# Management of atherosclerotic cardiovascular disease risk in diabetes mellitus patients: a population-level observational cohort study in Wales

**DOI:** 10.1093/ehjopen/oeaf158

**Published:** 2025-12-10

**Authors:** Daniel King, Ashley Akbari, Mike B Gravenor, Mathew Lawrence, Clive Weston, Sam Rice, Chris Hopkins, Leighton Phillips, Julian Halcox, Daniel E Harris

**Affiliations:** Faculty of Medicine, Health and Life Science, Swansea University Medical School, Swansea University, Singleton Park, Swansea SA2 8PP, UK; Faculty of Medicine, Health and Life Science, Swansea University Medical School, Swansea University, Singleton Park, Swansea SA2 8PP, UK; Faculty of Medicine, Health and Life Science, Swansea University Medical School, Swansea University, Singleton Park, Swansea SA2 8PP, UK; Tritech Institute, Hywel Dda University Health Board, Llanelli SA14 9TD, UK; Cardiology Department, Hywel Dda University Health Board, Glangwili Hospital, Carmarthen SA31 2AF, UK; Tritech Institute, Hywel Dda University Health Board, Llanelli SA14 9TD, UK; Tritech Institute, Hywel Dda University Health Board, Llanelli SA14 9TD, UK; Tritech Institute, Hywel Dda University Health Board, Llanelli SA14 9TD, UK; Faculty of Medicine, Health and Life Science, Swansea University Medical School, Swansea University, Singleton Park, Swansea SA2 8PP, UK; Department of Cardiology, Swansea Bay University Health Board, Singleton Hospital, Sketty Lane, Swansea SA2 8QA, UK; Faculty of Medicine, Health and Life Science, Swansea University Medical School, Swansea University, Singleton Park, Swansea SA2 8PP, UK; Tritech Institute, Hywel Dda University Health Board, Llanelli SA14 9TD, UK; Pharmacy Department, Hywel Dda University Health Board, Prince Phillip Hospital, Llanelli SA14 8QF, UK

**Keywords:** Lipids, Cholesterol, Atherosclerosis, Statin, Pharmacoepidemiology

## Abstract

**Aims:**

In patients with diabetes mellitus (DM) and atherosclerotic cardiovascular disease (ASCVD), or without ASCVD (primary prevention), the prescribing of lipid lowering therapy (LLT) is an established treatment strategy endorsed by clinical guidelines. This study aimed to document (i) trends in presentation of DM, (ii) treatment, monitoring and achievement of target low-density lipoprotein cholesterol (LDL-C) in DM with ASCVD, and (iii) ASCVD risk assessment and lipid treatment according to risk in the DM primary prevention setting.

**Methods and results:**

A retrospective observational population study including 282 581 DM patients using linked health-care data (2010–23) in Wales. The prevalence of DM (documented DM diagnosis in record prior to the beginning of the year) increased from 133 439 in 2010 to 183 948 in 2023 (6504 to 8200 per 100 000), along with increasing incidence (new diagnosis of DM documented in record during specific year) with 11 074 cases in 2010 (540 per 100 000 per year), increasing to 14 539 in 2023 (648 per 100 000 per year). The proportion of prevalent patients with established ASCVD prescribed LLT decreased from 87.5% to 81.8% (2010–23), testing of LDL-C decreased from 70.3% to 67.1%, and of those with documented lipids 41.0% achieved an LDL-C <1.8 mmol/L in 2010 increasing to 52.2% in 2023. Amongst DM without ASCVD, the proportion prescribed LLT decreased from 78.9% to 54.9% in those with chronic kidney disease (CKD) and from 70.7% to 55.6% in those without CKD. Considering DM without ASCVD or CKD (LLT is recommended according to 10-year CVD risk), only 44.2% of incident DM had a documented QRISK score in 2022 and of those with a 10-year risk >20%, only half were prescribed LLT.

**Conclusion:**

Increasing incidence and prevalence of DM, together with decreasing quality of risk factor management has the potential to lead to poorer health outcomes in the population if not addressed more effectively.

## Structured graphical abstract

**Structured graphical abstract:**
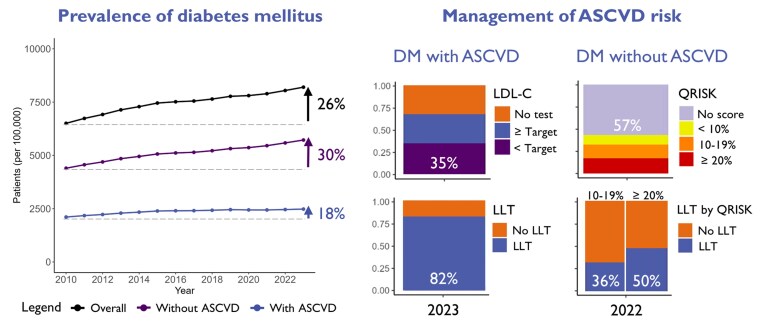

## Introduction

In patients with diabetes mellitus (DM) and established atherosclerotic cardiovascular disease (ASCVD), or without ASCVD (in all but the lowest risk of developing ASCVD), lipid lowering therapy (LLT) improves clinical outcomes and is a recognized treatment approach endorsed by all major clinical guidelines.^1–7^ In the United Kingdom, the National Institute for Health and Care Excellence (NICE) recommends LLT for the secondary prevention of ASCVD and in high-risk primary prevention patients including those with chronic kidney disease (CKD) or those with a 10-year CVD risk ≥10% (assessed using the QRISK score). A better understanding of the incidence and prevalence of DM in the population, together with trends in risk factor assessment and lipid management and factors associated with their delivery, will help determine not only population disease trends and the current and impending ‘therapeutic gap’ but also potential opportunities to improve ASCVD risk management.

The objectives of this study were to document (i) trends in the incidence and prevalence of DM, (ii) trends in the treatment, testing and control of lipids in those with established ASCVD, and (iii) CV risk assessment and prescribing of LLT according to risk in DM patients without ASCVD in the population of Wales, UK, since 2010.

## Methods

A retrospective observational cohort study was conducted using linked anonymized population-scale, individual-level electronic health record (EHR) data sources for patients with DM in Wales, United Kingdom, between 2010 and 2023, using the Secure Anonymized Information Linkage (SAIL) Databank.^8,9^

### Study inclusion and censor criteria

We included patients identified with DM in their primary care records between January 2000 and December 2023 (see Supplementary material for details of data sources). Patients with at least 1-year of follow-up were included in the documentation of trends in ASCVD risk management (see Supplementary material online, *Figure S1*).

Patients identified with DM between 1 January 2000 and 31 December 2009 who was alive during the study observation period (1 January 2010 and 31 December 2023) entered as prevalent cases in 2010. Patients first identified with DM before 2000 whose diagnoses were reconfirmed between 2000 and 2009 were also included as prevalent cases in 2010; otherwise they were excluded due to data quality concerns. Prevalent patients in 2010 were required to be registered as living in Wales, with a general practice providing data to SAIL at the start of the study period.

Patients diagnosed during the study period were included as incident cases and were considered incident for the year following diagnosis. Incident patients transitioned to prevalent from 1-year post-diagnosis until the year prior to censorship, to ensure a complete year of follow-up, or until the end of the study period. Incident patients were required to be resident in Wales, with a general practice providing data to SAIL for at least 90 days prior to first identification of DM.

Patients first identified with DM before the age of 18 entered the study in the year they turned 18. Patients were censored at the date of (i) death, (ii) moving to a General Practice that does not provide data to the SAIL Databank for a period > 90 days, or (iii) moved out of Wales for a period > 90 days.

### Medical history, assessment of QRISK score and demographic information

Age and deprivation quintiles were assigned at the index DM diagnosis date. Primary care EHR data were used to identify the following prior to inclusion census data: The presence of dementia, respiratory disease [including chronic obstructive pulmonary disease (COPD) or asthma], hypertension, chronic liver disease (including cirrhosis, fibrosis, chronic hepatitis, fatty liver, sclerosis of the liver, unspecified alcoholic liver damage or hepatic failure), smoking-status, and body mass index (BMI). Documentation of QRISK (version 2) 10-year risk score was identified from primary care records in the incident year.

### Characterizing ASCVD

Diagnoses of ASCVD (including ischaemic heart disease, stroke and/or peripheral arterial disease) were identified from either their primary or secondary care records (see Supplementary material online, *Tables S1*–S3 for diagnostic codes). ASCVD diagnoses made prior to diagnosis of DM were captured for incident patients. Among prevalent patients, diagnoses of ASCVD were identified throughout the follow-up and patients were considered as ‘With ASCVD’ from the year in which ASCVD was diagnosed. When describing prescription of LLT, patients were characterized as ‘With ASCVD’ from the year in which they first received an ASCVD diagnosis. When describing lipid testing, patients were characterized using the date of lowest recorded level. Recorded QRISK scores were only identified prior to any diagnosis of ASCVD.

### Characterizing CKD

Diagnoses of CKD were identified from recorded diagnoses of stage 3+ from primary care records and/or a recorded estimated glomerular filtration rate <60 mL/min/1.73 m^2^ from pathology records. Diagnoses were captured at any point prior to DM diagnosis for incident patients. For prevalent patients, diagnoses of CKD were captured throughout follow-up and used to characterize patients from the year in which they received the CKD diagnosis.

### Reporting of incidence and prevalence

The incidence of DM was reported as the number of patients receiving their first documented diagnosis of DM within each year (incident case) of the observation period. The prevalence of DM was reported as the number of patients alive with a diagnosis of diabetes already documented in the clinical record at the beginning of each year (prevalent case). If a patient was censored during a particular year, they were excluded from the prevalence count for the following year. We also reported the incidence and prevalence per 100 000 of the population; the denominator was calculated from the number of patients resident in Wales, registered with a primary care practice submitting records to SAIL, and aged ≥18 on 1st January each year.

### Lipid lowering therapy, testing and control of lipids

Prescriptions for LLT, including statins, ezetimibe, and fibrates were identified. LLT were classified as high intensity statin (HI-statin; atorvastatin ≥40 mg/d and rosuvastatin ≥20 mg/d), non-high-intensity (NI-statin; any other statin prescription), a combination of ezetimibe and/or fibrate with either HI- or NI-statin (combination statin), other treatments including ezetimibe and/or fibrate (other treatment) without a co-prescription of a statin, or no treatment. Documentation of lipid levels was identified from patient primary care records and pathology results.

For patients with ASCVD, in each year of the study, we reported the (i) prescribed LLT regimen and (ii) documentation of lipid testing and lipid levels for each patient with sufficient follow-up data (i.e. from the start to the end of each year for prevalent cases and a full year of follow up data after the date of first diagnosis for incident cases).

For patients without ASCVD and with CKD, we reported the prescribed LLT each year in prevalent and incident cases. Among incident cases without ASCVD, CKD or prescribed LLT in the year prior to diagnosis, we reported the (i) proportion with a documented QRISK score (in cases where more than one score was recorded, the highest was reported) and (ii) prescription of LLT in the year following diagnosis by recorded QRISK threshold (i.e. 10-year risk <10%, between 10 and 19%, over 20%).

### Statistical analysis

Multivariable binary logistic regression modelling was conducted to identify variables associated with (i) a prescription for HI-statin in patients with established ASCVD, (ii) achievement of an LDL-C < 1.8 mmol/L in patients with established ASCVD, (iii) a prescription of any LLT in patients with CKD (without ASCVD), (iv) documentation of QRISK 10-year risk score in patients without ASCVD, CKD or prior LLT prescription, and (v) prescribed LLT in the year following diagnosis in patients without ASCVD, CKD or prior LLT prescription. In each set of models, the analysis was performed for incident diabetics only, and the outcome was determined over the 12 months following entry into the cohort. In each case, a final model was determined by minimizing the Akaike information criterion. Odds ratios for the outcome were estimated for each variable in the final model. Analyses were carried out using R version 3.5. All scripts used to generate the findings presented in this study are available in a GitHub repository for others to access:

https://github.com/SwanseaUniversityDataScience/1483_Diabetes-ASCVD-Risk.

## Results

A total of 282 581 patients with DM were included in this study (see Supplementary material online, *Figure S1*), of whom 123 614 patients entered as a prevalent case and 158 967 were incident cases (*Table 1* and Supplementary material online, *Figure S1*).

**Table 1:** Cohort baseline characteristics at entry into study

	Prevalent	Incident^c^														Overall
		2010	2011	2012	2013	2014	2015	2016	2017	2018	2019	2020	2021	2022	2023	
	*n* = 123 614	*n* = 10 922	*n* = 10 619	*n* = 10 459	*n* = 10 941	*n* = 10 632	*n* = 11 453	*n* = 10 253	*n* = 10 174	*n* = 11 072	*n* = 12 419	*n* = 9740	*n* = 12 001	*n* = 13 764	*n* = 14 518	*n* = 282 581
**Sex**																
Male	67 597 (54.7%)	5983 (54.8%)	5844 (55%)	5824 (55.7%)	5877 (53.7%)	5724 (53.8%)	6139 (53.6%)	5565 (54.3%)	5496 (54%)	5875 (53.1%)	6619 (53.3%)	5227 (53.7%)	6347 (52.9%)	7378 (53.6%)	7806 (53.8%)	153 301 (54.3%)
**Age (years)**																
Entry to study	64.2 (15.3)	59.3 (14.9)	59.3 (15.0)	59.1 (15.1)	59.4 (15.0)	59.4 (15.1)	59.4 (15.6)	58.3 (16.0)	58.0 (16.0)	58.4 (15.9)	58.9 (16.1)	57.5 (16.2)	57.2 (15.8)	58.2 (15.9)	57.9 (16.1)	61.0 (15.7)
**Presence of ASCVD**																
	38 354 (31%)	2451 (22.4%)	2451 (23.1%)	2367 (22.6%)	2482 (22.7%)	2546 (23.9%)	2758 (24.1%)	2306 (22.5%)	2248 (22.1%)	2388 (21.6%)	2810 (22.6%)	2071 (21.3%)	2482 (20.7%)	2857 (20.8%)	2795 (19.3%)	73 366 (26%)
**Comorbidities**																
Respiratory Disease	23 256 (18.8%)	2092 (19.2%)	2078 (19.6%)	2054 (19.6%)	2327 (21.3%)	2299 (21.6%)	2555 (22.3%)	2311 (22.5%)	2330 (22.9%)	2533 (22.9%)	2958 (23.8%)	2300 (23.6%)	2794 (23.3%)	3097 (22.5%)	3309 (22.8%)	58 293 (20.6%)
Dementia	2210 (1.8%)	95 (0.9%)	104 (1.0%)	105 (1.0%)	107 (1.0%)	90 (0.8%)	130 (1.1%)	100 (1.0%)	102 (1.0%)	102 (0.9%)	146 (1.2%)	124 (1.3%)	96 (0.8%)	130 (0.9%)	143 (1.0%)	3784 (1.3%)
CKD (stage 3+)^b^	23 342 (18.9%)	1387 (12.7%)	1502 (14.1%)	1826 (17.5%)	2070 (18.9%)	2224 (20.9%)	2427 (21.2%)	2105 (20.5%)	2255 (22.2%)	2483 (22.4%)	3138 (25.3%)	2425 (24.9%)	2892 (24.1%)	3398 (24.7%)	3595 (24.8%)	57 069 (20.2%)
Liver Disease	2171 (1.8%)	188 (1.7%)	195 (1.8%)	193 (1.8%)	261 (2.4%)	234 (2.2%)	295 (2.6%)	255 (2.5%)	289 (2.8%)	357 (3.2%)	474 (3.8%)	431 (4.4%)	586 (4.9%)	642 (4.7%)	743 (5.1%)	7314 (2.6%)
Heart Failure	6743 (5.5%)	385 (3.5%)	377 (3.6%)	336 (3.2%)	383 (3.5%)	396 (3.7%)	497 (4.3%)	358 (3.5%)	382 (3.8%)	369 (3.3%)	438 (3.5%)	350 (3.6%)	433 (3.6%)	465 (3.4%)	482 (3.3%)	12 394 (4.4%)
Hyper-tension	77 622 (62.8%)	5524 (50.6%)	5265 (49.6%)	5054 (48.3%)	5234 (47.8%)	4965 (46.7%)	5245 (45.8%)	4523 (44.1%)	4405 (43.3%)	4886 (44.1%)	5633 (45.4%)	4168 (42.8%)	4984 (41.5%)	5873 (42.7%)	6090 (41.9%)	149 471 (52.9%)
**Smoker status**																
Active smoker^a^	21 288 (17.2%)	2300 (21.1%)	2175 (20.5%)	2262 (21.6%)	2227 (20.4%)	2268 (21.3%)	2361 (20.6%)	2116 (20.6%)	2052 (20.2%)	2205 (19.9%)	2325 (18.7%)	1957 (20.1%)	2379 (19.8%)	2539 (18.4%)	2598 (17.9%)	53 052 (18.8%)
**Weight**																
Obese^a^	63 867 (51.7%)	6326 (57.9%)	6244 (58.8%)	6252 (59.8%)	6565 (60%)	6263 (58.9%)	6824 (59.6%)	6109 (59.6%)	6179 (60.7%)	6766 (61.1%)	7614 (61.3%)	5981 (61.4%)	7600 (63.3%)	8406 (61.1%)	8800 (60.6%)	159 796 (56.5%)

^a^See Supplementary material online, *Table S4* for further cohort characteristics of weight, smoking status, and deprivation quintile.

^b^CKD stage 3+ captured as primary care diagnosis or eGFR <60.

^c^Incident cases are patients receiving their first documented diagnosis of DM within the given year. Prevalent cases are patients alive who with a diagnosis of DM documented in a prior year.

The mean age of incident cases in 2010 was 59.2 (SD ±15.3) years, decreasing to 57.9 (SD ±16.1) in 2023 (*Table 1*; see Supplementary material online, *Tables S5* and *S6* for characteristics of prevalent and incident cases presenting with and without ASCVD).

### Temporal trends in diabetes mellitus

### Prevalence

**Figure 1:**
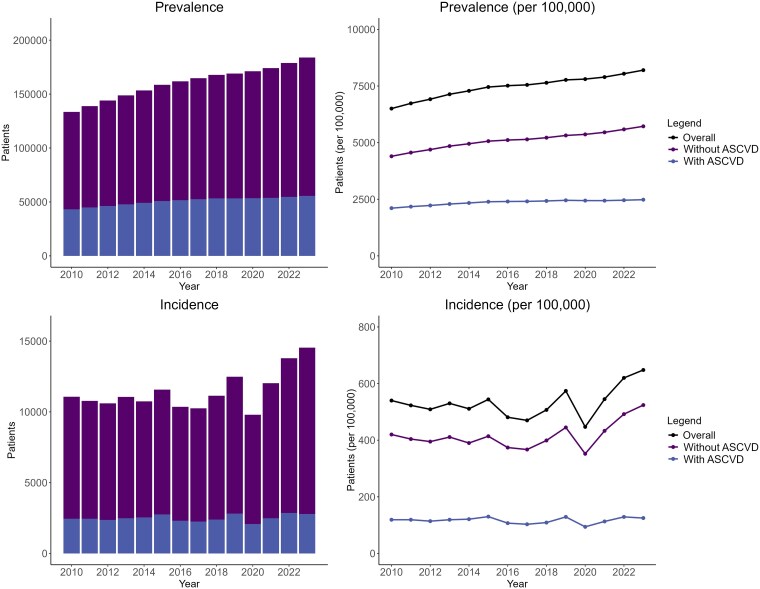
Prevalence and incidence of diabetes mellitus in patients with and without atherosclerotic cardiovascular disease. Prevalence (top) and incidence (bottom) from 2010 to 2023. Absolute numbers (left) and per 100 000 of the population (right).

The population prevalence of DM increased from 133 439 (6504 per 100 000) in 2010 to 183 948 (8200 per 100 000) in 2023 (*Figure 1* and Supplementary material online, *Table S7*). Over the same period the number of these patients with ASCVD increased from 43 237 (2107 per 100 000) to 55 640 (2480 per 100 000) and the number of patients without ASCVD increased from 90 202 (4397 per 100 000) to 128 308 (5720 per 100 000).

### Incidence

In 2010, there were 11 074 incident cases of DM (540 per 100 000 per year), increasing to 14 539 (648 per 100 000 per year) in 2023 (see Supplementary material online, *Table S7*). During 2020, the first year of the COVID-19 pandemic, there were fewer new diagnoses of DM, 9794 (447 per 100 000 per year), with a subsequent increase [12 029 (545 per 100 000 per year) in 2021; 13 791 (620 per 100 000) in 2022].

### Trends in LLT prescribing, lipid testing and control in patients with ASCVD

Of the 282 581 patients with DM, 251 210 patients had at least 1 year of follow up data (see Supplementary material online, *Table S8*).

### Prescribed lipid lowering therapy in patients with ASCVD

**Figure 2:**
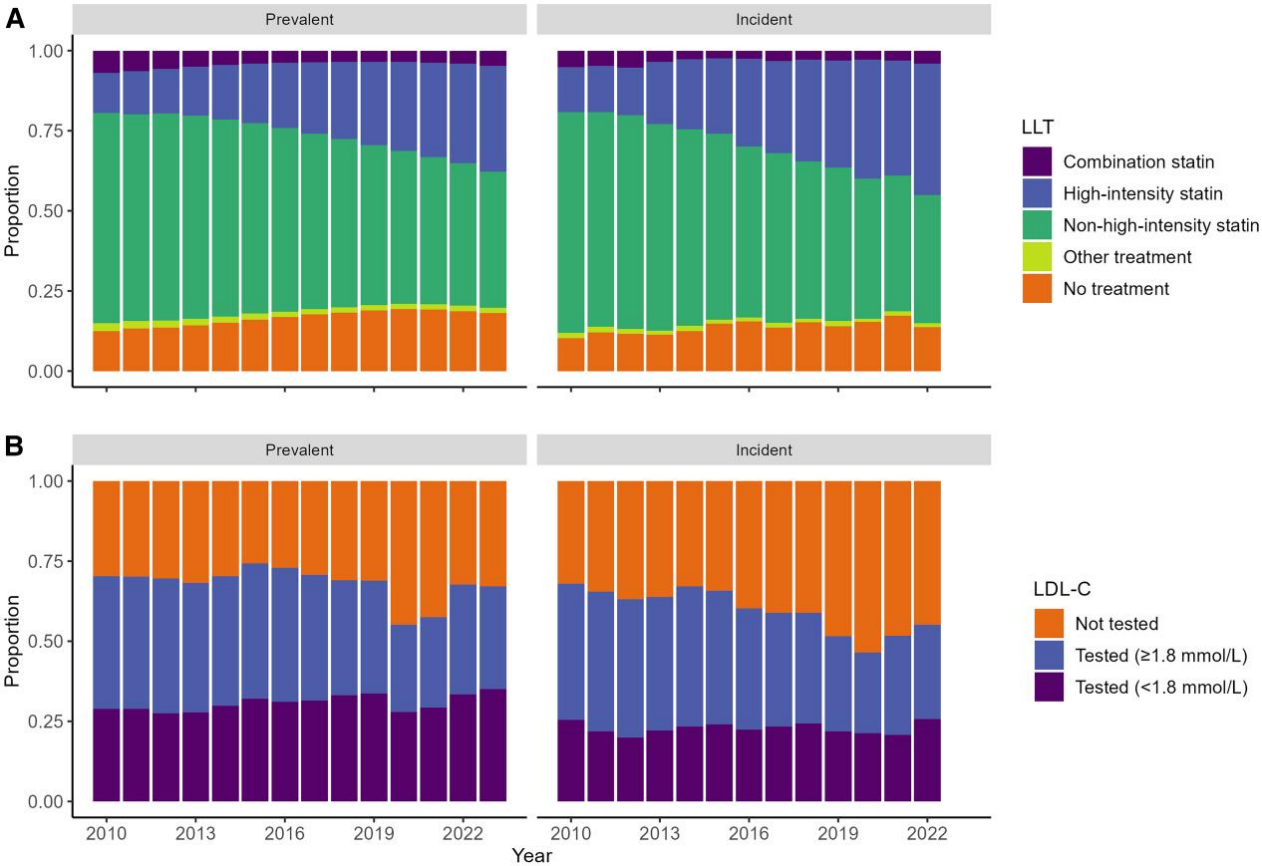
Lipid lowering treatment (A) and low-density-lipoprotein cholesterol testing and control (B) in prevalent cases and incident cases with established ASCVD during each year of the study period.

The proportion of prevalent patients with established ASCVD who were prescribed LLT decreased from 87.5% in 2010 to 81.8% in 2023. The proportion of incident cases with established ASCVD prescribed LLT decreased from 89.7% in 2010 to 82.8% in 2021, recovering in 2022 to 86.3%. (*Figure 2* and Supplementary material online, *Table S8* for further prescription details).

Male sex and being a current or former smoker were independently associated with a greater likelihood of a prescription for HI-statin, whereas CKD, dementia, liver disease and hypertension were associated with a lower likelihood of HI-statin prescription according to multivariable regression analyses in the incident population with ASCVD (see Supplementary material online, *Table S9*).

### Lipid testing in patients with ASCVD

Amongst the prevalent patients with ASCVD, documentation of LDL-C increased from 70.3% in 2010 to 74.3% in 2015, but fell thereafter to 68.9% by 2019. During the pandemic, only 55.2% of patients had a documented LDL-C level in 2020 and only 57.5% in 2021, with partial recovery to 67.1% with documented levels in 2023 (*Figure 2* and Supplementary material online, *Table S10*).

The proportion of incident cases with established ASCVD with a documented LDL-C result decreased from 67.9% in 2010 to 57.9% in 2018 (the last complete year of follow-up prior to the pandemic). In 2019, (51.6%) of incident patients had a documented LDL-C result; 46.4% in 2020 with a slight recovery to 55.1% by 2022.

Trends in non-HDL-C documentation closely mirrored those observed in LDL-C documentation in both prevalent and incident groups with established ASCVD (see Supplementary material online, *Figure S2* and *Table S11*).

### Lipid control in patients with ASCVD

**Figure 3:**
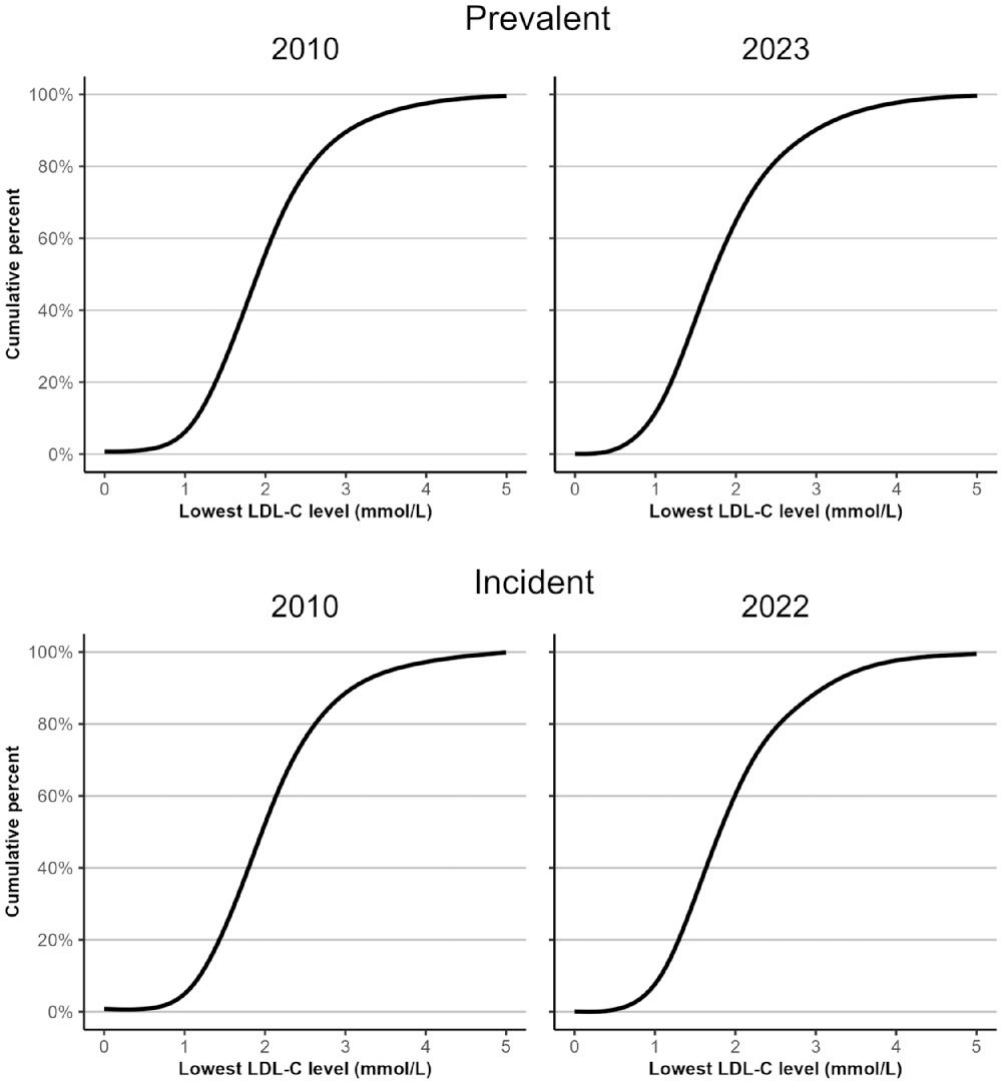
Distribution of lowest achieved low-density-lipoprotein cholesterol level among prevalent patients with ASCVD in 2010 and 2023 (top) and incident patients with ASCVD in 2010 and 2022 (bottom).

In 2010, only 41.0% of the prevalent patients with ASCVD, who also had documented LDL-C levels achieved an LDL-C of <1.8 mmol/L, increasing to 52.2% by 2023 (*Figures 2* and *3* and Supplementary material online, *Table S10*). As a proportion of the prevalent population with ASCVD (also including those without documented LDL-C) only 28.8% had documentation of an LDL-C <1.8 mmol/L in 2010, increasing to 35.0% by 2023 (*Figure 2* and Supplementary material online, *Table S10*).

In 2010, 37.5% of incident patients with ASCVD (and documented LDL-C levels) achieved an LDL-C of <1.8 mmol/L, increasing to 46.7% in 2022 (*Figures 2* and *3* and Supplementary material online, *Table S10*). As a proportion of the incident population (including those without documented LDL-C) only 25.5% had an LDL-C <1.8 mmol/L in 2010% and 25.7% in 2022 (*Figure 2* and Supplementary material online, *Table S10*).

Across the same period there was less of an improvement seen in the control of non-HDL-C. In 2010, 32.9% of the prevalent patients (with documented levels) achieved a non-HDL-C <2.6 mmol/L increasing to 34.8% in 2023 (see Supplementary material online, *Figure S2* and *Table S11*). Amongst the incident cases with documented levels in 2010, 28.5% achieved a non-HDL-C < 2.6 mmol/L and 25.8% in 2022.

Male sex, increasing age, CKD, and those prescribed LLT (compared with no LLT prescription) were independently associated with a greater likelihood of achieving an LDL-C level of <1.8 mmol/L, whereas a diagnosis of dementia was associated with greater likelihood of an LDL-C level of ≥1.8 mmol/L according to multivariable regression analyses (see Supplementary material online, *Table S12*).

### Trends in LLT prescribing and CVD risk assessment in patients without ASCVD

Of the 251 210 diabetic patients with at least 1 year follow-up data across the study period, there were 79 624 prevalent patients with DM and without a diagnosis of ASCVD in 2010, increasing to 111 097 in 2022. Within this group the proportion prescribed LLT decreased from 71.6% in 2010 to 59.6% in 2023 (see Supplementary material online, *Table S8* for further details of prescribed LLT).

In 2010 there were 7216 incident cases without ASCVD increasing to 9260 cases in 2022. Within this group, the proportion prescribed any LLT decreased from 53.7% in 2010 to 39.0% in 2021% and 44.7% in 2022 (see Supplementary material online, *Table S8*).

### LLT prescribing in patients without ASCVD and with CKD

**Figure 4:**
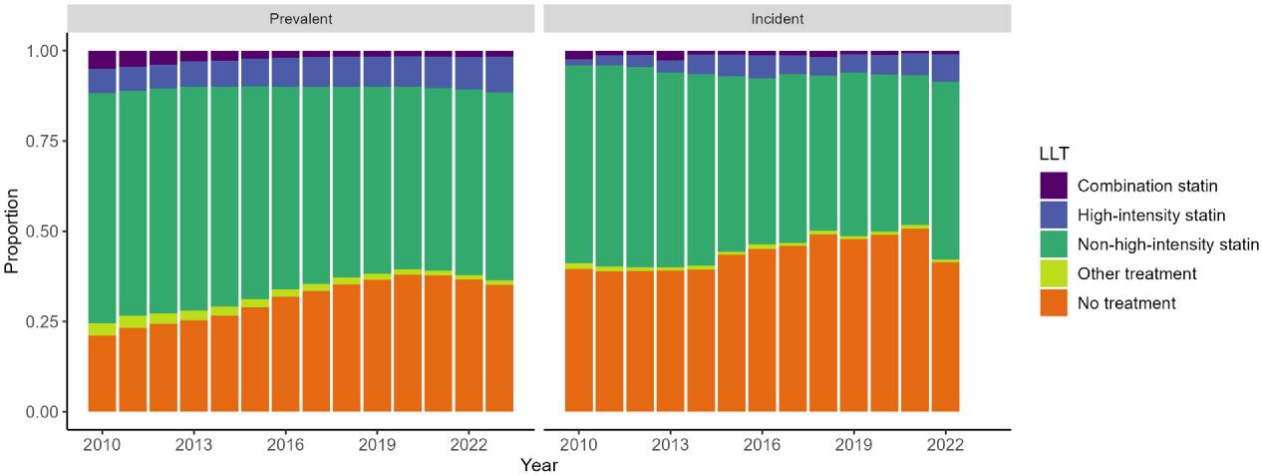
Lipid lowering treatment in prevalent cases and incident cases with chronic kidney disease (stage 3+) but without ASCVD during each year of the study period.

Of the prevalent diabetic patients without ASCVD, the number with a diagnosis of CKD increased from 10 402 to 12 838 (2010–2023). Of this group, the proportion prescribed LLT decreased from 78.9% in 2010 to 64.9% in 2023 (*Figure 4* and Supplementary material online, *Table S13* for further prescribing data).

Of incident diabetic patients without ASCVD, the number also with CKD increased from 625 in 2010 to 1840 in 2022. Of these patients, the proportion prescribed LLT decreased from 60.5% in 2010 to 49.3% in 2021, increasing to 58.6% in 2022 (*Figure 4* and Supplementary material online, *Table S13*).

Male sex, hypertension, those overweight or obese (vs. normal weight), and smoking history (compared with non-smokers) were independently associated with a prescription for statin therapy in diabetics with CKD and without ASCVD within the incident year, whereas less deprived socioeconomic quintiles (compared with those most deprived), dementia and liver disease were associated with lower likelihood or being prescribed statin therapy according to multivariable regression analyses (see Supplementary material online, *Table S14*).

### LLT prescribing in prevalent patients without a diagnosis of ASCVD or CKD

The number of prevalent DM patients without ASCVD or CKD increased from 70 156 in 2010 to 80 168 in 2022. Of these, the proportion prescribed LLT decreased from 70.7% in 2010 in 55.6% in 2023 (see Supplementary material online, *Figure S3* and *Table S15* for further prescription data).

### QRISK documentation in incident patients without a diagnosis of ASCVD or CKD or prior LLT prescription

**Figure 5:**
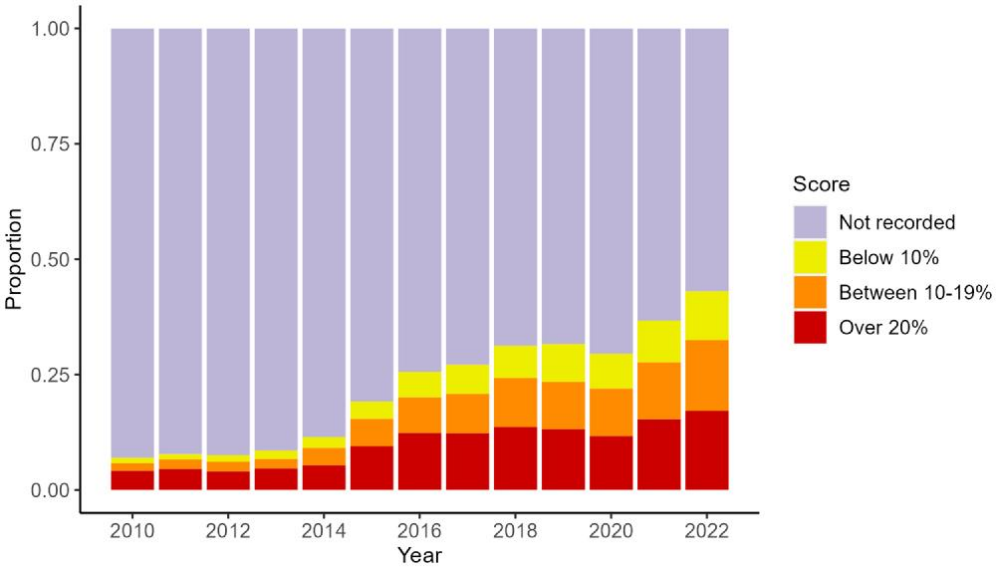
Proportion of incident diabetics (not prescribed LLT prior to diagnosis and without chronic kidney disease (stage 3+) or ASCVD) with documented QRISK 10-year risk score by QRISK threshold and year.

The number of patients with incident DM not prescribed LLT prior to diagnosis and without ASCVD or CKD increased from 4439 to 5476 (see Supplementary material online, *Table S16*). Among these patients, guidelines recommend assessment of ASCVD risk to direct the decision to prescribe LLT. In 2010, only 309 (7.0%) had a documented QRISK score increasing to 2358 (43.1%) in 2022. Of these patients with a recorded QRISK score the proportion with a documented risk <10% increased from 1.2% in 2010 to 10.6% in 2022; those with a documented risk of 10–19% increased from 1.6% to 15.3% and those with a risk >20% increased from 4.1% to 17.2% across the same period (*Figure 5* and Supplementary material online, *Table S16*).

Factors independently associated with a greater likelihood of a QRISK score being documented in the incident year included a history of hypertension, liver disease, the year of diagnosis of DM (later years more likely) and patients in less deprived socioeconomic quintiles (compared with most deprived) whereas dementia and respiratory disease were associated with a lower likelihood of LLT prescribing according to multivariable regression analyses (see Supplementary material online, *Table S17*).

### LLT prescribing according to QRISK in incident patients without a diagnosis of ASCVD or CKD or prior LLT prescription

**Figure 6:**
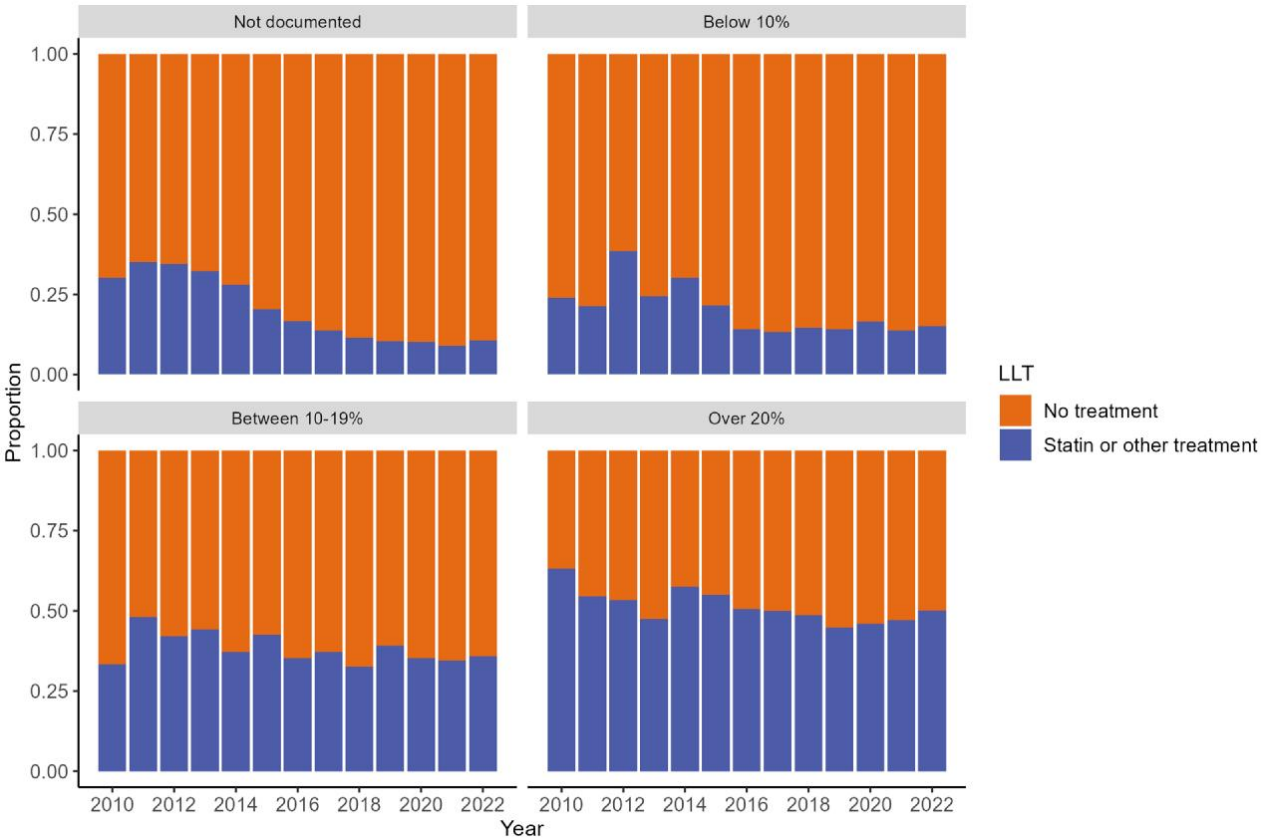
Prescribed lipid lowering therapy for incident patients [not prescribed LLT prior to diagnosis and without chronic kidney disease (stage 3+) or ASCVD] in the year following diagnosis by QRISK documentation and year.

Among the incident population (not prescribed LLT prior to diagnosis and without ASCVD or CKD) in 2010 with a recorded QRISK of >20%, 63.2% were prescribed LLT decreasing to 50.2% in 2022; in those with a QRISK 10–19%, LLT prescribing increased from 33.3% in 2010 to 35.9% in 2022 and in those with a QRISK < 10%, 23.9% were prescribed LLT, decreasing to 15.1% in 2022. (*Figure 6* and Supplementary material online, *Table S18*).

In 2010, 4158 (38.1%) had no QRISK documented, decreasing to 3248 (22.4%) in 2022. Of these patients, 1257 (30.2%) were prescribed LLT in the year following diagnosis in 2010, decreasing to 345 (10.6%) in 2022 (*Figure 5* and Supplementary material online, *Table S18*).

Factors independently associated with a greater likelihood of LLT prescription in the incident year included documented QRISK score, male sex, history of hypertension, age at diagnosis of DM (older patients more likely), and current smokers (compared with non-smokers) whereas diagnosis year (later years less likely), history of dementia and less deprived socioeconomic quintiles (compared with most deprived) were associated with a lower likelihood according to multivariable regression analyses (see Supplementary material online, *Table S19*).

## Discussion

Across the study period, we observed an increase in the incidence and prevalence of DM, and a concurrent decrease in the proportion of patients prescribed LLT (in DM patients both with and without ASCVD). Clinical documentation of LDL-C (and non-HDL-C) fell across the study period; but although a greater portion of those with ASCVD with documented lipids achieved acceptable LDL-C control, there was less of an improvement in non-HDL-C control in these patients.

Over the 13 years of this study, we observed a 37.9% increase in the number of patients with DM, equating to a 26.1% relative increase in the proportion of the population living with DM, having taken population growth into account. The reported population prevalence in this study is similar to that reported by the Public Health Wales,^10^ Public Health England,^11^ and other epidemiological reports, including other higher income nations.^12^

Overall, there was a fall in the mean age of diagnosis of DM. Not only is this important risk factor associated with a decreased healthy life^13,14^ due to vascular complications, but an earlier onset will likely reduce healthy life expectancy, with earlier development of these complications unless effectively managed.^15^ Indeed, the combination of an increasing prevalence, and falling age at diagnosis together with the less effective general provision of risk-factor management observed in this study is of particular concern regarding the future cardiovascular health of this high-risk population.

Amongst those patients with ASCVD, we observed increasing HI-statin and decreasing NI-statin prescribing. This suggests that statin intensification may well account for most of the improvement in LDL-C control (where documented) rather than wider provision of LLT, given almost 1 in 5 of these patients not being prescribed LLT during the latter years of our study. This low achievement of guideline directed LDL-C targets and underutilization of LLT, is certainly not unique to Wales and has also recently been documented in other very high-risk ASCVD populations.^16–22^

While fewer patients with ASCVD had a documented LDL-C level over the study period, a greater proportion of these tested patients were at target. However, there was less of a concurrent improvement in the proportion of patients achieving non-HDL targets. This is particularly pertinent for patients with type 2 DM in whom their dyslipidaemia is typically characterized by elevated triglycerides, low HDL and smaller denser LDL-C with proportionally higher levels of atherogenic ApoB lipoproteins not in the LDL sub-fraction, reflecting a pro-atherogenic lipid phenotype.^1^

Previous studies have explored the impact of the COVID-19 pandemic on CVD diagnosis, risk management, and outcomes, including describing the decrease in prescriptions for CVD risk management with modelled estimates on increase in CVD events.^23^ Although this study was not designed to assess the interactions between DM diagnosis, risk management, and outcomes or the impact of COVID-19 on these factors, the data in this study show an appreciable drop in incident diagnoses of DM, and a decline in lipid testing and prescribing of LLT (in incident ASCVD cases) during the pandemic. While this is of concern, data from this study highlight the gap in evidenced based risk factor management across the study period incorporating the COVID-19 pandemic and subsequent years.

Within the group without ASCVD we provided a detailed assessment of lipid management in those with comorbid CKD. In the UK, NICE clinical guidelines recommend statin treatment in DM and CKD without the need for CVD risk assessment. Despite this guidance, prescribing was low with over a third of this group not receiving LLT in 2023.

In patients without ASCVD or CKD (or other high-risk features), clinical guidelines recommend evaluation of risk to guide decisions to prescribe statins for primary prevention. Since 2012 NICE has recommended the use of QRISK2^24^ to assess CVD risk. In 2014 NICE lowered the threshold for initiation of statins from 20% to 10% 10-year ASCVD risk.^25^ Across the period of this study, documentation of QRISK improved. However, in those with a documented 10-year risk >20%, the proportion who were prescribed LLT decreased, with only a half of such cases receiving any LLT in 2022.

In the multivariable analyses, we note that in the cohort with ASCVD, females were less likely to be prescribed HI-statin and less likely to have controlled lipid levels documented; in the cohort without ASCVD and with CKD, females were also less likely to be prescribed statin therapy; also in the cohort without ASCVD or CKD females were less likely to be prescribed LLT, although sex was not a predictor of QRISK documentation. These findings provide further evidence of the gender disparity in CVD risk management and highlight a persistent need to address this gap.^26–28^

We were interested to note that deprivation status was for the most part not significantly associated with effectiveness of testing treatment and control of lipids in our analysis. We have classified this using the well validated Welsh Index of Multiple Deprivation, the Welsh Government’s official measure of relative deprivation for small areas in Wales. This identifies areas with the highest concentrations of several different types of deprivation, ranking all small areas in Wales from most to least deprived, updated every 4 to 5 years. These data suggest that despite the well-recognized adverse socioeconomic gradient in the development of DM and ASCVD,^29^ the management of lipids in the population is relatively equitable across the population, once these conditions are diagnosed.

### Strengths and limitations

To the best of our knowledge, this is the first study to describe contemporaneous national trends in the incidence and prevalence of DM together with lipid management in both primary and secondary ASCVD prevention populations. The SAIL databank provides a highly representative sample for evaluation, comprising the primary care records of over 85% of the Welsh population, without systematic differences with regard to the geographical locations or demographic composition of the practices not included in the databank.

We included patients with type 1 and type 2 DM. Only 3% of the entire cohort were type 1 DM and due to disclosure and privacy protection processes when using the SAIL Databank in reporting small numbers, we did not analyse this cohort separately or exclude this very high-risk group, which faces a greatly reduced life expectancy compared with the wider population.^1,30–32^ Not all patients with DM are classified as T1 or T2 in their record. Furthermore, secondary prevention LLT recommendations are similar in T1 and T2 DM and the majority of T1 DM patients would be considered at a sufficiently high level of risk to indicate LLT. Furthermore, documentation of urinary albuminuria is relatively infrequent in the primary care record, we therefore did not formally include this in our classification of CKD, restricting our inclusion criteria to the eGFR threshold.

In those with ASCVD we reported achievement of the ESC/EAS LDL-C target of 1.8 mmol/L, which was guideline recommendation for most of the study period (2011–2019)^33,34^ before the more stringent target of <1.4 mmol/L was introduced in 2019.^2^ Clearly, a lower proportion of patients would have achieved this target. We did not report achievement of the latest NICE targets for secondary prevention (LDL-C ≤ 2.0 mmol/L or non-HDL-C ≤ 2.6 mmol/L) which were introduced in December 2023 at the end of observation period but could also be inferred from *Figure 3*.^5^

We also did not report UK-specific lipid targets for the prevention of ASCVD which differ between NICE and ESC guidelines. NICE recommends aiming for aiming for a greater than 40% reduction in non-HDL-C for primary prevention and ESC guidelines recommend an LDL-C < 3.0 mmol/L in low-risk patients, < 2.6 mmol/L in patients at moderate risk and <1.8 mmol/L in those at high risk (most recently <1.4 mmol/L). We could not be confident in calculating ASCVD risk (in the absence of documented risk) or determining whether (or indeed which of) the non-HDL-C results were true pre-treatment levels from which to calculate the percentage reduction with treatment.

No patients prescribed the proprotein convertase subtillsin/kexin type 9 (PCSK9) monoclonal antibodies (MAb) evolocumab or alirocumab were identified. These agents were approved for use within the UK National Health Service in 2016. Similarly, no patients prescribed bempedoic acid or inclisiran (approved for use in NHS Wales in 2020 and 2021, respectively) were identified. Although the prescription of all these treatments has mainly been through specialist hospital outpatient services, (for whom their data was not routinely available for this study). The use of these agents is restricted in the Welsh NHS; PCSK9 Mab and inclisiran are only approved for use in ASCVD with LDL ≥4.0 mmol/L or ≥3.5 mmol/L in those with recurrent ASCVD events, and bempedoic acid is only approved for use in patients where statin therapy is contraindicated or not tolerated and ezetimibe therapy alone does not control LDL-C, thus the uptake of these agents has been low thus far in Wales. This does suggest a considerable potential for improved control of LDL-C with wider access to and increased uptake of these agents in the future.

While the restrictions on use of these newer LLT may limit the generalizability of our results into other healthcare systems, the gap in implementation of evidenced based risk factor management has been widely reported across multiple healthcare systems. A further consideration is that the data for this study was obtained from the Welsh NHS, where the cost of healthcare including prescriptions is free at the point of delivery. This may mitigate potential barriers when comparing these data to other healthcare systems, where affordability may disadvantage individuals and populations.

In this study, we took a liberal approach to reporting whether patients were prescribed LLT, with only a single prescription required to be classified as receiving LLT, although the vast majority of patients classified as treated with LLT were in receipt of longer-term prescriptions. However, a detailed, time dependent prospective analysis of longer term LLT prescribing and target achievement was outside the scope of this study. It was also not possible in this study of anonymized real-world clinical records, to account for compliance or adherence to therapy which has frequently been reported to be low, particularly with statin therapy. Therefore, our results represent the best-case scenario, with the real-world use of LLT and longer-term therapeutic effectiveness likely to be lower than that reported here.

Due to the nature of using routinely collected EHR data, it is not possible to determine the specific reasons why so few patients were prescribed guideline directed LLT. However, we offer several suggestions. Patients with DM often have care directed by multiple teams within secondary and primary care. The responsibility for risk factor management often falls between these clinical settings; with the lack of time available to clinicians, or robust systems/pathways to support patient care, or reimbursement/investment for implementing treatment guidelines opportunities to optimize risk factor management is often missed, as these data demonstrate.

We acknowledge that medication compliance is often low for chronic conditions, especially in the ‘preventative’ setting. Indeed ‘statin intolerance’, whether perceived or real is frequently encountered by clinicians. However, this study reported low prescribing of LLT in incident cases with high QRISK scores who had not previously (in the year prior) been prescribed LLT. We suspect that the observed gap in the prescribing of LLT is predmoninatly due to missed opportunity in a healthcare system where preventative care is not adequately prioritized with limited time available and resource availability competing service demands compromising optimal delivery of preventive care.

Considering the rising prevalence and declining provision of effective risk factor management in DM and those with or at risk of ASCVD from the wider population,^21^ digital technology that supports the identification of high-risk patients with treatment gaps may support effective use of limited clinician time. Clinical pathways delivering preventative care that is not restricted by diversion of activity to acute and symptomatic conditions should be evaluated. At a population level, collation of data to identify specific groups and/or geographies where risk factor provision is least effective may support the targeting of resources to where the need is greatest.

Although our study has not examined the relationship between testing treatment and control of lipids and clinical outcomes—a topic for future evaluation—the relationship between LDL-C levels and clinical outcomes as well as the incremental impact of LLT escalation and clinical outcomes is well recognized from epidemiological and clinical trial meta-analyses. Therefore, our findings could serve as a proxy for likely suboptimal outcomes at a population level and for what may be achievable with improved lipid management in this high-risk population.

## Conclusion

This study describes the rapid increase in the prevalence of DM and decreased provision of guideline recommended, prognostically beneficial LLT. Failure to adequately address the behaviours contributing to this rise in DM and providing evidenced based risk-factor management in those with DM has the potential to lead to further increases in prevalence and poor outcomes in the population if not addressed more effectively as an urgent priority.

## Lead author biography


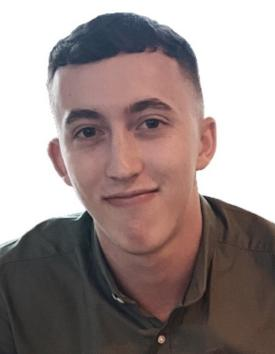


Daniel King is a researcher and data scientist at Swansea University. With a background in biomechanics and data analytics, he is pursuing a PhD in population and health data science. His research focuses on population-level cardiovascular risk management with a strong interest in utilizing statistical modeling techniques for optimizing risk prevention strategies.

## Supplementary Material

oeaf158_Supplementary_Data

## Data Availability

The data used in this study are available in the SAIL Databank at Swansea University, Swansea, UK. All proposals to use SAIL data are subject to review by an independent Information Governance Review Panel (IGRP). Before any data can be accessed, approval must be given by the IGRP. The IGRP gives careful consideration to each project to ensure proper and appropriate use of SAIL data. When access has been approved, it is gained through a privacy-protecting trusted research environment (TRE). SAIL has established an application process to be followed by anyone who would like to access data via https://saildatabank.com/data/apply-to-work-with-the-data. This project was approved by the IGRP (SAIL project number 1483). Participant consent was not required by the IGRP as all data was anonymized prior to the study.
