# Supplementary material for: Management of atherosclerotic cardiovascular disease risk in diabetes mellitus patients: a population-level observational cohort study in Wales
Source: Eur Heart J Open. 2025 Dec 10;5(6):oeaf158. doi: 10.1093/ehjopen/oeaf158 (PMC12730873; doi:10.1093/ehjopen/oeaf158)
Supplement: oeaf158_Supplementary_Data [file oeaf158_supplementary_data.docx]

**Supplementary Material**

[Data sources 4](#_Toc203644447)

[Smoking status 4](#_Toc203644448)

[Body mass index 4](#_Toc203644449)

[Diagnostic codes 6](#_Toc203644450)

[Supplementary Table 1. Read codes (version 2) for diagnoses of diabetes mellitus. 6](#_Toc203644451)

[Supplementary Table 2a. Read codes (version 2) for diagnoses of ischaemic heart disease. 11](#_Toc203644452)

[Supplementary Table 2b. Read codes (version 2) for diagnoses of peripheral arterial disease. 14](#_Toc203644453)

[Supplementary Table 2c. Read codes (version 2) for diagnoses of stroke. 24](#_Toc203644454)

[Supplementary Table 3a. ICD-10 codes for diagnoses of ischaemic heart disease. 26](#_Toc203644455)

[Supplementary Table 3b. ICD-10 codes for diagnoses of peripheral arterial disease. 27](#_Toc203644456)

[Supplementary Table 3c. ICD-10 codes for diagnoses of stroke. 28](#_Toc203644457)

[Supplementary Figure 1. Inclusion criteria for study cohort. 29](#_Toc203644458)

[Supplementary Table 4. Cohort baseline additional characteristics at entry into study, continued. 30](#_Toc203644459)

[Supplementary Table 5. Cohort baseline characteristics at entry into study for diabetics without ASCVD. 32](#_Toc203644460)

[Supplementary Table 6. Cohort baseline characteristics at entry into study for diabetics with ASCVD. 34](#_Toc203644461)

[Supplementary Table 7. Prevalence and Incidence of diabetes mellitus by presence of ASCVD across the study period. 36](#_Toc203644462)

[Supplementary Table 8. Prescribed lipid lowering therapy across the study period, in those with or without ASCVD. 37](#_Toc203644463)

[Supplementary Table 9. Multivariable logistic regression analysis of variables associated with a prescription for high-intensity statin therapy in diabetics with ASCVD within the incident year. 41](#_Toc203644464)

[Supplementary Table 10. Number and proportion of diabetics with ASCVD with documented and controlled low density lipoprotein cholesterol by year. 42](#_Toc203644465)

[Supplementary Figure 2. Proportion of diabetics with ASCVD with documented and controlled non-high density lipoprotein cholesterol by year. 43](#_Toc203644466)

[Supplementary Table 11. Proportion of diabetics with ASCVD with documented and controlled non-high density lipoprotein cholesterol by year. 44](#_Toc203644467)

[Supplementary Table 12. Multivariable logistic regression model to predict the likelihood of achieving low density lipoprotein cholesterol control (<1.8 mmol/L) in diabetics with ASCVD during incident year. 45](#_Toc203644468)

[Supplementary Table 13. Prescribed lipid lowering therapy across the study period in diabetics with chronic kidney disease (stage 3+) and without ASCVD. 46](#_Toc203644469)

[Supplementary Table 14. Multivariable logistic regression model to predict the likelihood of being prescribed lipid lowering therapy to diabetics with chronic kidney disease (stage 3+) and without ASCVD during incident year. 48](#_Toc203644470)

[Supplementary Figure 3. Prescribed lipid lowering therapy for prevalent diabetics (without chronic kidney disease (stage 3+) or ASCVD) across the study period. 49](#_Toc203644471)

[Supplementary Table 15. Prescribed lipid lowering therapy for prevalent diabetics (without chronic kidney disease (stage 3+) or ASCVD) across the study period. 50](#_Toc203644472)

[Supplementary Table 16. Number and proportion of diabetics (not prescribed lipid lowering therapy prior to diagnosis and without chronic kidney disease (stage 3+) or ASCVD) with documented QRISK 10-year risk score by QRISK threshold and year. 51](#_Toc203644473)

[Supplementary Table 17. Multivariable logistic regression model to predict the likelihood of having QRISK score documented for diabetics (not prescribed lipid lowering therapy prior to diagnosis and without chronic kidney disease (stage 3+) or ASCVD) during incident year. 52](#_Toc203644474)

[Model covariates determined by minimising the Akaike information criterion – no covariates removed. 52](#_Toc203644475)

[Supplementary Table 18. Prescribed lipid lowering therapy for incident diabetics (not prescribed lipid lowering therapy prior to diagnosis and without chronic kidney disease (stage 3+) or ASCVD) in the year following diagnosis by QRISK documentation and year. 53](#_Toc203644476)

[Supplementary Table 19. Multivariable logistic regression model to predict the likelihood of being prescribed lipid lowering therapy in the year following diagnosis for incident diabetics (not prescribed lipid lowering therapy prior to diagnosis and without chronic kidney disease (stage 3+) or ASCVD). 55](#_Toc203644477)

**Data sources**

Several data sources in the Secure Anonymised Information Linkage (SAIL) Databank were linked: the Patient Episode Database for Wales (PEDW), which records hospital admission and discharge dates, diagnoses and operational procedures, demographic data, and death date if relevant for the population of Wales; the Welsh Longitudinal General Practice (WLGP) data containing demographic, clinical, and prescribing data for about 85% of primary care practices across Wales; the Welsh Demographic Service Dataset (WDSD), which contains basic demographic information and history of individuals’ residence in Wales and registration with GP practices; and the Welsh Index of Multiple Deprivation (WIMD) 2019, an area-based deprivation measure.

**Smoking status**

Any patients recorded as a ‘smoker’, within their primary care record, prior to their first diabetes diagnosis were considered an ‘active smoker’. Those most recently recorded in primary care as an ‘ex-smoker’ were categorised as such. However, an ‘ex-smoker’ record was only valid 180 days after any previous ‘smoker’ record. Those with no recorded history of ‘smoker’ status were considered a ‘non-smoker’.

**Body mass index**

Body mass index (BMI), for each patient in the study cohort, was derived from primary and secondary care electronic health record data. To describe incident patients at entry into the study, the most recent record prior to their index diagnosis was used. For prevalent patients, who entered at the beginning of the study period, the most recent record prior to 1st January 2010 was used.

BMI data were used to characterise patients into the following weight categories: Underweight (less than 18.5), Normal weight (between 18.5 and 24.9), Overweight (between 25 and 29.9), Obese (30 or greater). Where the patient's data was not available, their weight was labelled as 'Unknown'.

**Diagnostic codes**

## Supplementary Table 1. Read codes (version 2) for diagnoses of diabetes mellitus.

| **Description** | **Code** | |
| --- | --- | --- |
| Diabetic on diet only | | 66A3. |
| Diabetic on oral treatment | | 66A4. |
| Diabetic on insulin | | 66A5. |
| Brittle diabetes | | 66AJ1 |
| Diabetic on non-insulin injectable medication | | 66o2. |
| Diabetic on oral treatment and glucagon-like peptide 1 receptor agonist | | 66o5. |
| Diabetic on insulin and glucagon-like peptide 1 receptor agonist | | 66o6. |
| Diabetes mellitus | | C10.. |
| Diabetes mellitus with no mention of complication | | C100. |
| Diabetes mellitus, juvenile type, no mention of complication | | C1000 |
| Diabetes mellitus, adult onset, no mention of complication | | C1001 |
| Diabetes mellitus NOS with no mention of complication | | C100z |
| Diabetes mellitus with ketoacidosis | | C101. |
| Diabetes mellitus, juvenile type, with ketoacidosis | | C1010 |
| Diabetes mellitus, adult onset, with ketoacidosis | | C1011 |
| Other specified diabetes mellitus with ketoacidosis | | C101y |
| Diabetes mellitus NOS with ketoacidosis | | C101z |
| Diabetes mellitus with hyperosmolar coma | | C102. |
| Diabetes mellitus, juvenile type, with hyperosmolar coma | | C1020 |
| Diabetes mellitus, adult onset, with hyperosmolar coma | | C1021 |
| Diabetes mellitus NOS with hyperosmolar coma | | C102z |
| Diabetes mellitus with ketoacidotic coma | | C103. |
| Diabetes mellitus, juvenile type, with ketoacidotic coma | | C1030 |
| Diabetes mellitus, adult onset, with ketoacidotic coma | | C1031 |
| Other specified diabetes mellitus with coma | | C103y |
| Diabetes mellitus NOS with ketoacidotic coma | | C103z |
| Diabetes mellitus with renal manifestation | | C104. |
| Diabetes mellitus, juvenile type, with renal manifestation | | C1040 |
| Diabetes mellitus, adult onset, with renal manifestation | | C1041 |
| Other specified diabetes mellitus with renal complications | | C104y |
| Diabetes mellitus with nephropathy NOS | | C104z |
| Diabetes mellitus with ophthalmic manifestation | | C105. |
| Diabetes mellitus, juvenile type, + ophthalmic manifestation | | C1050 |
| Diabetes mellitus, adult onset, + ophthalmic manifestation | | C1051 |
| Other specified diabetes mellitus with ophthalmic complicatn | | C105y |
| Diabetes mellitus NOS with ophthalmic manifestation | | C105z |
| Diabetes mellitus with neurological manifestation | | C106. |
| Diabetes mellitus, juvenile type, with neurological manifestation | | C1060 |
| Diabetes mellitus, adult onset, with neurological manifestation | | C1061 |
| Other specified diabetes mellitus with neurological complications | | C106y |
| Diabetes mellitus NOS with neurological manifestation | | C106z |
| Diabetes mellitus with peripheral circulatory disorder | | C107. |
| Diabetes mellitus, juvenile type, with peripheral circulatory disorder | | C1070 |
| Diabetes mellitus, adult onset, with peripheral circulatory disorder | | C1071 |
| Diabetes mellitus, adult with gangrene | | C1072 |
| Other specified diabetes mellitus with periph circ comps | | C107y |
| Diabetes mellitus NOS with peripheral circulatory disorder | | C107z |
| Insulin dependent diabetes mellitus | | C108. |
| Insulin-dependent diabetes mellitus with renal complications | | C1080 |
| Insulin-dependent diabetes mellitus with ophthalmic comps | | C1081 |
| Insulin-dependent diabetes mellitus with neurological comps | | C1082 |
| Insulin dependent diabetes mellitus with multiple complicatn | | C1083 |
| Unstable insulin dependent diabetes mellitus | | C1084 |
| Insulin dependent diabetes mellitus with ulcer | | C1085 |
| Insulin dependent diabetes mellitus with gangrene | | C1086 |
| Insulin dependent diabetes mellitus with retinopathy | | C1087 |
| Insulin dependent diabetes mellitus - poor control | | C1088 |
| Insulin dependent diabetes maturity onset | | C1089 |
| Insulin-dependent diabetes without complication | | C108A |
| Insulin dependent diabetes mellitus with mononeuropathy | | C108B |
| Insulin dependent diabetes mellitus with polyneuropathy | | C108C |
| Insulin dependent diabetes mellitus with nephropathy | | C108D |
| Insulin dependent diabetes mellitus with hypoglycaemic coma | | C108E |
| Insulin dependent diabetes mellitus with diabetic cataract | | C108F |
| Insulin dependent diab mell with peripheral angiopathy | | C108G |
| Insulin dependent diabetes mellitus with arthropathy | | C108H |
| Insulin dependent diab mell with neuropathic arthropathy | | C108J |
| Other specified diabetes mellitus with multiple comps | | C108y |
| Unspecified diabetes mellitus with multiple complications | | C108z |
| Non-insulin dependent diabetes mellitus | | C109. |
| Non-insulin-dependent diabetes mellitus with renal comps | | C1090 |
| Non-insulin-dependent diabetes mellitus with ophthalm comps | | C1091 |
| Non-insulin-dependent diabetes mellitus with neuro comps | | C1092 |
| Non-insulin-dependent diabetes mellitus with multiple comps | | C1093 |
| Non-insulin dependent diabetes mellitus with ulcer | | C1094 |
| Non-insulin dependent diabetes mellitus with gangrene | | C1095 |
| Non-insulin-dependent diabetes mellitus with retinopathy | | C1096 |
| Non-insulin dependent diabetes mellitus - poor control | | C1097 |
| Non-insulin-dependent diabetes mellitus without complication | | C1099 |
| Non-insulin dependent diabetes mellitus with mononeuropathy | | C109A |
| Non-insulin dependent diabetes mellitus with polyneuropathy | | C109B |
| Non-insulin dependent diabetes mellitus with nephropathy | | C109C |
| Non-insulin dependent diabetes mellitus with hypoglyca coma | | C109D |
| Non-insulin depend diabetes mellitus with diabetic cataract | | C109E |
| Non-insulin-dependent d m with peripheral angiopath | | C109F |
| Non-insulin dependent diabetes mellitus with arthropathy | | C109G |
| Non-insulin dependent d m with neuropathic arthropathy | | C109H |
| Insulin treated Type 2 diabetes mellitus | | C109J |
| Hyperosmolar non-ketotic state in type 2 diabetes mellitus | | C109K |
| Malnutrition-related diabetes mellitus | | C10A. |
| Malnutrition-related diabetes mellitus with coma | | C10A0 |
| Malnutrition-related diabetes mellitus with ketoacidosis | | C10A1 |
| Malnutrition-related diabetes mellitus with renal complicatn | | C10A2 |
| Malnutrit-related diabetes mellitus wth ophthalmic complicat | | C10A3 |
| Malnutrition-related diabetes mellitus wth neuro complicatns | | C10A4 |
| Malnutritn-relat diabetes melitus wth periph circul complctn | | C10A5 |
| Malnutrition-related diabetes mellitus with multiple comps | | C10A6 |
| Malnutrition-related diabetes mellitus without complications | | C10A7 |
| Malnutrit-related diabetes mellitus with unspec complics | | C10AW |
| Malnutrit-relat diabetes mellitus with other spec comps | | C10AX |
| Diabetes mellitus induced by steroids | | C10B. |
| Steroid induced diabetes mellitus without complication | | C10B0 |
| Diabetes mellitus autosomal dominant | | C10C. |
| Diabetes mellitus autosomal dominant type 2 | | C10D. |
| Type 1 diabetes mellitus | | C10E. |
| Type 1 diabetes mellitus with renal complications | | C10E0 |
| Type 1 diabetes mellitus with ophthalmic complications | | C10E1 |
| Type 1 diabetes mellitus with neurological complications | | C10E2 |
| Type 1 diabetes mellitus with multiple complications | | C10E3 |
| Unstable type 1 diabetes mellitus | | C10E4 |
| Type 1 diabetes mellitus with ulcer | | C10E5 |
| Type 1 diabetes mellitus with gangrene | | C10E6 |
| Type 1 diabetes mellitus with retinopathy | | C10E7 |
| Type 1 diabetes mellitus - poor control | | C10E8 |
| Type 1 diabetes mellitus maturity onset | | C10E9 |
| Type 1 diabetes mellitus without complication | | C10EA |
| Type 1 diabetes mellitus with mononeuropathy | | C10EB |
| Type 1 diabetes mellitus with polyneuropathy | | C10EC |
| Type 1 diabetes mellitus with nephropathy | | C10ED |
| Type 1 diabetes mellitus with hypoglycaemic coma | | C10EE |
| Type 1 diabetes mellitus with diabetic cataract | | C10EF |
| Type 1 diabetes mellitus with peripheral angiopathy | | C10EG |
| Type 1 diabetes mellitus with arthropathy | | C10EH |
| Type 1 diabetes mellitus with neuropathic arthropathy | | C10EJ |
| Type 1 diabetes mellitus with persistent proteinuria | | C10EK |
| Type 1 diabetes mellitus with persistent microalbuminuria | | C10EL |
| Type 1 diabetes mellitus with ketoacidosis | | C10EM |
| Type 1 diabetes mellitus with ketoacidotic coma | | C10EN |
| Type 1 diabetes mellitus with exudative maculopathy | | C10EP |
| Type 1 diabetes mellitus with gastroparesis | | C10EQ |
| Latent autoimmune diabetes mellitus in adult | | C10ER |
| Type 2 diabetes mellitus | | C10F. |
| Type 2 diabetes mellitus with renal complications | | C10F0 |
| Type 2 diabetes mellitus with ophthalmic complications | | C10F1 |
| Type 2 diabetes mellitus with neurological complications | | C10F2 |
| Type 2 diabetes mellitus with multiple complications | | C10F3 |
| Type 2 diabetes mellitus with ulcer | | C10F4 |
| Type 2 diabetes mellitus with gangrene | | C10F5 |
| Type 2 diabetes mellitus with retinopathy | | C10F6 |
| Type 2 diabetes mellitus - poor control | | C10F7 |
| Type 2 diabetes mellitus without complication | | C10F9 |
| Type 2 diabetes mellitus with mononeuropathy | | C10FA |
| Type 2 diabetes mellitus with polyneuropathy | | C10FB |
| Type 2 diabetes mellitus with nephropathy | | C10FC |
| Type 2 diabetes mellitus with hypoglycaemic coma | | C10FD |
| Type 2 diabetes mellitus with diabetic cataract | | C10FE |
| Type 2 diabetes mellitus with peripheral angiopathy | | C10FF |
| Type 2 diabetes mellitus with arthropathy | | C10FG |
| Type 2 diabetes mellitus with neuropathic arthropathy | | C10FH |
| Insulin treated Type 2 diabetes mellitus | | C10FJ |
| Hyperosmolar non-ketotic state in type 2 diabetes mellitus | | C10FK |
| Type 2 diabetes mellitus with persistent proteinuria | | C10FL |
| Type 2 diabetes mellitus with persistent microalbuminuria | | C10FM |
| Type 2 diabetes mellitus with ketoacidosis | | C10FN |
| Type 2 diabetes mellitus with ketoacidotic coma | | C10FP |
| Type 2 diabetes mellitus with exudative maculopathy | | C10FQ |
| Type 2 diabetes mellitus with gastroparesis | | C10FR |
| Maternally inherited diabetes mellitus | | C10FS |
| Diabetes mellitus induced by non-steroid drugs | | C10H. |
| DM induced by non-steroid drugs without complication | | C10H0 |
| Lipoatrophic diabetes mellitus | | C10M. |
| Lipoatrophic diabetes mellitus without complication | | C10M0 |
| Maturity onset diabetes of the young type 5 | | C10Q. |
| Diabetes mellitus with other specified manifestation | | C10y. |
| Diabetes mellitus, juvenile, + other specified manifestation | | C10y0 |
| Diabetes mellitus, adult, + other specified manifestation | | C10y1 |
| Other specified diabetes mellitus with other spec comps | | C10yy |
| Diabetes mellitus NOS with other specified manifestation | | C10yz |
| Diabetes mellitus with unspecified complication | | C10z. |
| Diabetes mellitus, juvenile type, + unspecified complication | | C10z0 |
| Diabetes mellitus, adult onset, + unspecified complication | | C10z1 |
| Other specified diabetes mellitus with unspecified comps | | C10zy |
| Diabetes mellitus NOS with unspecified complication | | C10zz |
| Steroid induced diabetes | | C11y0 |
| [X]Diabetes mellitus | | Cyu2. |
| [X]Other specified diabetes mellitus | | Cyu20 |
| [X]Malnutrition-related diabetes mellitus with other specified complications | | Cyu21 |
| [X]Malnutrition-related diabetes mellitus with unspecified complications | | Cyu22 |
| [X]Unspecified diabetes mellitus with renal complications | | Cyu23 |
| Diabetes mellitus during pregnancy, childbirth and the puerperium | | L180. |
| Diabetes mellitus – unspecified whether during pregnancy or the puerperium | | L1800 |
| Diabetes mellitus during pregnancy – baby delivered | | L1801 |
| Diabetes mellitus during pregnancy – baby delivered during current episode of care | | L1802 |
| Diabetes mellitus during pregnancy – baby not yet delivered | | L1803 |
| Diabetes mellitus during pregnancy – baby delivered during previous episode of care | | L1804 |
| Pre-existing diabetes mellitus, insulin-dependent | | L1805 |
| Pre-existing diabetes mellitus, non-insulin-dependent | | L1806 |
| Pre-existing malnutrition-related diabetes mellitus | | L1807 |
| Diabetes mellitus arising in pregnancy | | L1808 |
| Pre-existing type 1 diabetes mellitus in pregnancy | | L180A |
| Pre-existing type 2 diabetes mellitus in pregnancy | | L180B |
| Pre-existing diabetes mellitus, unspecified | | L180X |
| Diabetes mellitus during pregnancy, childbirth and the puerperium NOS | | L180z |
| [X]Pre-existing diabetes mellitus, unspecified | | Lyu29 |
| Neonatal diabetes mellitus | | Q441. |

## Supplementary Table 2a. Read codes (version 2) for diagnoses of ischaemic heart disease.

| **Description** | **Code** |
| --- | --- |
| H/O: myocardial infarct <60 | 14A3. |
| H/O: myocardial infarct >60 | 14A4. |
| H/O: angina pectoris | 14A5. |
| H/O: heart disease NOS | 14AA. |
| H/O: Myocardial infarction in last year | 14AH. |
| H/O: Angina in last year | 14AJ. |
| H/O: Treatment for ischaemic heart disease | 14AL. |
| History of myocardial infarction | 14AT. |
| Frequency of angina | 187.. |
| Angina control | 662K. |
| Angina control - good | 662K0 |
| Angina control - poor | 662K1 |
| Angina control - improving | 662K2 |
| Angina control - worsening | 662K3 |
| Angina self management plan commenced | 662K4 |
| Angina self management plan completed | 662K5 |
| Angina control NOS | 662Kz |
| CHD monitoring | 662N. |
| Antianginal therapy | 8B27. |
| Ischaemic heart disease | G3... |
| Acute myocardial infarction | G30.. |
| Acute anterolateral infarction | G300. |
| Other specified anterior myocardial infarction | G301. |
| Acute anteroapical infarction | G3010 |
| Acute anteroseptal infarction | G3011 |
| Anterior myocardial infarction NOS | G301z |
| Acute inferolateral infarction | G302. |
| Acute inferoposterior infarction | G303. |
| Posterior myocardial infarction NOS | G304. |
| Lateral myocardial infarction NOS | G305. |
| True posterior myocardial infarction | G306. |
| Acute subendocardial infarction | G307. |
| Acute non-Q wave infarction | G3070 |
| Acute non-ST segment elevation myocardial infarction | G3071 |
| Inferior myocardial infarction NOS | G308. |
| Acute Q-wave infarct | G309. |
| Mural thrombosis | G30A. |
| Acute posterolateral myocardial infarction | G30B. |
| Acute transmural myocardial infarction of unspecified site | G30X. |
| Acute ST segment elevation myocardial infarction | G30X0 |
| Other acute myocardial infarction | G30y. |
| Acute atrial infarction | G30y0 |
| Acute papillary muscle infarction | G30y1 |
| Acute septal infarction | G30y2 |
| Other acute myocardial infarction NOS | G30yz |
| Acute myocardial infarction NOS | G30z. |
| Other acute and subacute ischaemic heart disease | G31.. |
| Postmyocardial infarction syndrome | G310. |
| Preinfarction syndrome | G311. |
| Myocardial infarction aborted | G3110 |
| Unstable angina | G3111 |
| Angina at rest | G3112 |
| Refractory angina | G3113 |
| Worsening angina | G3114 |
| Acute coronary syndrome | G3115 |
| Preinfarction syndrome NOS | G311z |
| Coronary thrombosis not resulting in myocardial infarction | G312. |
| Other acute and subacute ischaemic heart disease | G31y. |
| Acute coronary insufficiency | G31y0 |
| Microinfarction of heart | G31y1 |
| Subendocardial ischaemia | G31y2 |
| Transient myocardial ischaemia | G31y3 |
| Other acute and subacute ischaemic heart disease NOS | G31yz |
| Old myocardial infarction | G32.. |
| Angina pectoris | G33.. |
| Angina decubitus | G330. |
| Nocturnal angina | G3300 |
| Angina decubitus NOS | G330z |
| Coronary artery spasm | G332. |
| Angina pectoris NOS | G33z. |
| Status anginosus | G33z0 |
| Stenocardia | G33z1 |
| Syncope anginosa | G33z2 |
| Angina on effort | G33z3 |
| Ischaemic chest pain | G33z4 |
| Post infarct angina | G33z5 |
| New onset angina | G33z6 |
| Stable angina | G33z7 |
| Angina pectoris NOS | G33zz |
| Other chronic ischaemic heart disease | G34.. |
| Coronary atherosclerosis | G340. |
| Single coronary vessel disease | G3400 |
| Double coronary vessel disease | G3401 |
| Acquired atrioventricular fistula of heart | G3413 |
| Atherosclerotic cardiovascular disease | G342. |
| Ischaemic cardiomyopathy | G343. |
| Silent myocardial ischaemia | G344. |
| Other specified chronic ischaemic heart disease | G34y. |
| Chronic coronary insufficiency | G34y0 |
| Chronic myocardial ischaemia | G34y1 |
| Other specified chronic ischaemic heart disease NOS | G34yz |
| Other chronic ischaemic heart disease NOS | G34z. |
| Asymptomatic coronary heart disease | G34z0 |
| Subsequent myocardial infarction | G35.. |
| Subsequent myocardial infarction of anterior wall | G350. |
| Subsequent myocardial infarction of inferior wall | G351. |
| Subsequent myocardial infarction of other sites | G353. |
| Subsequent myocardial infarction of unspecified site | G35X. |
| Certain current complications following acute myocardial infarction | G36.. |
| Haemopericardium as current complication following acute myocardial infarction | G360. |
| Atrial septal defect as current complication following acute myocardial infarction | G361. |
| Ventricular septal defect as current complication following acute myocardial infarction | G362. |
| Rupture of cardiac wall without haemopericardium as current complication following acute myocardial infarction | G363. |
| Rupture of chordae tendinae as current complication following acute myocardial infarction | G364. |
| Rupture of papillary muscle as current complication following acute myocardial infarction | G365. |
| Thrombosis of atrium, auricular appendage, and ventricle as current complications following acute myocardial infarction | G366. |
| Postoperative myocardial infarction | G38.. |
| Postoperative transmural myocardial infarction of anterior wall | G380. |
| Postoperative transmural myocardial infarction of inferior wall | G381. |
| Postoperative transmural myocardial infarction of other sites | G382. |
| Postoperative transmural myocardial infarction of unspecified site | G383. |
| Postoperative subendocardial myocardial infarction | G384. |
| Postoperative myocardial infarction, unspecified | G38z. |
| Other specified ischaemic heart disease | G3y.. |
| Ischaemic heart disease NOS | G3z.. |
| Non-obstructive coronary atherosclerosis | G704. |

## Supplementary Table 2b. Read codes (version 2) for diagnoses of peripheral arterial disease.

| **Description** | **Code** |
| --- | --- |
| H/O: Peripheral vascular disease procedure | 14NB. |
| Peripheral vascular disease monitoring | 662U. |
| Operations on aortic root | 791C. |
| Aortic root replacement using pulmonary valve autograft with right ventricle to pulmonary artery valved conduit | 791C0 |
| Aortic root replacement using pulmonary valve autograft with right ventricle to pulmonary artery valved conduit and aortoventriculoplasty | 791C1 |
| Aortic root replacement using homograft | 791C2 |
| Aortic root replacement using mechanical prosthesis | 791C3 |
| Aortic root replacement | 791C4 |
| Other specified operations on aortic root | 791Cy |
| Operations on aortic root NOS | 791Cz |
| Extraanatomic bypass of aorta | 7A10. |
| Emergency bypass of aorta by anastomosis of axillary artery to femoral artery | 7A100 |
| Bypass of aorta by anastomosis of axillary artery to femoral artery NEC | 7A101 |
| Axillo-bifemoral bypass graft | 7A102 |
| Axillo-unifemoral PTFE bypass graft | 7A103 |
| Bypass of aorta by anastomosis of axillary artery to bilateral femoral arteries | 7A104 |
| Other specified extraanatomic bypass of aorta | 7A10y |
| Extraanatomic bypass of aorta NOS | 7A10z |
| Replacement of aneurysmal bifurcation of aorta | 7A11. |
| Emergency replacement of aneurysmal bifurcation of aorta by anastomosis of aorta to femoral artery | 7A110 |
| Replacement of aneurysmal bifurcation of aorta by anastomosis of aorta to femoral artery | 7A111 |
| Emergency replacement of aneurysmal bifurcation of aorta by anastomosis of aorta to iliac artery | 7A112 |
| Replacement of aneurysmal bifurcation of aorta by anastomosis of aorta to iliac artery | 7A113 |
| Other specified replacement of aneurysmal bifurcation of aorta | 7A11y |
| Replacement of aneurysmal bifurcation of aorta NOS | 7A11z |
| Other bypass of bifurcation of aorta | 7A12. |
| Emergency bypass of bifurcation of aorta by anastomosis of aorta to femoral artery | 7A120 |
| Bypass of bifurcation of aorta by anastomosis of aorta to femoral artery NEC | 7A121 |
| Emergency bypass of bifurcation of aorta by anastomosis of aorta to iliac artery | 7A122 |
| Bypass of bifurcation of aorta by anastomosis of aorta to iliac artery | 7A123 |
| Other specified other bypass of bifurcation of aorta | 7A12y |
| Other bypass of bifurcation of aorta NOS | 7A12z |
| Emergency replacement of aneurysmal segment of aorta | 7A13. |
| Emergency replacement of aneurysmal segment of ascending aorta by anastomosis of aorta to aorta | 7A130 |
| Emergency replacement of aneurysmal segment of thoracic aorta by anastomosis of aorta to aorta | 7A131 |
| Emergency replacement of aneurysmal segment of suprarenal abdominal aorta by anastomosis of aorta to aorta | 7A132 |
| Emergency replacement of aneurysmal segment of infrarenal abdominal aorta by anastomosis of aorta to aorta | 7A133 |
| Emergency replacement of aneurysmal segment of abdominal aorta by anastomosis of aorta to aorta NEC | 7A134 |
| Other specified emergency replacement of aneurysmal segment of aorta | 7A13y |
| Emergency replacement of aneurysmal segment of aorta NOS | 7A13z |
| Other replacement of aneurysmal segment of aorta | 7A14. |
| Replacement of aneurysmal segment of ascending aorta by anastomosis of aorta to aorta NEC | 7A140 |
| Replacement of aneurysmal segment of thoracic aorta by anastomosis of aorta to aorta NEC | 7A141 |
| Replacement of aneurysmal segment of suprarenal abdominal aorta by anastomosis of aorta to aorta NEC | 7A142 |
| Replacement of aneurysmal segment of infrarenal abdominal aorta by anastomosis of aorta to aorta NEC | 7A143 |
| Replacement of aneurysmal segment of abdominal aorta by anastomosis of aorta to aorta NEC | 7A144 |
| Other specified other replacement of aneurysmal segment of aorta | 7A14y |
| Other replacement of aneurysmal segment of aorta NOS | 7A14z |
| Transluminal operations on aneurysmal segment of aorta | 7A1B. |
| Endovascular stenting of infrarenal abdominal aortic aneurysm | 7A1B0 |
| Endovascular stenting of suprarenal aortic aneurysm | 7A1B1 |
| Endovascular stenting of thoracic aortic aneurysm | 7A1B2 |
| Endovascular stenting of aortic dissection in any position | 7A1B3 |
| Endovascular stenting of aortic bifurcation NEC | 7A1B4 |
| Endovascular stenting of aorto-uniiliac aneurysm | 7A1B5 |
| Endovascular stenting for aortic aneurysm of bifurcation NEC | 7A1B6 |
| Endovascular stenting for aorto-uniiliac aneurysm | 7A1B7 |
| Endovascular insertion of stent for infrarenal abdominal aortic aneurysm | 7A1B8 |
| Endovascular insertion of stent for suprarenal aortic aneurysm | 7A1B9 |
| Endovascular insertion of stent for thoracic aortic aneurysm | 7A1BA |
| Endovascular insertion of stent for aortic dissection in any position | 7A1BB |
| Endovascular insertion of stent for aortic aneurysm of bifurcation NEC | 7A1BC |
| Endovascular insertion of stent for aorto-uniiliac aneurysm | 7A1BD |
| Other specified transluminal operations on aneurysmal segment of aorta | 7A1By |
| Transluminal operations on aneurysmal segment of aorta NOS | 7A1Bz |
| Transluminal insertion of stent graft for aneurysmal segment of aorta | 7A1C. |
| Endovascular insertion of stent graft for infrarenal abdominal aortic aneurysm | 7A1C0 |
| Endovascular insertion of stent graft for suprarenal aortic aneurysm | 7A1C1 |
| Endovascular insertion of stent graft for thoracic aortic aneurysm | 7A1C2 |
| Endovascular insertion of stent graft for aortic dissection in any position | 7A1C3 |
| Endovascular insertion of stent graft for aortic bifurcation NEC | 7A1C4 |
| Endovascular insertion of stent graft for aorto-uniiliac aneurysm | 7A1C5 |
| Other specified transluminal insertion of stent graft for aneurysmal segment of aorta | 7A1Cy |
| Transluminal insertion of stent graft for aneurysmal segment of aorta NOS | 7A1Cz |
| Percutaneous transluminal angioplasty of carotid artery | 7A220 |
| Endovascular repair of carotid artery | 7A222 |
| Percutaneous transluminal insertion of stent into carotid artery | 7A223 |
| Other specified transluminal operation on carotid artery | 7A22y |
| Transluminal operation on carotid artery NOS | 7A22z |
| Other open operations on subclavian artery | 7A27. |
| Operation on aneurysm of subclavian artery | 7A27C |
| Operation on aneurysm of axillary artery | 7A27D |
| Operation on aneurysm of brachial artery | 7A27E |
| Operation on aneurysm of vertebral artery | 7A27F |
| Percutaneous transluminal angioplasty of subclavian artery | 7A280 |
| Percutaneous transluminal angioplasty of brachial artery | 7A281 |
| Percutaneous transluminal angioplasty of vertebral artery | 7A282 |
| Percutaneous transluminal angioplasty of axillary artery | 7A28C |
| Percutaneous transluminal insertion of stent into subclavian artery | 7A28G |
| Operation on aneurysm of coeliac artery NEC | 7A34C |
| Operation on aneurysm of superior mesenteric artery NEC | 7A34D |
| Operation on aneurysm of inferior mesenteric artery NEC | 7A34E |
| Operation on aneurysm of suprarenal artery NEC | 7A34F |
| Operation on aneurysm of visceral branch of abdominal aorta NEC | 7A34K |
| Percutaneous transluminal angioplasty of coeliac artery NEC | 7A350 |
| Percutaneous transluminal angioplasty of superior mesenteric artery NEC | 7A351 |
| Percutaneous transluminal angioplasty of inferior mesenteric artery NEC | 7A352 |
| Percutaneous transluminal angioplasty of suprarenal artery NEC | 7A353 |
| Percutaneous transluminal insertion of stent into visceral branch of abdominal aorta NEC | 7A35D |
| Percutaneous transluminal insertion of stent into superior mesenteric artery | 7A35E |
| Replacement of aneurysmal iliac artery | 7A40. |
| Emergency replacement of aneurysmal iliac artery by anastomosis of iliac artery to femoral artery | 7A400 |
| Replacement of aneurysmal iliac artery by anastomosis of iliac artery to femoral artery NEC | 7A401 |
| Emergency replacement of aneurysmal iliac artery by anastomosis of femoral artery to femoral artery | 7A402 |
| Replacement of aneurysmal iliac artery by anastomosis of femoral artery to femoral artery NEC | 7A403 |
| Emergency replacement of aneurysmal common iliac artery by anastomosis of aorta to common iliac artery | 7A404 |
| Emergency replacement of aneurysmal iliac artery by anastomosis of aorta to external iliac artery | 7A405 |
| Emergency replacement of aneurysmal artery of leg by anastomosis of aorta to common femoral artery | 7A406 |
| Emergency replacement of aneurysmal artery of leg by anastomosis of aorta to superficial femoral artery | 7A407 |
| Emergency replacement of aneurysmal iliac artery by anastomosis of iliac artery to iliac artery | 7A408 |
| Replacement of aneurysmal common iliac artery by anastomosis of aorta to common iliac artery NEC | 7A409 |
| Replacement of aneurysmal iliac artery by anastomosis of aorta to external iliac artery NEC | 7A40A |
| Replacement of aneurysmal artery of leg by anastomosis of aorta to common femoral artery NEC | 7A40B |
| Replacement of aneurysmal artery of leg by anastomosis of aorta to superficial femoral artery NEC | 7A40C |
| Replacement of aneurysmal iliac artery by anastomosis of iliac artery to iliac artery NEC | 7A40D |
| Other specified replacement of aneurysmal iliac artery | 7A40y |
| Replacement of aneurysmal iliac artery NOS | 7A40z |
| Other bypass of iliac artery | 7A41. |
| Emergency bypass of iliac artery by anastomosis of iliac artery to femoral artery NEC | 7A410 |
| Bypass of iliac artery by anastomosis of iliac artery to femoral artery NEC | 7A411 |
| Emergency bypass of iliac artery by anastomosis of femoral artery to femoral artery NEC | 7A412 |
| Bypass of iliac artery by anastomosis of femoral artery to femoral artery NEC | 7A413 |
| Emergency bypass of common iliac artery by anastomosis of aorta to common iliac artery NEC | 7A414 |
| Emergency bypass of iliac artery by anastomosis of aorta to external iliac artery NEC | 7A415 |
| Emergency bypass of artery of leg by anastomosis of aorta to common femoral artery NEC | 7A416 |
| Emergency bypass of artery of leg by anastomosis of aorta to deep femoral artery NEC | 7A417 |
| Emergency bypass of iliac artery by anastomosis of iliac artery to iliac artery NEC | 7A418 |
| Bypass of common iliac artery by anastomosis of aorta to common iliac artery NEC | 7A419 |
| Bypass of iliac artery by anastomosis of aorta to external iliac NEC | 7A41A |
| Bypass of artery of leg by anastomosis of aorta to common femoral artery NEC | 7A41B |
| Bypass of artery of leg by anastomosis of aorta to deep femoral artery NEC | 7A41C |
| Bypass of iliac artery by anastomosis of iliac artery to iliac artery NEC | 7A41D |
| Emergency bypass of iliac artery by unspecified anastomosis | 7A41E |
| Ilio-femoral prosthetic cross over graft | 7A41F |
| Other specified other bypass of iliac artery | 7A41y |
| Other bypass of iliac artery NOS | 7A41z |
| Reconstruction of iliac artery | 7A42. |
| Endarterectomy of iliac artery and patch repair of iliac artery | 7A420 |
| Endarterectomy of iliac artery NEC | 7A421 |
| Other specified reconstruction of iliac artery | 7A42y |
| Reconstruction of iliac artery NOS | 7A42z |
| Operation on aneurysm of iliac artery NEC | 7A432 |
| Open insertion of iliac artery stent | 7A433 |
| Percutaneous transluminal angioplasty of iliac artery | 7A440 |
| Insertion of iliac artery stent | 7A443 |
| Percutaneous transluminal insertion of iliac artery stent | 7A444 |
| Emergency replacement of aneurysmal femoral or popliteal artery | 7A45. |
| Emergency replacement of aneurysmal femoral artery by anastomosis of femoral artery to popliteal artery using prosthesis | 7A450 |
| Emergency replacement of aneurysmal popliteal artery by anastomosis of popliteal artery to popliteal artery using prosthesis | 7A451 |
| Emergency replacement of aneurysmal femoral artery by anastomosis of femoral artery to popliteal artery using vein graft | 7A452 |
| Emergency replacement of aneurysmal popliteal artery by anastomosis of popliteal artery to popliteal artery using vein graft | 7A453 |
| Emergency replacement of aneurysmal femoral artery by anastomosis of femoral artery to tibial artery using prosthesis | 7A454 |
| Emergency replacement of aneurysmal popliteal artery by anastomosis of popliteal artery to tibial artery using prosthesis | 7A455 |
| Emergency replacement of aneurysmal femoral artery by anastomosis of femoral artery to tibial artery using vein graft | 7A456 |
| Emergency replacement of aneurysmal popliteal artery by anastomosis of popliteal artery to tibial artery using vein graft | 7A457 |
| Emergency replacement of aneurysmal femoral artery by anastomosis of femoral artery to peroneal artery using prosthesis | 7A458 |
| Emergency replacement of aneurysmal popliteal artery by anastomosis of popliteal artery to peroneal artery using prosthesis | 7A459 |
| Emergency replacement of aneurysmal femoral artery by anastomosis of femoral artery to peroneal artery using vein graft | 7A45A |
| Emergency replacement of aneurysmal popliteal artery by anastomosis of popliteal artery to peroneal artery using vein graft | 7A45B |
| Emergency replacement of aneurysmal femoral artery by anastomosis of femoral artery to femoral artery | 7A45C |
| Emergency replacement of aneurysmal popliteal artery by anastomosis of popliteal artery to femoral artery | 7A45D |
| Other specified emergency replacement of aneurysmal femoral artery or popliteal artery | 7A45y |
| Emergency replacement of aneurysmal femoral artery or popliteal artery NOS | 7A45z |
| Other replacement of aneurysmal femoral artery | 7A46. |
| Replacement of aneurysmal femoral artery by anastomosis of femoral artery to popliteal artery using prosthesis NEC | 7A460 |
| Replacement of aneurysmal popliteal artery by anastomosis of popliteal artery to popliteal artery using prosthesis NEC | 7A461 |
| Replacement of aneurysmal femoral artery by anastomosis of femoral artery to popliteal artery using vein graft NEC | 7A462 |
| Replacement of aneurysmal popliteal artery by anastomosis of popliteal artery to popliteal artery using vein graft NEC | 7A463 |
| Replacement of aneurysmal femoral artery by anastomosis of femoral artery to tibial artery using prosthesis NEC | 7A464 |
| Replacement of aneurysmal popliteal artery by anastomosis of popliteal artery to tibial artery using prosthesis NEC | 7A465 |
| Replacement of aneurysmal femoral artery by anastomosis of femoral artery to tibial artery using vein graft NEC | 7A466 |
| Replacement of aneurysmal popliteal artery by anastomosis of popliteal artery to tibial artery using vein graft NEC | 7A467 |
| Replacement of aneurysmal femoral artery by anastomosis of femoral artery to peroneal artery using prosthesis NEC | 7A468 |
| Replacement of aneurysmal popliteal artery by anastomosis of popliteal artery to peroneal artery using prosthesis NEC | 7A469 |
| Replacement of aneurysmal femoral artery by anastomosis of femoral artery to peroneal artery using vein graft NEC | 7A46A |
| Replacement of aneurysmal popliteal artery by anastomosis of popliteal artery to peroneal artery using vein graft NEC | 7A46B |
| Replacement of aneurysmal femoral artery by anastomosis of femoral artery to femoral artery NEC | 7A46C |
| Replacement of aneurysmal popliteal artery by anastomosis of popliteal artery to femoral artery NEC | 7A46D |
| Other specified other replacement of aneurysmal femoral artery or popliteal artery | 7A46y |
| Other replacement of aneurysmal femoral artery or popliteal artery NOS | 7A46z |
| Other emergency bypass of femoral artery or popliteal artery | 7A47. |
| Emergency bypass of femoral artery by anastomosis of femoral artery to popliteal artery using prosthesis NEC | 7A470 |
| Emergency bypass of popliteal artery by anastomosis of popliteal artery to popliteal artery using prosthesis NEC | 7A471 |
| Emergency bypass of femoral artery by anastomosis of femoral artery to popliteal artery using vein graft NEC | 7A472 |
| Emergency bypass of popliteal artery by anastomosis of popliteal artery to popliteal artery using vein graft NEC | 7A473 |
| Emergency bypass of femoral artery by anastomosis of femoral artery to tibial artery using prosthesis NEC | 7A474 |
| Emergency bypass of popliteal artery by anastomosis of popliteal artery to tibial artery using prosthesis NEC | 7A475 |
| Emergency bypass of femoral artery by anastomosis of femoral artery to tibial artery using vein graft NEC | 7A476 |
| Emergency bypass of popliteal artery by anastomosis of popliteal artery to tibial artery using vein graft NEC | 7A477 |
| Emergency bypass of femoral artery by anastomosis of femoral artery to peroneal artery using prosthesis NEC | 7A478 |
| Emergency bypass of popliteal artery by anastomosis of popliteal artery to peroneal artery using prosthesis NEC | 7A479 |
| Emergency bypass of femoral artery by anastomosis of femoral artery to peroneal artery using vein graft NEC | 7A47A |
| Emergency bypass of popliteal artery by anastomosis of popliteal artery to peroneal artery using vein graft NEC | 7A47B |
| Emergency bypass of femoral artery by anastomosis of femoral artery to femoral artery NEC | 7A47C |
| Emergency bypass of popliteal artery by anastomosis of popliteal artery to femoral artery NEC | 7A47D |
| Other specified other emergency bypass of femoral artery or popliteal artery | 7A47y |
| Other emergency bypass of femoral artery or popliteal artery NOS | 7A47z |
| Other bypass of femoral artery or popliteal artery | 7A48. |
| Bypass of femoral artery by anastomosis of femoral artery to popliteal artery using prosthesis NEC | 7A480 |
| Bypass of popliteal artery by anastomosis of popliteal artery to popliteal artery using prosthesis NEC | 7A481 |
| Bypass of femoral artery by anastomosis of femoral artery to popliteal artery using vein graft NEC | 7A482 |
| Bypass of popliteal artery by anastomosis of popliteal artery to popliteal artery using vein graft NEC | 7A483 |
| Bypass of femoral artery by anastomosis of femoral artery to tibial artery using prosthesis NEC | 7A484 |
| Bypass of popliteal artery by anastomosis of popliteal artery to tibial artery using prosthesis NEC | 7A485 |
| Bypass of femoral artery by anastomosis of femoral artery to tibial artery using vein graft NEC | 7A486 |
| Bypass of popliteal artery by anastomosis of popliteal artery to tibial artery using vein graft NEC | 7A487 |
| Bypass of femoral artery by anastomosis of femoral artery to peroneal artery using prosthesis NEC | 7A488 |
| Bypass of popliteal artery by anastomosis of popliteal artery to peroneal artery using prosthesis NEC | 7A489 |
| Bypass of femoral artery by anastomosis of femoral artery to peroneal artery using vein graft NEC | 7A48A |
| Bypass of popliteal artery by anastomosis of popliteal artery to peroneal artery using vein graft NEC | 7A48B |
| Bypass of femoral artery by anastomosis of femoral artery to femoral artery NEC | 7A48C |
| Bypass of popliteal artery by anastomosis of popliteal artery to femoral artery NEC | 7A48D |
| Femoro-femoral prosthetic cross over graft | 7A48E |
| Other specified other bypass of femoral artery or popliteal artery | 7A48y |
| Other bypass of femoral artery or popliteal artery NOS | 7A48z |
| Reconstruction of femoral artery or popliteal artery | 7A49. |
| Endarterectomy of femoral artery and patch repair of femoral artery | 7A490 |
| Endarterectomy of popliteal artery and patch repair of popliteal artery | 7A491 |
| Endarterectomy of femoral artery NEC | 7A492 |
| Endarterectomy of popliteal artery NEC | 7A493 |
| Profundoplasty of femoral artery and patch repair of deep femoral artery | 7A494 |
| Profundoplasty of popliteal artery and patch repair of popliteal artery | 7A495 |
| Profundoplasty of femoral artery NEC | 7A496 |
| Profundoplasty of popliteal artery NEC | 7A497 |
| Reconstruction of femoral artery with vein graft | 7A498 |
| Reconstruction of popliteal artery with vein graft | 7A499 |
| Other specified reconstruction of femoral artery or popliteal artery | 7A49y |
| Reconstruction of femoral or popliteal artery NOS | 7A49z |
| Ligation of aneurysm of popliteal artery | 7A4A4 |
| Operation on aneurysm of femoral artery NEC | 7A4A5 |
| Percutaneous transluminal angioplasty of femoral artery | 7A4B0 |
| Percutaneous transluminal angioplasty of popliteal artery | 7A4B1 |
| Percutaneous transluminal thrombolysis of femoral graft using streptokinase | 7A4B8 |
| Percutaneous transluminal insertion of stent into femoral artery | 7A4B9 |
| Percutaneous transluminal angioplasty of artery NEC | 7A540 |
| Percutaneous transluminal dilation of artery NEC | 7A544 |
| Rotary blade angioplasty | 7A545 |
| Percutaneous transluminal atherectomy | 7A548 |
| Percutaneous transluminal stent reconstruction of artery | 7A561 |
| Percutaneous transluminal balloon angioplasty of artery | 7A564 |
| Percutaneous transluminal placement of peripheral stent in artery | 7A566 |
| Transluminal coil embolisation of aneurysm | 7A58. |
| Percutaneous transluminal coil embolisation of aneurysm small | 7A580 |
| Percutaneous transluminal coil embolisation of aneurysm medium | 7A581 |
| Percutaneous transluminal coil embolisation of aneurysm large | 7A582 |
| Percutaneous transluminal coil embolisation of aneurysm giant | 7A583 |
| Other specified transluminal coil embolisation of aneurysm | 7A58y |
| Transluminal coil embolisation of aneurysm NOS | 7A58z |
| Transluminal balloon assisted coil embolisation of aneurysm | 7A59. |
| Percutaneous transluminal balloon assisted coil embolisation of three or more aneurysms | 7A590 |
| Percutaneous transluminal balloon assisted coil embolisation of two aneurysms | 7A591 |
| Percutaneous transluminal balloon assisted coil embolisation of single aneurysm | 7A592 |
| Other specified transluminal balloon assisted coil embolisation of aneurysm | 7A59y |
| Transluminal balloon assisted coil embolisation of aneurysm NOS | 7A59z |
| Transluminal stent assisted coil embolisation of aneurysm | 7A5A. |
| Percutaneous transluminal stent assisted coil embolisation of three or more aneurysms | 7A5A0 |
| Percutaneous transluminal stent assisted coil embolisation of two aneurysms | 7A5A1 |
| Percutaneous transluminal stent assisted coil embolisation of single aneurysm | 7A5A2 |
| Other specified transluminal stent assisted coil embolisation of aneurysm | 7A5Ay |
| Transluminal stent assisted coil embolisation of aneurysm NOS | 7A5Az |
| Other transluminal embolisation of aneurysm | 7A5B. |
| Percutaneous transluminal liquid polymer embolisation of aneurysm | 7A5B0 |
| Percutaneous transluminal stent assisted liquid polymer embolisation of aneurysm | 7A5B1 |
| Other specified other transluminal embolisation of aneurysm | 7A5By |
| Other transluminal embolisation of aneurysm NOS | 7A5Bz |
| Non-insulin-dependent diabetes mellitus with peripheral angiopathy | C109F |
| Type 1 diabetes mellitus with peripheral angiopathy | C10EG |
| Type 2 diabetes mellitus with peripheral angiopathy | C10FF |
| Subclavian steal syndrome | G652. |
| Aortic atherosclerosis | G700. |
| Renal artery atherosclerosis | G701. |
| Atherosclerotic renal artery stenosis | G7010 |
| Extremity artery atheroma | G702. |
| Extremity artery atheroma NOS | G702z |
| Acquired renal artery stenosis | G703. |
| Other specified artery atheroma | G70y. |
| Arteriosclerotic vascular disease NOS | G70z. |
| Aortic aneurysm | G71.. |
| Dissecting aortic aneurysm | G710. |
| Thoracic aortic aneurysm which has ruptured | G711. |
| Thoracic aortic aneurysm without mention of rupture | G712. |
| Abdominal aortic aneurysm which has ruptured | G713. |
| Ruptured suprarenal aortic aneurysm | G7130 |
| Abdominal aortic aneurysm without mention of rupture | G714. |
| Juxtarenal aortic aneurysm | G7140 |
| Inflammatory abdominal aortic aneurysm | G7141 |
| Infrarenal abdominal aortic aneurysm | G7142 |
| Aneurysm of suprarenal aorta | G7143 |
| Ruptured aortic aneurysm NOS | G715. |
| Thoracoabdominal aortic aneurysm, ruptured | G7150 |
| Aortic aneurysm without mention of rupture NOS | G716. |
| Thoracoabdominal aortic aneurysm, without mention of rupture | G7160 |
| Leaking abdominal aortic aneurysm | G718. |
| Abscess of aortic root | G719. |
| Aortic root dilatation | G71A. |
| Aortic aneurysm NOS | G71z. |
| Other aneurysm | G72.. |
| Aneurysm of artery of arm | G720. |
| Aneurysm of brachial artery | G7200 |
| Aneurysm of radial artery | G7201 |
| Aneurysm of ulnar artery | G7202 |
| Aneurysm of arm artery NOS | G720z |
| Aneurysm of renal artery | G721. |
| Acquired renal artery aneurysm | G7210 |
| Congenital renal artery aneurysm | G7211 |
| Aneurysm of iliac artery | G722. |
| Aneurysm of common iliac artery | G7220 |
| Aneurysm of external iliac artery | G7221 |
| Aneurysm of internal iliac artery | G7222 |
| Aneurysm of iliac artery NOS | G722z |
| Aneurysm of leg artery | G723. |
| Aneurysm of femoral artery | G7230 |
| Aneurysm of popliteal artery | G7231 |
| Aneurysm of anterior tibial artery | G7232 |
| Aneurysm of dorsalis pedis artery | G7233 |
| Aneurysm of posterior tibial artery | G7234 |
| Ruptured popliteal artery aneurysm | G7235 |
| Post radiological femoral false aneurysm | G7236 |
| Aneurysm of leg artery NOS | G723z |
| Arterial false aneurysm | G724. |
| Dissection of artery of upper extremity | G725. |
| Dissection of renal artery | G726. |
| Dissection of iliac artery | G727. |
| Dissection of artery of lower extremity | G728. |
| Aneurysm and dissection of precerebral artery | G729. |
| Dissection of other specified arteries | G72A. |
| Dissection of artery | G72B. |
| Aneurysm of other artery | G72y. |
| Aneurysm of subclavian artery | G72y4 |
| Aneurysm of splenic artery | G72y5 |
| Aneurysm of axillary artery | G72y6 |
| Aneurysm of coeliac artery | G72y7 |
| Aneurysm of superior mesenteric artery | G72y8 |
| Aneurysm of inferior mesenteric artery | G72y9 |
| Aneurysm of hepatic artery | G72yA |
| Aneurysm of other visceral artery | G72yB |
| Other aneurysm NOS | G72yz |
| Aneurysm NOS | G72z. |
| Other peripheral vascular disease | G73.. |
| Peripheral arterial disease | G734. |
| Other specified peripheral vascular disease | G73y. |
| Peripheral vascular disease NOS | G73z. |
| Intermittent claudication | G73z0 |
| Peripheral vascular disease NOS | G73zz |
| Iliac artery occlusion | G76z0 |
| Femoral artery occlusion | G76z1 |
| Popliteal artery occlusion | G76z2 |
| [X]Other specified peripheral vascular diseases | Gyu74 |
|  |  |

## Supplementary Table 2c. Read codes (version 2) for diagnoses of stroke.

| **Description** | **Code** |
| --- | --- |
| H/O: CVA/stroke | 14A7. |
| Precerebral arterial occlusion | G63.. |
| Basilar artery occlusion | G630. |
| Carotid artery occlusion | G631. |
| Vertebral artery occlusion | G632. |
| Multiple and bilateral precerebral arterial occlusion | G633. |
| Carotid artery stenosis | G634. |
| Other precerebral artery occlusion | G63y. |
| Cerebral infarct due to thrombosis of precerebral arteries | G63y0 |
| Cerebral infarction due to embolism of precerebral arteries | G63y1 |
| Precerebral artery occlusion NOS | G63z. |
| Cerebral arterial occlusion | G64.. |
| Cerebral thrombosis | G640. |
| Cerebral infarction due to thrombosis of cerebral arteries | G6400 |
| Cerebral embolism | G641. |
| Cerebral infarction due to embolism of cerebral arteries | G6410 |
| Cerebral infarction NOS | G64z. |
| Brainstem infarction | G64z0 |
| Wallenberg syndrome | G64z1 |
| Left sided cerebral infarction | G64z2 |
| Right sided cerebral infarction | G64z3 |
| Infarction of basal ganglia | G64z4 |
| Transient cerebral ischaemia | G65.. |
| Basilar artery syndrome | G650. |
| Vertebral artery syndrome | G651. |
| Vertebro-basilar artery syndrome | G6510 |
| Carotid artery syndrome hemispheric | G653. |
| Multiple and bilateral precerebral artery syndromes | G654. |
| Vertebrobasilar insufficiency | G656. |
| Carotid territory transient ischaemic attack | G657. |
| Other transient cerebral ischaemia | G65y. |
| Transient cerebral ischaemia NOS | G65z. |
| Impending cerebral ischaemia | G65z0 |
| Intermittent cerebral ischaemia | G65z1 |
| Transient cerebral ischaemia NOS | G65zz |
| Stroke and cerebrovascular accident unspecified | G66.. |
| Middle cerebral artery syndrome | G660. |
| Anterior cerebral artery syndrome | G661. |
| Posterior cerebral artery syndrome | G662. |
| Brain stem stroke syndrome | G663. |
| Cerebellar stroke syndrome | G664. |
| Pure motor lacunar syndrome | G665. |
| Pure sensory lacunar syndrome | G666. |
| Left sided CVA | G667. |
| Right sided CVA | G668. |
| Cerebral palsy, not congenital or infantile, acute | G669. |
| Cerebral atherosclerosis | G670. |
| Generalised ischaemic cerebrovascular disease NOS | G671. |
| Acute cerebrovascular insufficiency NOS | G6710 |
| Chronic cerebral ischaemia | G6711 |
| Generalised ischaemic cerebrovascular disease NOS | G671z |
| Moyamoya disease | G675. |
| Occlusion and stenosis of cerebral arteries, not resulting in cerebral infarction | G677. |
| Occlusion and stenosis of middle cerebral artery | G6770 |
| Occlusion and stenosis of anterior cerebral artery | G6771 |
| Occlusion and stenosis of posterior cerebral artery | G6772 |
| Occlusion and stenosis of cerebellar arteries | G6773 |
| Occlusion and stenosis of multiple and bilateral cerebral arteries | G6774 |
| Cerebral infarction due to unspecified occlusion or stenosis of precerebral arteries | G6W.. |
| Cerebral infarction due to unspecified occlusion or stenosis of cerebral arteries | G6X.. |
| Carotid artery atherosclerosis | G70y0 |

## Supplementary Table 3a. ICD-10 codes for diagnoses of ischaemic heart disease.

| **Description** | **Code** |
| --- | --- |
| Unstable angina | I200 |
| Angina pectoris with documented spasm | I201 |
| Other forms of angina pectoris | I208 |
| Angina pectoris unspecified | I209 |
| Acute transmural myocardial infarction of anterior wall | I210 |
| Acute transmural myocardial infarction of inferior wall | I211 |
| Acute transmural myocardial infarction of other sites | I212 |
| Acute transmural myocardial infarction of unspecified site | I213 |
| Acute subendocardial myocardial infarction | I214 |
| Acute myocardial infarction unspecified | I219 |
| Subsequent myocardial infarction of anterior wall | I220 |
| Subsequent myocardial infarction of inferior wall | I221 |
| Subsequent myocardial infarction of other sites | I228 |
| Subsequent myocardial infarction of unspecified site | I229 |
| Haemopericardium as curr comp folow acut myocard infarct | I230 |
| Atral sept defect as curr comp folow acut myocardal infarct | I231 |
| Ventric sep defect as curr comp fol acut myocardal infarc | I232 |
| Rup cardac wal withou haemopercard as cur comp fol ac MI | I233 |
| Rup chordae tendinae as curr comp fol acut myocard infarct | I234 |
| Rup papilary muscle as curr comp fol acute myocard infarct | I235 |
| Thromb atrium/auric append/vent as curr comp foll acute MI | I236 |
| Oth current comp following acute myocardial infarction | I238 |
| Coronary thrombosis not resulting in myocardial infarction | I240 |
| Dressler's syndrome | I241 |
| Other forms of acute ischaemic heart disease | I248 |
| Acute ischaemic heart diseaseunspecified | I249 |
| Atherosclerotic cardiovascular diseaseso described | I250 |
| Atherosclerotic heart disease | I251 |
| Old myocardial infarction | I252 |
| Silent myocardial ischaemia | I256 |
| Other forms of chronic ischaemic heart disease | I258 |
| Chronic ischaemic heart diseaseunspecified | I259 |

## Supplementary Table 3b. ICD-10 codes for diagnoses of peripheral arterial disease.

| **Description** | **Code** |
| --- | --- |
| Atherosclerosis of aorta | I700 |
| Atherosclerosis of renal artery | I701 |
| Atherosclerosis of arteries of extremities | I702 |
| Atherosclerosis of other arteries | I708 |
| Generalized and unspecified atherosclerosis | I709 |
| Dissection of aorta [any part] | I710 |
| Thoracic aortic aneurysm ruptured | I711 |
| Thoracic aortic aneurysm without mention of rupture | I712 |
| Abdominal aortic aneurysm ruptured | I713 |
| Abdominal aortic aneurysm without mention of rupture | I714 |
| Thoracoabdominal aortic aneurysm ruptured | I715 |
| Thoracoabdominal aortic aneurysm without mention of rupture | I716 |
| Aortic aneurysm of unspecified siter uptured | I718 |
| Aortic aneurysm of unspec site without mention of rupture | I719 |
| Aneurysm of carotid artery | I720 |
| Aneurysm of renal artery | I722 |
| Aneurysm of iliac artery | I723 |
| Aneurysm of artery of lower extremity | I724 |
| Aneurysm of other specified arteries | I728 |
| Aneurysm of unspecified site | I729 |
| Other specified peripheral vascular diseases | I738 |
| Peripheral vascular disease unspecified | I739 |

## Supplementary Table 3c. ICD-10 codes for diagnoses of stroke.

| **Description** | **Code** |
| --- | --- |
| Vertebro-basilar artery syndrome | G450 |
| Carotid artery syndrome (hemispheric) | G451 |
| Multiple and bilateral precerebral artery syndromes | G452 |
| Amaurosis fugax | G453 |
| Other transient cerebral ischaemic attacks and related synd | G458 |
| Transient cerebral ischaemic attack unspecified | G459 |
| Cerebral infarct due to thrombosis of precerebral arteries | I630 |
| Cerebral infarction due to embolism of precerebral arteries | I631 |
| Cereb infarct due unsp occlusion or stenos precerebrl arts | I632 |
| Cerebral infarction due to thrombosis of cerebral arteries | I633 |
| Cerebral infarction due to embolism of cerebral arteries | I634 |
| Cerebrl infarct due unspec occlusion or stenos cerebrl arts | I635 |
| Cereb infarct due cerebral venous thrombosisnonpyogenic | I636 |
| Other cerebral infarction | I638 |
| Cerebral infarction unspecified | I639 |
| Occlusion and stenosis of vertebral artery | I650 |
| Occlusion and stenosis of basilar artery | I651 |
| Occlusion and stenosis of carotid artery | I652 |
| Occlusion and stenosis of multip and bilat precerebrl arts | I653 |
| Occlusion and stenosis of other precerebral artery | I658 |
| Occlusion and stenosis of unspecified precerebral artery | I659 |
| Occlusion and stenosis of middle cerebral artery | I660 |
| Occlusion and stenosis of anterior cerebral artery | I661 |
| Occlusion and stenosis of posterior cerebral artery | I662 |
| Occlusion and stenosis of cerebellar arteries | I663 |
| Occlusion and stenosis of multiple and bilat cerebrl arts | I664 |
| Occlusion and stenosis of other cerebral artery | I668 |
| Occlusion and stenosis of unspecified cerebral artery | I669 |
| Sequelae of cerebral infarction | I693 |
| Sequelae of stroke not spec as haemorrhage or infarction | I694 |


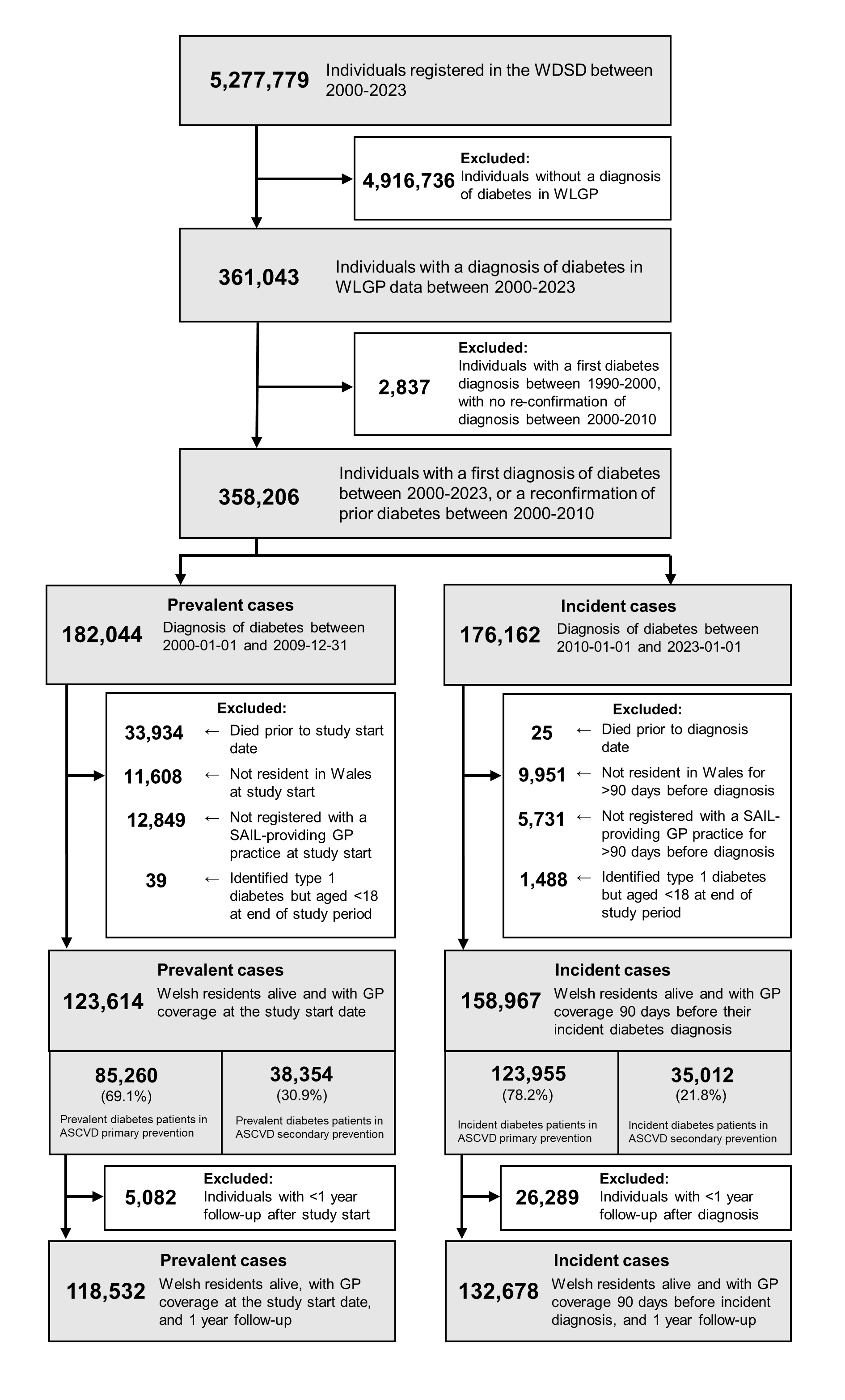


Supplementary Figure 1. Inclusion criteria for study cohort.

Supplementary Table 4. Cohort baseline additional characteristics at entry into study, continued.

|  | **Prevalent** | **Incident** | | | | | | | | | | | | | | **Overall** |
| --- | --- | --- | --- | --- | --- | --- | --- | --- | --- | --- | --- | --- | --- | --- | --- | --- |
|  |  | **2010** | **2011** | **2012** | **2013** | **2014** | **2015** | **2016** | **2017** | **2018** | **2019** | **2020** | **2021** | **2022** | **2023** |  |
|  | n = 123614 | n = 10922 | n = 10619 | n = 10459 | n = 10941 | n = 10632 | n = 11453 | n = 10253 | n = 10174 | n = 11072 | n = 12419 | n =  9740 | n = 12001 | n = 13764 | n = 14518 | n = 282581 |
| **Smoker status** | | | | | | | | | | | | | | | | |
| Active smoker | 21288 (17.2%) | 2300 (21.1%) | 2175 (20.5%) | 2262 (21.6%) | 2227 (20.4%) | 2268 (21.3%) | 2361 (20.6%) | 2116 (20.6%) | 2052 (20.2%) | 2205 (19.9%) | 2325 (18.7%) | 1957 (20.1%) | 2379 (19.8%) | 2539 (18.4%) | 2598 (17.9%) | 53052 (18.8%) |
| Ex-smoker | 64120 (51.9%) | 4879 (44.7%) | 4862 (45.8%) | 4751 (45.4%) | 5027 (45.9%) | 4958 (46.6%) | 5357 (46.8%) | 4735 (46.2%) | 4704 (46.2%) | 5161 (46.6%) | 5909 (47.6%) | 4479 (46%) | 5383 (44.9%) | 6318 (45.9%) | 6482 (44.6%) | 137125 (48.5%) |
| Non-smoker | 36839 (29.8%) | 3490 (32%) | 3415 (32.2%) | 3250 (31.1%) | 3499 (32%) | 3242 (30.5%) | 3539 (30.9%) | 3211 (31.3%) | 3220 (31.6%) | 3465 (31.3%) | 3937 (31.7%) | 3065 (31.5%) | 3899 (32.5%) | 4516 (32.8%) | 4992 (34.4%) | 87579 (31%) |
| Unknown | 1367 (1.1%) | 253 (2.3%) | 167 (1.6%) | 196 (1.9%) | 188 (1.7%) | 164 (1.5%) | 196 (1.7%) | 191 (1.9%) | 198 (1.9%) | 241 (2.2%) | 248 (2%) | 239 (2.5%) | 340 (2.8%) | 391 (2.8%) | 446 (3.1%) | 4825 (1.7%) |
| **Weight** | | | | | | | | | | | | | | | | |
| Obese | 63867 (51.7%) | 6326 (57.9%) | 6244 (58.8%) | 6252 (59.8%) | 6565 (60%) | 6263 (58.9%) | 6824 (59.6%) | 6109 (59.6%) | 6179 (60.7%) | 6766 (61.1%) | 7614 (61.3%) | 5981 (61.4%) | 7600 (63.3%) | 8406 (61.1%) | 8800 (60.6%) | 159796 (56.5%) |
| Overweight | 37058 (30%) | 2555 (23.4%) | 2494 (23.5%) | 2474 (23.7%) | 2515 (23%) | 2610 (24.5%) | 2688 (23.5%) | 2374 (23.2%) | 2303 (22.6%) | 2492 (22.5%) | 2798 (22.5%) | 2100 (21.6%) | 2450 (20.4%) | 3019 (21.9%) | 3140 (21.6%) | 73070 (25.9%) |
| Normal weight | 16443 (13.3%) | 845 (7.7%) | 832 (7.8%) | 769 (7.4%) | 930 (8.5%) | 843 (7.9%) | 956 (8.3%) | 812 (7.9%) | 822 (8.1%) | 851 (7.7%) | 996 (8%) | 779 (8%) | 843 (7%) | 1080 (7.8%) | 1103 (7.6%) | 28904 (10.2%) |
| Underweight | 1090 (0.9%) | 51 (0.5%) | 61 (0.6%) | 50 (0.5%) | 63 (0.6%) | 64 (0.6%) | 57 (0.5%) | 60 (0.6%) | 54 (0.5%) | 76 (0.7%) | 68 (0.5%) | 65 (0.7%) | 86 (0.7%) | 81 (0.6%) | 97 (0.7%) | 2023 (0.7%) |
| Unknown | 5156 (4.2%) | 1145 (10.5%) | 988 (9.3%) | 914 (8.7%) | 868 (7.9%) | 852 (8%) | 928 (8.1%) | 898 (8.8%) | 816 (8%) | 887 (8%) | 943 (7.6%) | 815 (8.4%) | 1022 (8.5%) | 1178 (8.6%) | 1378 (9.5%) | 18788 (6.6%) |
| **Welsh Index of Multiple Deprivation (WIMD) Quintile** | | | | | | | | | | | | | | | | |
| 1 | 27690 (22.4%) | 2519 (23.1%) | 2468 (23.2%) | 2361 (22.6%) | 2516 (23%) | 2404 (22.6%) | 2621 (22.9%) | 2393 (23.3%) | 2478 (24.4%) | 2596 (23.4%) | 2972 (23.9%) | 2278 (23.4%) | 2895 (24.1%) | 3228 (23.5%) | 3475 (23.9%) | 64894 (23%) |
| 2 | 27138 (22%) | 2374 (21.7%) | 2258 (21.3%) | 2316 (22.1%) | 2402 (22%) | 2392 (22.5%) | 2530 (22.1%) | 2192 (21.4%) | 2279 (22.4%) | 2422 (21.9%) | 2668 (21.5%) | 2132 (21.9%) | 2641 (22%) | 2995 (21.8%) | 3083 (21.2%) | 61822 (21.9%) |
| 3 | 24837 (20.1%) | 2227 (20.4%) | 2103 (19.8%) | 2113 (20.2%) | 2236 (20.4%) | 2218 (20.9%) | 2391 (20.9%) | 2125 (20.7%) | 2084 (20.5%) | 2290 (20.7%) | 2565 (20.7%) | 2018 (20.7%) | 2398 (20%) | 2794 (20.3%) | 2922 (20.1%) | 57321 (20.3%) |
| 4 | 22521 (18.2%) | 1912 (17.5%) | 1967 (18.5%) | 1887 (18%) | 1980 (18.1%) | 1990 (18.7%) | 2083 (18.2%) | 1893 (18.5%) | 1742 (17.1%) | 1967 (17.8%) | 2190 (17.6%) | 1714 (17.6%) | 2155 (18%) | 2448 (17.8%) | 2537 (17.5%) | 50986 (18%) |
| 5  or Unknown | 21428 (17.3%) | 1890 (17.3%) | 1823 (17.2%) | 1782 (17.0%) | 1807 (16.5%) | 1628 (15.3%) | 1828 (15.9%) | 1650 (16.0%) | 1591 (15.7%) | 1797 (16.2%) | 2024 (16.3%) | 1598 (16.4%) | 1912 (15.9%) | 2299 (16.7%) | 2501 (17.2%) | 47558 (16.9%) |

*Note:* Categorical descriptors are presented as the % of the cohort included in that category. Continuous descriptors are presented as mean(SD).

Supplementary Table 5. Cohort baseline characteristics at entry into study for diabetics without ASCVD.

|  | **Prevalent** | **Incident** | | | | | | | | | | | | | | **Overall** |
| --- | --- | --- | --- | --- | --- | --- | --- | --- | --- | --- | --- | --- | --- | --- | --- | --- |
|  |  | **2010** | **2011** | **2012** | **2013** | **2014** | **2015** | **2016** | **2017** | **2018** | **2019** | **2020** | **2021** | **2022** | **2023** |  |
|  | n = 85260 | n = 8471 | n = 8168 | n = 8092 | n = 8459 | n = 8086 | n = 8695 | n = 7947 | n = 7926 | n = 8684 | n = 9609 | n = 7669 | n = 9519 | n = 10907 | n = 11723 | n = 209215 |
| **Sex** | | | | | | | | | | | | | | | | |
| Male | 44678 (52.4%) | 4433 (52.3%) | 4285 (52.5%) | 4321 (53.4%) | 4341 (51.3%) | 4167 (51.5%) | 4418 (50.8%) | 4134 (52%) | 4105 (51.8%) | 4352 (50.1%) | 4865 (50.6%) | 3891 (50.7%) | 4818 (50.6%) | 5500 (50.4%) | 6013 (51.3%) | 108321 (51.8%) |
| **Age (years)** | | | | | | | | | | | | | | | | |
| Entry to study | 60.9 (15.8) | 56.7 (14.8) | 56.6 (14.8) | 56.4 (14.9) | 56.6 (14.8) | 56.5 (15.0) | 56.2 (15.3) | 55.1 (15.7) | 54.9 (15.7) | 55.5 (15.7) | 55.8 (15.9) | 54.7 (16.0) | 54.5 (15.5) | 55.5 (15.8) | 55.3 (15.9) | 57.8 (15.8) |
| **Comorbidities** | | | | | | | | | | | | | | | | |
| Respiratory Disease | 14571 (17.1%) | 1538 (18.2%) | 1524 (18.7%) | 1500 (18.5%) | 1679 (19.8%) | 1639 (20.3%) | 1771 (20.4%) | 1663 (20.9%) | 1704 (21.5%) | 1838 (21.2%) | 2158 (22.5%) | 1745 (22.8%) | 2089 (21.9%) | 2317 (21.2%) | 2575 (22%) | 40311 (19.3%) |
| Dementia | 942 (1.1%) | 52 (0.6%) | 54 (0.7%) | 52 (0.6%) | 45 (0.5%) | 35 (0.4%) | 59 (0.7%) | 58 (0.7%) | 40 (0.5%) | 49 (0.6%) | 71 (0.7%) | 68 (0.9%) | 45 (0.5%) | 63 (0.6%) | 66 (0.6%) | 1699 (0.8%) |
| CKD  (stage 3+)* | 11490 (13.5%) | 743 (8.8%) | 822 (10.1%) | 996 (12.3%) | 1181 (14%) | 1225 (15.1%) | 1304 (15%) | 1187 (14.9%) | 1303 (16.4%) | 1462 (16.8%) | 1808 (18.8%) | 1487 (19.4%) | 1775 (18.6%) | 2106 (19.3%) | 2282 (19.5%) | 31171 (14.9%) |
| Liver Disease | 1491 (1.7%) | 152 (1.8%) | 148 (1.8%) | 161 (2%) | 204 (2.4%) | 178 (2.2%) | 226 (2.6%) | 209 (2.6%) | 225 (2.8%) | 272 (3.1%) | 371 (3.9%) | 340 (4.4%) | 456 (4.8%) | 503 (4.6%) | 591 (5%) | 5527 (2.6%) |
| Heart Failure | 1491 (1.7%) | 102 (1.2%) | 101 (1.2%) | 85 (1.1%) | 82  (1.0%) | 101 (1.2%) | 136 (1.6%) | 95 (1.2%) | 107 (1.3%) | 113 (1.3%) | 135 (1.4%) | 94 (1.2%) | 171 (1.8%) | 147 (1.3%) | 151 (1.3%) | 3111 (1.5%) |
| Hypertension | 49492 (58%) | 3956 (46.7%) | 3745 (45.8%) | 3547 (43.8%) | 3648 (43.1%) | 3422 (42.3%) | 3589 (41.3%) | 3128 (39.4%) | 3072 (38.8%) | 3470 (40%) | 3932 (40.9%) | 2987 (38.9%) | 3575 (37.6%) | 4199 (38.5%) | 4480 (38.2%) | 100242 (47.9%) |
| **Smoker status** | | | | | | | | | | | | | | | | |
| Active smoker | 15124 (17.7%) | 1807 (21.3%) | 1699 (20.8%) | 1774 (21.9%) | 1717 (20.3%) | 1781 (22%) | 1823 (21%) | 1667 (21%) | 1617 (20.4%) | 1752 (20.2%) | 1830 (19%) | 1544 (20.1%) | 1850 (19.4%) | 1979 (18.1%) | 2070 (17.7%) | 40034 (19.1%) |
| Ex-smoker | 40543 (47.6%) | 3466 (40.9%) | 3425 (41.9%) | 3373 (41.7%) | 3596 (42.5%) | 3427 (42.4%) | 3731 (42.9%) | 3361 (42.3%) | 3371 (42.5%) | 3737 (43%) | 4186 (43.6%) | 3285 (42.8%) | 3988 (41.9%) | 4689 (43%) | 4940 (42.1%) | 93118 (44.5%) |
| Non-smoker | 28413 (33.3%) | 2974 (35.1%) | 2900 (35.5%) | 2773 (34.3%) | 2989 (35.3%) | 2731 (33.8%) | 2962 (34.1%) | 2742 (34.5%) | 2753 (34.7%) | 2978 (34.3%) | 3365 (35%) | 2622 (34.2%) | 3362 (35.3%) | 3870 (35.5%) | 4296 (36.6%) | 71730 (34.3%) |
| Unknown | 1180 (1.4%) | 224 (2.6%) | 144 (1.8%) | 172 (2.1%) | 157 (1.9%) | 147 (1.8%) | 179 (2.1%) | 177 (2.2%) | 185 (2.3%) | 217 (2.5%) | 228 (2.4%) | 218 (2.8%) | 319 (3.4%) | 369 (3.4%) | 417 (3.6%) | 4333 (2.1%) |
| **Weight** | | | | | | | | | | | | | | | | |
| Obese | 45065 (52.9%) | 4942 (58.3%) | 4868 (59.6%) | 4942 (61.1%) | 5159 (61%) | 4864 (60.2%) | 5259 (60.5%) | 4836 (60.9%) | 4896 (61.8%) | 5400 (62.2%) | 6054 (63%) | 4819 (62.8%) | 6125 (64.3%) | 6786 (62.2%) | 7238 (61.7%) | 121253 (58%) |
| Overweight | 24569 (28.8%) | 1902 (22.5%) | 1796 (22%) | 1786 (22.1%) | 1860 (22%) | 1872 (23.2%) | 1929 (22.2%) | 1728 (21.7%) | 1675 (21.1%) | 1816 (20.9%) | 1976 (20.6%) | 1552 (20.2%) | 1800 (18.9%) | 2219 (20.3%) | 2354 (20.1%) | 50834 (24.3%) |
| Normal weight | 10931 (12.8%) | 638 (7.5%) | 617 (7.6%) | 550 (6.8%) | 663 (7.8%) | 584 (7.2%) | 674 (7.8%) | 577 (7.3%) | 607 (7.7%) | 642 (7.4%) | 712 (7.4%) | 545 (7.1%) | 626 (6.6%) | 809 (7.4%) | 833 (7.1%) | 20008 (9.6%) |
| Underweight | 657 (0.8%) | 41 (0.5%) | 50 (0.6%) | 37 (0.5%) | 48 (0.6%) | 44 (0.5%) | 40 (0.5%) | 44 (0.6%) | 43 (0.5%) | 54 (0.6%) | 50 (0.5%) | 48 (0.6%) | 64 (0.7%) | 57 (0.5%) | 72 (0.6%) | 1349 (0.6%) |
| Unknown | 4038 (4.7%) | 948 (11.2%) | 837 (10.2%) | 777 (9.6%) | 729 (8.6%) | 722 (8.9%) | 793 (9.1%) | 762 (9.6%) | 705 (8.9%) | 772 (8.9%) | 817 (8.5%) | 705 (9.2%) | 904 (9.5%) | 1036 (9.5%) | 1226 (10.5%) | 15771 (7.5%) |
| **Welsh Index of Multiple Deprivation (WIMD) Quintile** | | | | | | | | | | | | | | | | |
| 1 | 18603 (21.8%) | 1929 (22.8%) | 1884 (23.1%) | 1834 (22.7%) | 1926 (22.8%) | 1830 (22.6%) | 2019 (23.2%) | 1866 (23.5%) | 1974 (24.9%) | 2049 (23.6%) | 2337 (24.3%) | 1785 (23.3%) | 2340 (24.6%) | 2574 (23.6%) | 2868 (24.5%) | 47818 (22.9%) |
| 2 | 18540 (21.7%) | 1823 (21.5%) | 1704 (20.9%) | 1778 (22%) | 1914 (22.6%) | 1835 (22.7%) | 1920 (22.1%) | 1700 (21.4%) | 1785 (22.5%) | 1889 (21.8%) | 2041 (21.2%) | 1691 (22%) | 2062 (21.7%) | 2411 (22.1%) | 2494 (21.3%) | 45587 (21.8%) |
| 3 | 17173 (20.1%) | 1730 (20.4%) | 1623 (19.9%) | 1624 (20.1%) | 1694 (20%) | 1657 (20.5%) | 1808 (20.8%) | 1643 (20.7%) | 1609 (20.3%) | 1786 (20.6%) | 1984 (20.6%) | 1560 (20.3%) | 1918 (20.1%) | 2198 (20.2%) | 2360 (20.1%) | 42367 (20.3%) |
| 4 | 15706 (18.4%) | 1497 (17.7%) | 1519 (18.6%) | 1456 (18%) | 1474 (17.4%) | 1487 (18.4%) | 1552 (17.8%) | 1454 (18.3%) | 1321 (16.7%) | 1544 (17.8%) | 1693 (17.6%) | 1348 (17.6%) | 1687 (17.7%) | 1903 (17.4%) | 2013 (17.2%) | 37654 (18%) |
| 5  or Unknown | 15238 (17.9%) | 1492 (17.6%) | 1438 (17.6%) | 1400 (17.3%) | 1451 (17.2%) | 1277 (15.8%) | 1396 (16.0%) | 1284 (16.2%) | 1237 (15.6%) | 1416 (16.3%) | 1554 (16.2%) | 1285 (16.8%) | 1512 (15.9%) | 1821 (16.7%) | 1988 (16.9%) | 35789 (17.1%) |

*Note:* Categorical descriptors are presented as the % of the cohort included in that category. Continuous descriptors are presented as mean(SD). *CKD stage 3+ captured as primary care diagnosis or eGFR <60.

Supplementary Table 6. Cohort baseline characteristics at entry into study for diabetics with ASCVD.

|  | **Prevalent** | **Incident** | | | | | | | | | | | | | | **Overall** |
| --- | --- | --- | --- | --- | --- | --- | --- | --- | --- | --- | --- | --- | --- | --- | --- | --- |
|  |  | **2010** | **2011** | **2012** | **2013** | **2014** | **2015** | **2016** | **2017** | **2018** | **2019** | **2020** | **2021** | **2022** | **2023** |  |
|  | n = 38354 | n = 2451 | n = 2451 | n = 2367 | n = 2482 | n = 2546 | n = 2758 | n = 2306 | n = 2248 | n = 2388 | n = 2810 | n = 2071 | n = 2482 | n = 2857 | n = 2795 | n = 73366 |
| **Sex** | | | | | | | | | | | | | | | | |
| Male | 22919 (59.8%) | 1550 (63.2%) | 1559 (63.6%) | 1503 (63.5%) | 1536 (61.9%) | 1557 (61.2%) | 1721 (62.4%) | 1431 (62.1%) | 1391 (61.9%) | 1523 (63.8%) | 1754 (62.4%) | 1336 (64.5%) | 1529 (61.6%) | 1878 (65.7%) | 1793 (64.2%) | 44980 (61.3%) |
| **Age (years)** | | | | | | | | | | | | | | | | |
| Entry to study | 71.6 (10.9) | 68.1 (11.3) | 68.1 (11.7) | 68.4 (11.6) | 69.0 (11.1) | 68.5 (11.5) | 69.4 (11.4) | 69.0 (11.7) | 68.7 (11.8) | 68.9 (11.9) | 69.6 (11.4) | 68.2 (11.9) | 67.9 (11.7) | 68.5 (11.8) | 68.9 (12.0) | 70.2 (11.3) |
| **Comorbidities** | | | | | | | | | | | | | | | | |
| Respiratory Disease | 8685 (22.6%) | 554 (22.6%) | 554 (22.6%) | 554 (23.4%) | 648 (26.1%) | 660 (25.9%) | 784 (28.4%) | 648 (28.1%) | 626 (27.8%) | 695 (29.1%) | 800 (28.5%) | 555 (26.8%) | 705 (28.4%) | 780 (27.3%) | 734 (26.3%) | 17982 (24.5%) |
| Dementia | 1268 (3.3%) | 43 (1.8%) | 50 (2.0%) | 53 (2.2%) | 62 (2.5%) | 55 (2.2%) | 71 (2.6%) | 42 (1.8%) | 62 (2.8%) | 53 (2.2%) | 75 (2.7%) | 56 (2.7%) | 51 (2.1%) | 67 (2.3%) | 77 (2.8%) | 2085 (2.8%) |
| CKD  (stage 3+)* | 11852 (30.9%) | 644 (26.3%) | 680 (27.7%) | 830 (35.1%) | 889 (35.8%) | 999 (39.2%) | 1123 (40.7%) | 918 (39.8%) | 952 (42.3%) | 1021 (42.8%) | 1330 (47.3%) | 938 (45.3%) | 1117 (45%) | 1292 (45.2%) | 1313 (47%) | 25898 (35.3%) |
| Liver Disease | 680 (1.8%) | 36 (1.5%) | 47 (1.9%) | 32 (1.4%) | 57 (2.3%) | 56 (2.2%) | 69 (2.5%) | 46 (2.0%) | 64 (2.8%) | 85 (3.6%) | 103 (3.7%) | 91 (4.4%) | 130 (5.2%) | 139 (4.9%) | 152 (5.4%) | 1787 (2.4%) |
| Heart Failure | 5252 (13.7%) | 283 (11.5%) | 276 (11.3%) | 251 (10.6%) | 301 (12.1%) | 295 (11.6%) | 361 (13.1%) | 263 (11.4%) | 275 (12.2%) | 256 (10.7%) | 303 (10.8%) | 256 (12.4%) | 262 (10.6%) | 318 (11.1%) | 331 (11.8%) | 9283 (12.7%) |
| Hypertension | 28130 (73.3%) | 1568 (64%) | 1520 (62%) | 1507 (63.7%) | 1586 (63.9%) | 1543 (60.6%) | 1656 (60%) | 1395 (60.5%) | 1333 (59.3%) | 1416 (59.3%) | 1701 (60.5%) | 1181 (57%) | 1409 (56.8%) | 1674 (58.6%) | 1610 (57.6%) | 49229 (67.1%) |
| **Smoker status** | | | | | | | | | | | | | | | | |
| Active smoker | 6164 (16.1%) | 493 (20.1%) | 476 (19.4%) | 488 (20.6%) | 510 (20.5%) | 487 (19.1%) | 538 (19.5%) | 449 (19.5%) | 435 (19.4%) | 453 (19%) | 495 (17.6%) | 413 (19.9%) | 529 (21.3%) | 560 (19.6%) | 528 (18.9%) | 13018 (17.7%) |
| Ex-smoker | 23577 (61.5%) | 1413 (57.6%) | 1437 (58.6%) | 1378 (58.2%) | 1431 (57.7%) | 1531 (60.1%) | 1626 (59%) | 1374 (59.6%) | 1333 (59.3%) | 1424 (59.6%) | 1723 (61.3%) | 1194 (57.7%) | 1395 (56.2%) | 1629 (57%) | 1542 (55.2%) | 44007 (60%) |
| Non-smoker | 8426 (22%) | 516 (21.1%) | 515 (21%) | 477 (20.2%) | 510 (20.5%) | 511 (20.1%) | 577 (20.9%) | 469 (20.3%) | 467 (20.8%) | 487 (20.4%) | 572 (20.4%) | 443 (21.4%) | 537 (21.6%) | 646 (22.6%) | 696 (24.9%) | 15849 (21.6%) |
| Unknown | 187 (0.5%) | 29 (1.2%) | 23 (0.9%) | 24 (1.0%) | 31 (1.2%) | 17 (0.7%) | 17 (0.6%) | 14 (0.6%) | 13 (0.6%) | 24 (1.0%) | 20 (0.7%) | 21 (1.0%) | 21 (0.8%) | 22 (0.8%) | 29 (1.0%) | 492 (0.7%) |
| **Weight** | | | | | | | | | | | | | | | | |
| Obese | 18802 (49%) | 1384 (56.5%) | 1376 (56.1%) | 1310 (55.3%) | 1406 (56.6%) | 1399 (54.9%) | 1565 (56.7%) | 1273 (55.2%) | 1283 (57.1%) | 1366 (57.2%) | 1560 (55.5%) | 1162 (56.1%) | 1475 (59.4%) | 1620 (56.7%) | 1562 (55.9%) | 38543 (52.5%) |
| Overweight | 12489 (32.6%) | 653 (26.6%) | 698 (28.5%) | 688 (29.1%) | 655 (26.4%) | 738 (29%) | 759 (27.5%) | 646 (28%) | 628 (27.9%) | 676 (28.3%) | 822 (29.3%) | 548 (26.5%) | 650 (26.2%) | 800 (28%) | 786 (28.1%) | 22236 (30.3%) |
| Normal weight | 5512 (14.4%) | 207 (8.4%) | 215 (8.8%) | 219 (9.3%) | 267 (10.8%) | 259 (10.2%) | 282 (10.2%) | 235 (10.2%) | 215 (9.6%) | 209 (8.8%) | 284 (10.1%) | 234 (11.3%) | 217 (8.7%) | 271 (9.5%) | 270 (9.7%) | 8896 (12.1%) |
| Underweight | 433 (1.1%) | 10 (0.4%) | 11 (0.4%) | 13 (0.5%) | 15 (0.6%) | 20 (0.8%) | 17 (0.6%) | 16 (0.7%) | 11 (0.5%) | 22 (0.9%) | 18 (0.6%) | 17 (0.8%) | 22 (0.9%) | 24 (0.8%) | 25 (0.9%) | 674 (0.9%) |
| Unknown | 1118 (2.9%) | 197 (8%) | 151 (6.2%) | 137 (5.8%) | 139 (5.6%) | 130 (5.1%) | 135 (4.9%) | 136 (5.9%) | 111 (4.9%) | 115 (4.8%) | 126 (4.5%) | 110 (5.3%) | 118 (4.8%) | 142 (5%) | 152 (5.4%) | 3017 (4.1%) |
| **Welsh Index of Multiple Deprivation (WIMD) Quintile** | | | | | | | | | | | | | | | | |
| 1 | 9087 (23.7%) | 590 (24.1%) | 584 (23.8%) | 527 (22.3%) | 590 (23.8%) | 574 (22.5%) | 602 (21.8%) | 527 (22.9%) | 504 (22.4%) | 547 (22.9%) | 635 (22.6%) | 493 (23.8%) | 555 (22.4%) | 654 (22.9%) | 607 (21.7%) | 17076 (23.3%) |
| 2 | 8598 (22.4%) | 551 (22.5%) | 554 (22.6%) | 538 (22.7%) | 488 (19.7%) | 557 (21.9%) | 610 (22.1%) | 492 (21.3%) | 494 (22%) | 533 (22.3%) | 627 (22.3%) | 441 (21.3%) | 579 (23.3%) | 584 (20.4%) | 589 (21.1%) | 16235 (22.1%) |
| 3 | 7664 (20%) | 497 (20.3%) | 480 (19.6%) | 489 (20.7%) | 542 (21.8%) | 561 (22%) | 583 (21.1%) | 482 (20.9%) | 475 (21.1%) | 504 (21.1%) | 581 (20.7%) | 458 (22.1%) | 480 (19.3%) | 596 (20.9%) | 562 (20.1%) | 14954 (20.4%) |
| 4 | 6815 (17.8%) | 415 (16.9%) | 448 (18.3%) | 431 (18.2%) | 506 (20.4%) | 503 (19.8%) | 531 (19.3%) | 439 (19%) | 421 (18.7%) | 423 (17.7%) | 497 (17.7%) | 366 (17.7%) | 468 (18.9%) | 545 (19.1%) | 524 (18.7%) | 13332 (18.2%) |
| 5  or Unknown | 6190 (16.1%) | 398 (16.2%) | 385 (15.7%) | 382 (16.2%) | 356 (14.3%) | 351 (13.7%) | 432 (15.6%) | 366 (15.8%) | 354 (15.8%) | 381 (15.9%) | 470 (16.7%) | 313 (15.1%) | 400 (16.1%) | 478 (16.8%) | 513 (18.3%) | 11769 (16%) |

*Note:* Categorical descriptors are presented as the % of the cohort included in that category. Continuous descriptors are presented as mean(SD). *CKD stage 3+ captured as primary care diagnosis or eGFR <60.

Supplementary Table 7. Prevalence and Incidence of diabetes mellitus by presence of ASCVD across the study period.

|  | **2010** | **2011** | **2012** | **2013** | **2014** | **2015** | **2016** | **2017** | **2018** | **2019** | **2020** | **2021** | **2022** | **2023** |
| --- | --- | --- | --- | --- | --- | --- | --- | --- | --- | --- | --- | --- | --- | --- |
| **Population denominator** | | | | | | | | | | | | | | |
|  | 2051664 | 2062311 | 2081386 | 2085283 | 2105066 | 2128019 | 2153403 | 2181306 | 2195894 | 2176005 | 2192949 | 2206006 | 2223173 | 2243170 |
| **Prevalence** | | | | | | | | | | | | | | |
| Overall | 133439 | 138875 | 143993 | 148810 | 153373 | 158635 | 161819 | 164681 | 167841 | 169081 | 171158 | 174147 | 178814 | 183948 |
| Without ASCVD | 90202 | 94049 | 97677 | 101047 | 104202 | 107785 | 110123 | 112198 | 114580 | 115705 | 117620 | 120344 | 124161 | 128308 |
| With ASCVD | 43237 | 44826 | 46316 | 47763 | 49171 | 50850 | 51696 | 52483 | 53261 | 53376 | 53538 | 53803 | 54653 | 55640 |
| **Prevalence (per 100,000)** | | | | | | | | | | | | | | |
| Overall | 6504 | 6734 | 6918 | 7136 | 7286 | 7455 | 7515 | 7550 | 7643 | 7770 | 7805 | 7894 | 8043 | 8200 |
| Without ASCVD | 4397 | 4560 | 4693 | 4846 | 4950 | 5065 | 5114 | 5144 | 5218 | 5317 | 5364 | 5455 | 5585 | 5720 |
| With ASCVD | 2107 | 2174 | 2225 | 2290 | 2336 | 2390 | 2401 | 2406 | 2425 | 2453 | 2441 | 2439 | 2458 | 2480 |
| **Incidence** | | | | | | | | | | | | | | |
| Overall | 11074 | 10778 | 10598 | 11060 | 10747 | 11567 | 10361 | 10250 | 11144 | 12484 | 9794 | 12029 | 13791 | 14539 |
| Without ASCVD | 8623 | 8327 | 8231 | 8578 | 8201 | 8809 | 8055 | 8002 | 8756 | 9674 | 7723 | 9547 | 10934 | 11744 |
| With ASCVD | 2451 | 2451 | 2367 | 2482 | 2546 | 2758 | 2306 | 2248 | 2388 | 2810 | 2071 | 2482 | 2857 | 2795 |
| **Incidence (per 100,000)** | | | | | | | | | | | | | | |
| Overall | 540 | 523 | 509 | 530 | 511 | 544 | 481 | 470 | 507 | 574 | 447 | 545 | 620 | 648 |
| Without ASCVD | 420 | 404 | 395 | 411 | 390 | 414 | 374 | 367 | 399 | 445 | 352 | 433 | 492 | 524 |
| With ASCVD | 119 | 119 | 114 | 119 | 121 | 130 | 107 | 103 | 109 | 129 | 94 | 113 | 129 | 125 |

Supplementary Table 8. Prescribed lipid lowering therapy across the study period, in those with or without ASCVD.

|  | **2010** | **2011** | **2012** | **2013** | **2014** | **2015** | **2016** | **2017** | **2018** | **2019** | **2020** | **2021** | **2022** | **2023** |
| --- | --- | --- | --- | --- | --- | --- | --- | --- | --- | --- | --- | --- | --- | --- |
| **Prevalent cases treated (each year) - Overall** | | | | | | | | | | | | | | |
| Overall | 117298 | 123014 | 127705 | 132234 | 137152 | 140947 | 145257 | 147870 | 147566 | 151038 | 154585 | 155740 | 158747 | 163945 |
| Combination statin | 5510 | 5228 | 4716 | 4089 | 3712 | 3424 | 3317 | 3205 | 3042 | 3072 | 3071 | 3254 | 3554 | 4131 |
| High-intensity statin | 9417 | 10374 | 10904 | 12476 | 14258 | 15892 | 17664 | 19223 | 20260 | 21831 | 23372 | 24640 | 26329 | 29097 |
| Non-high-intensity statin | 72426 | 74374 | 77190 | 78898 | 79566 | 78580 | 77676 | 76062 | 73262 | 72652 | 71699 | 71082 | 72040 | 73306 |
| Other treatment | 2649 | 2647 | 2454 | 2274 | 2270 | 2239 | 2146 | 2091 | 2010 | 1987 | 1996 | 1943 | 1990 | 2021 |
| No treatment | 27296 | 30391 | 32441 | 34497 | 37346 | 40812 | 44454 | 47289 | 48992 | 51496 | 54447 | 54821 | 54834 | 55390 |
| **Prevalent cases percentage treated (each year) - Overall** | | | | | | | | | | | | | | |
| Combination statin | 4.7 | 4.3 | 3.7 | 3.1 | 2.7 | 2.4 | 2.3 | 2.2 | 2.1 | 2.0 | 2.0 | 2.1 | 2.2 | 2.5 |
| High-intensity statin | 8.0 | 8.4 | 8.5 | 9.4 | 10.4 | 11.3 | 12.2 | 13.0 | 13.7 | 14.4 | 15.1 | 15.8 | 16.6 | 17.8 |
| Non-high-intensity statin | 61.8 | 60.5 | 60.4 | 59.7 | 58.0 | 55.8 | 53.5 | 51.4 | 49.6 | 48.1 | 46.4 | 45.6 | 45.4 | 44.7 |
| Other treatment | 2.3 | 2.1 | 1.9 | 1.7 | 1.7 | 1.6 | 1.5 | 1.4 | 1.4 | 1.3 | 1.3 | 1.3 | 1.3 | 1.2 |
| No treatment | 23.3 | 24.7 | 25.4 | 26.1 | 27.2 | 29.0 | 30.6 | 32.0 | 33.2 | 34.1 | 35.2 | 35.2 | 34.5 | 33.8 |
| **Incident cases treated (first year) - Overall** | | | | | | | | | | | | | | |
| Overall | 9339 | 10176 | 10008 | 10462 | 10120 | 10609 | 9721 | 9186 | 9892 | 10962 | 8669 | 10262 | 11739 | N/A |
| Combination statin | 204 | 232 | 198 | 172 | 133 | 119 | 116 | 123 | 114 | 128 | 105 | 126 | 183 | N/A |
| High-intensity statin | 462 | 520 | 557 | 739 | 836 | 1013 | 960 | 924 | 1065 | 1234 | 1036 | 1192 | 1639 | N/A |
| Non-high-intensity statin | 5029 | 5554 | 5413 | 5456 | 4995 | 4729 | 4004 | 3579 | 3707 | 4110 | 3065 | 3536 | 4385 | N/A |
| Other treatment | 85 | 85 | 70 | 79 | 72 | 69 | 60 | 57 | 70 | 87 | 49 | 77 | 68 | N/A |
| No treatment | 3559 | 3785 | 3770 | 4016 | 4084 | 4679 | 4581 | 4503 | 4936 | 5403 | 4414 | 5331 | 5464 | N/A |
| **Incident cases percentage treated (first year) - Overall** | | | | | | | | | | | | | | |
| Combination statin | 2.2 | 2.3 | 2.0 | 1.6 | 1.3 | 1.1 | 1.2 | 1.3 | 1.1 | 1.2 | 1.2 | 1.2 | 1.6 | N/A |
| High-intensity statin | 5.0 | 5.1 | 5.6 | 7.1 | 8.3 | 9.6 | 9.9 | 10.1 | 10.8 | 11.3 | 11.9 | 11.6 | 14.0 | N/A |
| Non-high-intensity statin | 53.9 | 54.6 | 54.1 | 52.1 | 49.4 | 44.6 | 41.2 | 39.0 | 37.5 | 37.5 | 35.4 | 34.5 | 37.4 | N/A |
| Other treatment | 0.9 | 0.8 | 0.7 | 0.8 | 0.7 | 0.7 | 0.6 | 0.6 | 0.7 | 0.8 | 0.6 | 0.8 | 0.6 | N/A |
| No treatment | 38.1 | 37.2 | 37.7 | 38.4 | 40.4 | 44.1 | 47.1 | 49.0 | 49.9 | 49.3 | 50.9 | 52.0 | 46.5 | N/A |
| **Prevalent cases treated (each year) - Without ASCVD** | | | | | | | | | | | | | | |
| Overall | 79624 | 83828 | 87134 | 90434 | 94034 | 96695 | 99723 | 101690 | 101613 | 104310 | 107584 | 108692 | 111097 | 115046 |
| Combination statin | 2913 | 2715 | 2424 | 2008 | 1817 | 1642 | 1596 | 1527 | 1442 | 1436 | 1423 | 1499 | 1648 | 1842 |
| High-intensity statin | 4673 | 5088 | 5237 | 6103 | 6899 | 7670 | 8378 | 8906 | 9179 | 9713 | 10328 | 10773 | 11512 | 12896 |
| Non-high-intensity statin | 47727 | 49111 | 50987 | 52352 | 53034 | 52285 | 51609 | 50823 | 49153 | 49291 | 49258 | 49436 | 50827 | 52578 |
| Other treatment | 1724 | 1746 | 1570 | 1455 | 1456 | 1410 | 1335 | 1300 | 1232 | 1225 | 1205 | 1176 | 1187 | 1219 |
| No treatment | 22587 | 25168 | 26916 | 28516 | 30828 | 33688 | 36805 | 39134 | 40607 | 42645 | 45370 | 45808 | 45923 | 46511 |
| **Prevalent cases percentage treated (each year) - Without ASCVD** | | | | | | | | | | | | | | |
| Combination statin | 3.7 | 3.2 | 2.8 | 2.2 | 1.9 | 1.7 | 1.6 | 1.5 | 1.4 | 1.4 | 1.3 | 1.4 | 1.5 | 1.6 |
| High-intensity statin | 5.9 | 6.1 | 6.0 | 6.8 | 7.3 | 7.9 | 8.4 | 8.8 | 9.0 | 9.3 | 9.6 | 9.9 | 10.4 | 11.2 |
| Non-high-intensity statin | 59.9 | 58.6 | 58.5 | 57.9 | 56.4 | 54.1 | 51.8 | 50.0 | 48.4 | 47.3 | 45.8 | 45.5 | 45.8 | 45.7 |
| Other treatment | 2.2 | 2.1 | 1.8 | 1.6 | 1.6 | 1.5 | 1.3 | 1.3 | 1.2 | 1.2 | 1.1 | 1.1 | 1.1 | 1.1 |
| No treatment | 28.4 | 30.0 | 30.9 | 31.5 | 32.8 | 34.8 | 36.9 | 38.5 | 40.0 | 40.9 | 42.2 | 42.1 | 41.3 | 40.4 |
| **Incident cases treated (first year) - Without ASCVD** | | | | | | | | | | | | | | |
| Overall | 7216 | 7868 | 7787 | 8128 | 7731 | 8082 | 7548 | 7206 | 7774 | 8541 | 6805 | 8148 | 9260 | N/A |
| Combination statin | 94 | 124 | 82 | 90 | 68 | 57 | 62 | 58 | 55 | 52 | 52 | 61 | 83 | N/A |
| High-intensity statin | 165 | 184 | 227 | 287 | 316 | 419 | 364 | 356 | 391 | 425 | 345 | 432 | 622 | N/A |
| Non-high-intensity statin | 3566 | 4008 | 3929 | 3951 | 3527 | 3263 | 2844 | 2531 | 2668 | 2953 | 2249 | 2642 | 3393 | N/A |
| Other treatment | 51 | 44 | 39 | 49 | 34 | 37 | 35 | 27 | 46 | 47 | 30 | 46 | 38 | N/A |
| No treatment | 3340 | 3508 | 3510 | 3751 | 3786 | 4306 | 4243 | 4234 | 4614 | 5064 | 4129 | 4967 | 5124 | N/A |
| **Incident cases percentage treated (first year) - Without ASCVD** | | | | | | | | | | | | | | |
| Combination statin | 1.3 | 1.6 | 1.1 | 1.1 | 0.9 | 0.7 | 0.8 | 0.8 | 0.7 | 0.6 | 0.8 | 0.8 | 0.9 | N/A |
| High-intensity statin | 2.3 | 2.3 | 2.9 | 3.5 | 4.1 | 5.2 | 4.8 | 4.9 | 5.0 | 5.0 | 5.1 | 5.3 | 6.7 | N/A |
| Non-high-intensity statin | 49.4 | 50.9 | 50.5 | 48.6 | 45.6 | 40.4 | 37.7 | 35.1 | 34.3 | 34.6 | 33.0 | 32.4 | 36.6 | N/A |
| Other treatment | 0.7 | 0.6 | 0.5 | 0.6 | 0.4 | 0.5 | 0.5 | 0.4 | 0.6 | 0.6 | 0.4 | 0.6 | 0.4 | N/A |
| No treatment | 46.3 | 44.6 | 45.1 | 46.1 | 49.0 | 53.3 | 56.2 | 58.8 | 59.4 | 59.3 | 60.7 | 61.0 | 55.3 | N/A |
| **Prevalent cases treated (each year) - With ASCVD** | | | | | | | | | | | | | | |
| Overall | 37674 | 39186 | 40571 | 41800 | 43118 | 44252 | 45534 | 46180 | 45953 | 46728 | 47001 | 47048 | 47650 | 48899 |
| Combination statin | 2597 | 2513 | 2292 | 2081 | 1895 | 1782 | 1721 | 1678 | 1600 | 1636 | 1648 | 1755 | 1906 | 2289 |
| High-intensity statin | 4744 | 5286 | 5667 | 6373 | 7359 | 8222 | 9286 | 10317 | 11081 | 12118 | 13044 | 13867 | 14817 | 16201 |
| Non-high-intensity statin | 24699 | 25263 | 26203 | 26546 | 26532 | 26295 | 26067 | 25239 | 24109 | 23361 | 22441 | 21646 | 21213 | 20728 |
| Other treatment | 925 | 901 | 884 | 819 | 814 | 829 | 811 | 791 | 778 | 762 | 791 | 767 | 803 | 802 |
| No treatment | 4709 | 5223 | 5525 | 5981 | 6518 | 7124 | 7649 | 8155 | 8385 | 8851 | 9077 | 9013 | 8911 | 8879 |
| **Prevalent cases percentage treated (each year) - With ASCVD** | | | | | | | | | | | | | | |
| Combination statin | 6.9 | 6.4 | 5.7 | 5.0 | 4.4 | 4.0 | 3.8 | 3.6 | 3.5 | 3.5 | 3.5 | 3.7 | 4.0 | 4.7 |
| High-intensity statin | 12.6 | 13.5 | 14.0 | 15.3 | 17.1 | 18.6 | 20.4 | 22.3 | 24.1 | 25.9 | 27.8 | 29.5 | 31.1 | 33.1 |
| Non-high-intensity statin | 65.6 | 64.5 | 64.6 | 63.5 | 61.5 | 59.4 | 57.3 | 54.6 | 52.5 | 50.0 | 47.8 | 46.0 | 44.5 | 42.4 |
| Other treatment | 2.5 | 2.3 | 2.2 | 2.0 | 1.9 | 1.9 | 1.8 | 1.7 | 1.7 | 1.6 | 1.7 | 1.6 | 1.7 | 1.6 |
| No treatment | 12.5 | 13.3 | 13.6 | 14.3 | 15.1 | 16.1 | 16.8 | 17.7 | 18.3 | 18.9 | 19.3 | 19.2 | 18.7 | 18.2 |
| **Incident cases treated (first year) - With ASCVD** | | | | | | | | | | | | | | |
| Overall | 2123 | 2308 | 2221 | 2334 | 2389 | 2527 | 2173 | 1980 | 2118 | 2421 | 1864 | 2114 | 2479 | N/A |
| Combination statin | 110 | 108 | 116 | 82 | 65 | 62 | 54 | 65 | 59 | 76 | 53 | 65 | 100 | N/A |
| High-intensity statin | 297 | 336 | 330 | 452 | 520 | 594 | 596 | 568 | 674 | 809 | 691 | 760 | 1017 | N/A |
| Non-high-intensity statin | 1463 | 1546 | 1484 | 1505 | 1468 | 1466 | 1160 | 1048 | 1039 | 1157 | 816 | 894 | 992 | N/A |
| Other treatment | 34 | 41 | 31 | 30 | 38 | 32 | 25 | 30 | 24 | 40 | 19 | 31 | 30 | N/A |
| No treatment | 219 | 277 | 260 | 265 | 298 | 373 | 338 | 269 | 322 | 339 | 285 | 364 | 340 | N/A |
| **Incident cases percentage treated (first year) - With ASCVD** | | | | | | | | | | | | | | |
| Combination statin | 5.2 | 4.7 | 5.2 | 3.5 | 2.7 | 2.5 | 2.5 | 3.3 | 2.8 | 3.1 | 2.8 | 3.1 | 4.0 | N/A |
| High-intensity statin | 14.0 | 14.6 | 14.9 | 19.4 | 21.8 | 23.5 | 27.4 | 28.7 | 31.8 | 33.4 | 37.1 | 36.0 | 41.0 | N/A |
| Non-high-intensity statin | 68.9 | 67.0 | 66.8 | 64.5 | 61.5 | 58.0 | 53.4 | 52.9 | 49.1 | 47.8 | 43.8 | 42.3 | 40.0 | N/A |
| Other treatment | 1.6 | 1.8 | 1.4 | 1.3 | 1.6 | 1.3 | 1.1 | 1.5 | 1.1 | 1.6 | 1.0 | 1.5 | 1.2 | N/A |
| No treatment | 10.3 | 12.0 | 11.7 | 11.3 | 12.5 | 14.8 | 15.6 | 13.6 | 15.2 | 14.0 | 15.3 | 17.2 | 13.7 | N/A |

Supplementary Table 9. Multivariable logistic regression analysis of variables associated with a prescription for high-intensity statin therapy in diabetics with ASCVD within the incident year.

|  | Odds ratio | Lower CI | Upper CI | P |
| --- | --- | --- | --- | --- |
| Sex – Female (reference) |  |  |  |  |
| Sex – Male | 1.35 | 1.26 | 1.45 | <0.01 |
| Diagnosis year | 1.14 | 1.13 | 1.15 | <0.01 |
| Age at diagnosis | 0.98 | 0.97 | 0.98 | <0.01 |
| Weight – Normal (reference) |  |  |  |  |
| Weight – Obese | 1.01 | 0.89 | 1.14 | 0.90 |
| Weight – Overweight | 1.12 | 0.98 | 1.27 | 0.10 |
| Weight – Unknown | 0.96 | 0.80 | 1.17 | 0.71 |
| Weight – Underweight | 0.88 | 0.51 | 1.47 | 0.65 |
| Smoker Status – Non-smoker (reference) |  |  |  |  |
| Smoker Status – Ex-smoker | 1.19 | 1.09 | 1.30 | <0.01 |
| Smoker Status – Current smoker | 1.28 | 1.15 | 1.43 | <0.01 |
| Smoker Status – Unknown | 1.04 | 0.70 | 1.53 | 0.84 |
| Dementia | 0.70 | 0.52 | 0.93 | 0.02 |
| Respiratory Disease | 0.91 | 0.85 | 0.98 | 0.02 |
| Liver Disease | 0.69 | 0.56 | 0.83 | <0.01 |
| Chronic kidney disease (stage 3+) | 0.91 | 0.82 | 1.00 | 0.05 |
| Hypertension | 0.86 | 0.80 | 0.92 | <0.01 |

Model covariates determined by minimising the Akaike information criterion – covariates removed include: Welsh Index of Multiple Deprivation (WIMD) quintile.

Supplementary Table 10. Number and proportion of diabetics with ASCVD with documented and controlled low density lipoprotein cholesterol by year.

|  | **2010** | **2011** | **2012** | **2013** | **2014** | **2015** | **2016** | **2017** | **2018** | **2019** | **2020** | **2021** | **2022** | **2023** |
| --- | --- | --- | --- | --- | --- | --- | --- | --- | --- | --- | --- | --- | --- | --- |
| **Prevalent cases (each year)** | | | | | | | | | | | | | | |
| Tested - Controlled (<1.8 mmol/L) | 10642 | 11100 | 10939 | 11423 | 12638 | 13897 | 13851 | 14297 | 14967 | 15448 | 12934 | 13536 | 15606 | 16810 |
| Tested - Not controlled (≥1.8 mmol/L) | 15322 | 15889 | 16784 | 16551 | 17155 | 18335 | 18703 | 17721 | 16186 | 16166 | 12671 | 13071 | 16036 | 15371 |
| Not tested | 10943 | 11484 | 12112 | 13043 | 12581 | 11171 | 12089 | 13294 | 13926 | 14279 | 20813 | 19679 | 15111 | 15792 |
| **Prevalent cases percentage tested (each year)** | | | | | | | | | | | | | | |
| Tested - Controlled (<1.8 mmol/L) | 28.8 | 28.9 | 27.5 | 27.8 | 29.8 | 32 | 31 | 31.6 | 33.2 | 33.7 | 27.9 | 29.2 | 33.4 | 35.0 |
| Tested - Not controlled (≥1.8 mmol/L) | 41.5 | 41.3 | 42.1 | 40.4 | 40.5 | 42.2 | 41.9 | 39.1 | 35.9 | 35.2 | 27.3 | 28.2 | 34.3 | 32.0 |
| Not tested | 29.7 | 29.8 | 30.4 | 31.8 | 29.7 | 25.7 | 27.1 | 29.3 | 30.9 | 31.1 | 44.8 | 42.5 | 32.3 | 32.9 |
| **Incident cases (first year)** | | | | | | | | | | | | | | |
| Tested - Controlled (<1.8 mmol/L) | 625 | 538 | 472 | 550 | 599 | 664 | 516 | 529 | 583 | 613 | 445 | 516 | 738 | N/A |
| Tested - Not controlled (≥1.8 mmol/L) | 1042 | 1068 | 1028 | 1031 | 1122 | 1146 | 867 | 798 | 827 | 836 | 523 | 773 | 842 | N/A |
| Not tested | 788 | 849 | 874 | 895 | 845 | 945 | 914 | 929 | 987 | 1360 | 1116 | 1203 | 1287 | N/A |
| **Incident cases percentage tested (first year)** | | | | | | | | | | | | | | |
| Tested - Controlled (<1.8 mmol/L) | 25.5 | 21.9 | 19.9 | 22.2 | 23.3 | 24.1 | 22.5 | 23.4 | 24.3 | 21.8 | 21.4 | 20.7 | 25.7 | N/A |
| Tested - Not controlled (≥1.8 mmol/L) | 42.4 | 43.5 | 43.3 | 41.6 | 43.7 | 41.6 | 37.7 | 35.4 | 34.5 | 29.8 | 25.1 | 31 | 29.4 | N/A |
| Not tested | 32.1 | 34.6 | 36.8 | 36.1 | 32.9 | 34.3 | 39.8 | 41.2 | 41.2 | 48.4 | 53.6 | 48.3 | 44.9 | N/A |


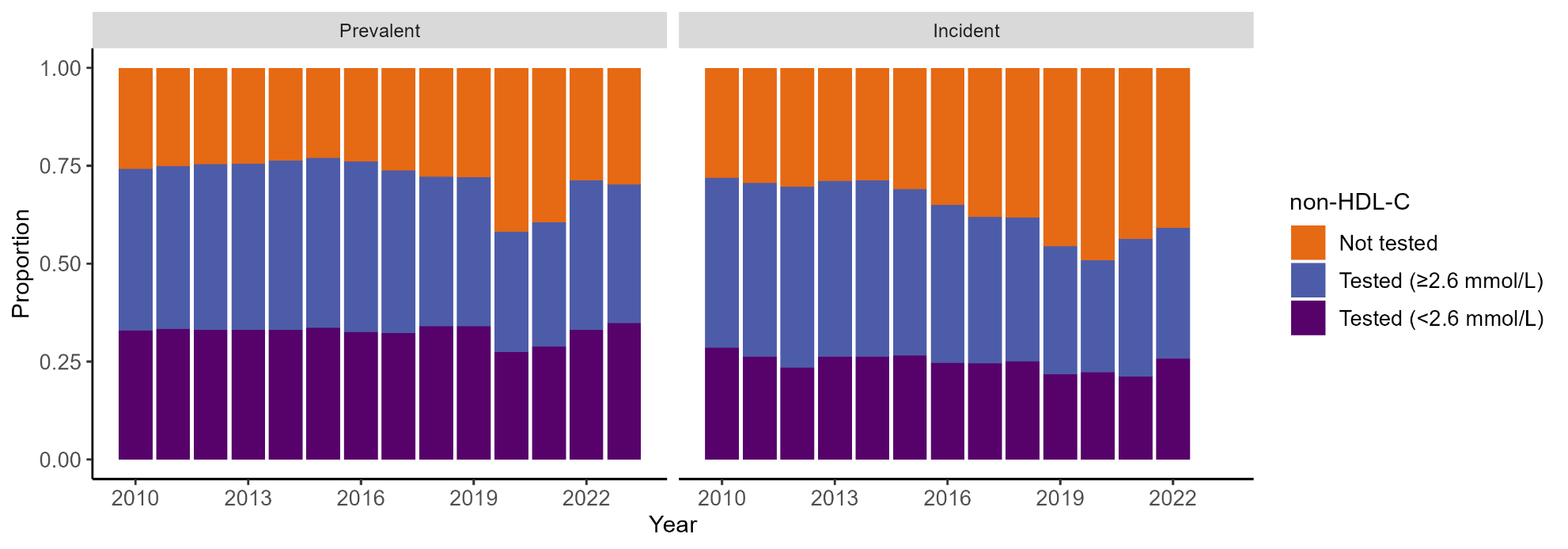


Supplementary Figure 2. Proportion of diabetics with ASCVD with documented and controlled non-high density lipoprotein cholesterol by year.

Supplementary Table 11. Proportion of diabetics with ASCVD with documented and controlled non-high density lipoprotein cholesterol by year.

|  | **2010** | **2011** | **2012** | **2013** | **2014** | **2015** | **2016** | **2017** | **2018** | **2019** | **2020** | **2021** | **2022** | **2023** |
| --- | --- | --- | --- | --- | --- | --- | --- | --- | --- | --- | --- | --- | --- | --- |
| **Prevalent cases (each year)** | | | | | | | | | | | | | | |
| Tested - Controlled (<2.6 mmol/L) | 12131 | 12814 | 13150 | 13572 | 14010 | 14584 | 14505 | 14627 | 15323 | 15594 | 12716 | 13295 | 15443 | 16668 |
| Tested - Not controlled (≥2.6 mmol/L) | 15256 | 15939 | 16865 | 17355 | 18247 | 18820 | 19413 | 18806 | 17239 | 17438 | 14270 | 14739 | 17804 | 16985 |
| Not tested | 9501 | 9674 | 9768 | 10023 | 10025 | 9955 | 10690 | 11838 | 12495 | 12823 | 19400 | 18207 | 13446 | 14255 |
| **Prevalent cases percentage tested (each year)** | | | | | | | | | | | | | | |
| Tested - Controlled (<2.6 mmol/L) | 32.9 | 33.3 | 33.1 | 33.1 | 33.1 | 33.6 | 32.5 | 32.3 | 34 | 34 | 27.4 | 28.8 | 33.1 | 34.8 |
| Tested - Not controlled (≥2.6 mmol/L) | 41.4 | 41.5 | 42.4 | 42.4 | 43.2 | 43.4 | 43.5 | 41.5 | 38.3 | 38 | 30.8 | 31.9 | 38.1 | 35.5 |
| Not tested | 25.8 | 25.2 | 24.6 | 24.5 | 23.7 | 23 | 24 | 26.1 | 27.7 | 28 | 41.8 | 39.4 | 28.8 | 29.8 |
| **Incident cases (first year)** | | | | | | | | | | | | | | |
| Tested - Controlled (<2.6 mmol/L) | 699 | 646 | 559 | 651 | 672 | 732 | 567 | 554 | 601 | 611 | 463 | 528 | 739 | N/A |
| Tested - Not controlled (≥2.6 mmol/L) | 1066 | 1087 | 1097 | 1109 | 1156 | 1168 | 923 | 841 | 878 | 917 | 595 | 873 | 958 | N/A |
| Not tested | 689 | 721 | 721 | 715 | 736 | 855 | 803 | 859 | 915 | 1278 | 1022 | 1088 | 1171 | N/A |
| **Incident cases percentage tested (first year)** | | | | | | | | | | | | | | |
| Tested - Controlled (<2.6 mmol/L) | 28.5 | 26.3 | 23.5 | 26.3 | 26.2 | 26.6 | 24.7 | 24.6 | 25.1 | 21.8 | 22.3 | 21.2 | 25.8 | N/A |
| Tested - Not controlled (≥2.6 mmol/L) | 43.4 | 44.3 | 46.2 | 44.8 | 45.1 | 42.4 | 40.3 | 37.3 | 36.7 | 32.7 | 28.6 | 35.1 | 33.4 | N/A |
| Not tested | 28.1 | 29.4 | 30.3 | 28.9 | 28.7 | 31 | 35 | 38.1 | 38.2 | 45.5 | 49.1 | 43.7 | 40.8 | N/A |

Supplementary Table 12. Multivariable logistic regression model to predict the likelihood of achieving low density lipoprotein cholesterol control (<1.8 mmol/L) in diabetics with ASCVD during incident year.

|  | Odds ratio | Lower CI | Upper CI | P |
| --- | --- | --- | --- | --- |
| Sex – Female (reference) |  |  |  |  |
| Sex – Male | 1.35 | 1.26 | 1.44 | <0.01 |
| Diagnosis year | 1.01 | 1.00 | 1.02 | 0.03 |
| Age at diagnosis | 1.01 | 1.01 | 1.01 | <0.01 |
| Smoker Status – Non-smoker (reference) |  |  |  |  |
| Smoker Status – Ex-smoker | 1.03 | 0.95 | 1.12 | 0.42 |
| Smoker Status – Current smoker | 0.90 | 0.82 | 1.00 | 0.06 |
| Smoker Status – Unknown | 1.26 | 0.85 | 1.85 | 0.24 |
| Dementia | 0.74 | 0.57 | 0.95 | 0.02 |
| Liver Disease | 1.19 | 0.99 | 1.43 | 0.06 |
| Chronic kidney disease (stage 3+) | 1.10 | 1.01 | 1.20 | 0.03 |
| Lipid lowering therapy (LLT) – No treatment (reference) |  |  |  |  |
| LLT – Other treatment | 1.04 | 0.60 | 1.69 | 0.88 |
| LLT – Non-high-intensity statin | 7.57 | 6.47 | 8.92 | <0.01 |
| LLT – High-intensity statin | 9.30 | 7.90 | 11.01 | <0.01 |
| LLT – Combination statin | 7.58 | 6.06 | 9.49 | <0.01 |

Model covariates determined by minimising the Akaike information criterion – covariates removed include: Welsh Index of Multiple Deprivation (WIMD) quintile, Weight, Respiratory Disease, Hypertension

Supplementary Table 13. Prescribed lipid lowering therapy across the study period in diabetics with chronic kidney disease (stage 3+) and without ASCVD.

|  | **2010** | **2011** | **2012** | **2013** | **2014** | **2015** | **2016** | **2017** | **2018** | **2019** | **2020** | **2021** | **2022** | **2023** |
| --- | --- | --- | --- | --- | --- | --- | --- | --- | --- | --- | --- | --- | --- | --- |
| **Prevalent cases treated (each year)** | | | | | | | | | | | | | | |
| Overall | 10402 | 10125 | 9883 | 9775 | 9951 | 10028 | 10211 | 10306 | 10295 | 10686 | 11232 | 11469 | 11982 | 12838 |
| Combination statin | 523 | 451 | 385 | 292 | 278 | 225 | 202 | 183 | 167 | 174 | 168 | 191 | 203 | 213 |
| High-intensity statin | 700 | 672 | 647 | 679 | 716 | 770 | 815 | 847 | 860 | 896 | 953 | 991 | 1084 | 1273 |
| Non-high-intensity statin | 6633 | 6314 | 6159 | 6069 | 6062 | 5903 | 5732 | 5630 | 5448 | 5541 | 5682 | 5811 | 6157 | 6677 |
| Other treatment | 356 | 341 | 296 | 267 | 255 | 226 | 216 | 206 | 192 | 181 | 172 | 149 | 152 | 162 |
| No treatment | 2190 | 2347 | 2396 | 2468 | 2640 | 2904 | 3246 | 3440 | 3628 | 3894 | 4257 | 4327 | 4386 | 4513 |
| **Prevalent cases % treated (each year)** | | | | | | | | | | | | | | |
| Combination statin | 5.0 | 4.5 | 3.9 | 3.0 | 2.8 | 2.2 | 2.0 | 1.8 | 1.6 | 1.6 | 1.5 | 1.7 | 1.7 | 1.7 |
| High-intensity statin | 6.7 | 6.6 | 6.5 | 7.0 | 7.2 | 7.7 | 8.0 | 8.2 | 8.3 | 8.4 | 8.5 | 8.6 | 9.1 | 9.9 |
| Non-high-intensity statin | 63.8 | 62.4 | 62.3 | 62.1 | 60.9 | 58.9 | 56.1 | 54.6 | 52.9 | 51.9 | 50.6 | 50.7 | 51.4 | 52.0 |
| Other treatment | 3.4 | 3.4 | 3.0 | 2.7 | 2.6 | 2.3 | 2.1 | 2.0 | 1.9 | 1.7 | 1.5 | 1.3 | 1.3 | 1.3 |
| No treatment | 21.1 | 23.2 | 24.2 | 25.3 | 26.5 | 29.0 | 31.8 | 33.4 | 35.2 | 36.4 | 37.9 | 37.7 | 36.6 | 35.1 |
| **Incident cases treated (first year)** | | | | | | | | | | | | | | |
| Overall | 625 | 762 | 958 | 1114 | 1154 | 1232 | 1133 | 1154 | 1327 | 1603 | 1297 | 1558 | 1840 | N/A |
| Combination statin | 15 | 10 | 11 | 30 | 12 | 13 | 14 | 15 | 23 | 16 | 15 | 11 | 18 | N/A |
| High-intensity statin | 11 | 21 | 33 | 37 | 63 | 74 | 72 | 60 | 69 | 80 | 71 | 96 | 140 | N/A |
| Non-high-intensity statin | 342 | 425 | 531 | 602 | 612 | 599 | 522 | 540 | 569 | 726 | 564 | 645 | 907 | N/A |
| Other treatment | 10 | 10 | 10 | 10 | 13 | 11 | 14 | 10 | 14 | 16 | 12 | 16 | 14 | N/A |
| No treatment | 247 | 296 | 373 | 435 | 454 | 535 | 511 | 529 | 652 | 765 | 635 | 790 | 761 | N/A |
| **Incident cases % treated (first year)** | | | | | | | | | | | | | | |
| Combination statin | 2.4 | 1.3 | 1.1 | 2.7 | 1.0 | 1.1 | 1.2 | 1.3 | 1.7 | 1.0 | 1.2 | 0.7 | 1.0 | N/A |
| High-intensity statin | 1.8 | 2.8 | 3.4 | 3.3 | 5.5 | 6.0 | 6.3 | 5.2 | 5.2 | 5.0 | 5.5 | 6.2 | 7.6 | N/A |
| Non-high-intensity statin | 54.7 | 55.8 | 55.4 | 54.0 | 53.0 | 48.6 | 46.1 | 46.8 | 42.9 | 45.3 | 43.5 | 41.4 | 49.3 | N/A |
| Other treatment | 1.6 | 1.3 | 1.0 | 0.9 | 1.1 | 0.9 | 1.2 | 0.9 | 1.1 | 1.0 | 0.9 | 1.0 | 0.8 | N/A |
| No treatment | 39.5 | 38.9 | 38.9 | 39.0 | 39.3 | 43.4 | 45.1 | 45.8 | 49.1 | 47.7 | 49.0 | 50.7 | 41.4 | N/A |

Supplementary Table 14. Multivariable logistic regression model to predict the likelihood of being prescribed lipid lowering therapy to diabetics with chronic kidney disease (stage 3+) and without ASCVD during incident year.

|  | Odds ratio | Lower CI | Upper CI | P |
| --- | --- | --- | --- | --- |
| Sex – Female (reference) |  |  |  |  |
| Sex – Male | 1.12 | 1.04 | 1.21 | <0.01 |
| Welsh Index of Multiple Deprivation (WIMD) quintile – 1 (reference) Most deprived |  |  |  |  |
| WIMD quintile – 2 | 0.95 | 0.85 | 1.08 | 0.45 |
| WIMD quintile – 3 | 0.78 | 0.69 | 0.88 | <0.01 |
| WIMD quintile – 4 | 0.85 | 0.75 | 0.96 | 0.01 |
| WIMD quintile – 5 (least deprived) | 0.80 | 0.70 | 0.91 | <0.01 |
| WIMD quintile - Unknown | 1.07 | 0.27 | 4.55 | 0.92 |
| Diagnosis year | 0.98 | 0.97 | 0.99 | <0.01 |
| Age at diagnosis | 1.01 | 1.00 | 1.01 | <0.01 |
| Weight – Normal (reference) |  |  |  |  |
| Weight - Obese | 1.49 | 1.28 | 1.72 | <0.01 |
| Weight - Overweight | 1.45 | 1.24 | 1.69 | <0.01 |
| Weight - Unknown | 0.89 | 0.72 | 1.11 | 0.29 |
| Weight - Underweight | 0.28 | 0.13 | 0.56 | <0.01 |
| Smoker Status – Non-smoker (reference) |  |  |  |  |
| Smoker Status – Ex-smoker | 1.09 | 1.00 | 1.19 | 0.05 |
| Smoker Status – Current smoker | 1.39 | 1.22 | 1.58 | <0.01 |
| Smoker Status – Unknown | 0.73 | 0.48 | 1.10 | 0.14 |
| Dementia | 0.66 | 0.49 | 0.90 | 0.01 |
| Liver Disease | 0.81 | 0.65 | 0.99 | 0.04 |
| Hypertension | 1.96 | 1.80 | 2.13 | <0.01 |

Model covariates determined by minimising the Akaike information criterion – covariates removed include: Respiratory Disease
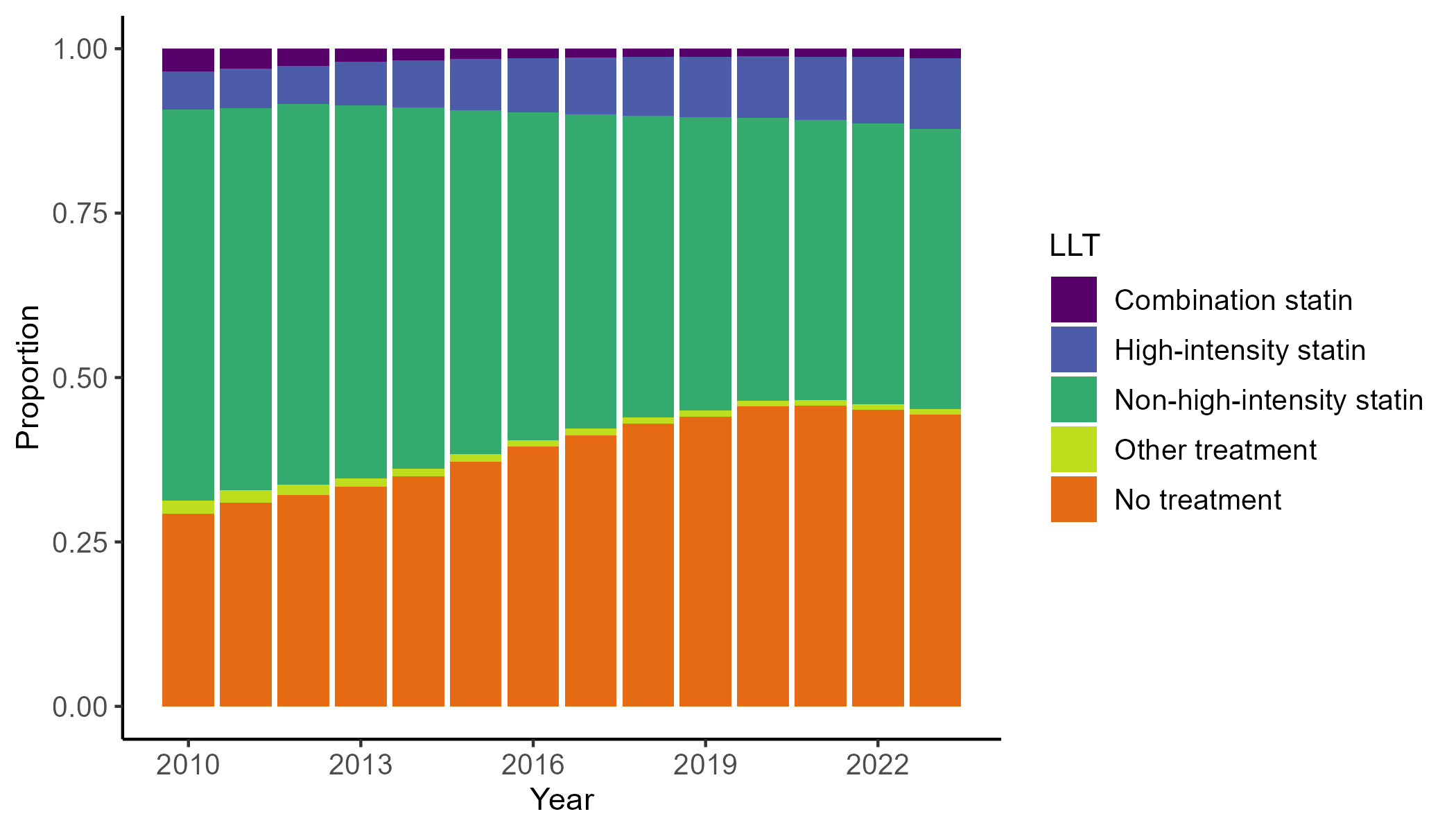


Supplementary Figure 3. Prescribed lipid lowering therapy for prevalent diabetics (without chronic kidney disease (stage 3+) or ASCVD) across the study period.

Supplementary Table 15. Prescribed lipid lowering therapy for prevalent diabetics (without chronic kidney disease (stage 3+) or ASCVD) across the study period.

|  | **2010** | **2011** | **2012** | **2013** | **2014** | **2015** | **2016** | **2017** | **2018** | **2019** | **2020** | **2021** | **2022** | **2023** |
| --- | --- | --- | --- | --- | --- | --- | --- | --- | --- | --- | --- | --- | --- | --- |
| **Prevalent cases treated (each year)** | | | | | | | | | | | | | | |
| Overall | 70156 | 72581 | 72418 | 71318 | 72678 | 73449 | 74965 | 75863 | 74176 | 75093 | 76503 | 76703 | 78155 | 80168 |
| Combination statin | 2417 | 2203 | 1851 | 1446 | 1245 | 1109 | 1063 | 1028 | 945 | 923 | 905 | 922 | 1008 | 1130 |
| High-intensity statin | 4060 | 4354 | 4241 | 4692 | 5234 | 5746 | 6216 | 6549 | 6589 | 6860 | 7159 | 7355 | 7844 | 8681 |
| Non-high-intensity statin | 41750 | 42154 | 41878 | 40454 | 39909 | 38429 | 37319 | 36263 | 34066 | 33541 | 32925 | 32692 | 33432 | 34139 |
| Other treatment | 1396 | 1395 | 1150 | 924 | 904 | 839 | 780 | 752 | 699 | 665 | 638 | 622 | 625 | 622 |
| No treatment | 20533 | 22475 | 23298 | 23802 | 25386 | 27326 | 29587 | 31271 | 31877 | 33104 | 34876 | 35112 | 35246 | 35596 |
| **Prevalent cases % treated (each year)** | | | | | | | | | | | | | | |
| Combination statin | 3.5 | 3.0 | 2.6 | 2.0 | 1.7 | 1.5 | 1.4 | 1.4 | 1.3 | 1.2 | 1.2 | 1.2 | 1.3 | 1.4 |
| High-intensity statin | 5.8 | 6.0 | 5.9 | 6.6 | 7.2 | 7.8 | 8.3 | 8.6 | 8.9 | 9.1 | 9.4 | 9.6 | 10.0 | 10.8 |
| Non-high-intensity statin | 59.5 | 58.1 | 57.8 | 56.7 | 54.9 | 52.3 | 49.8 | 47.8 | 45.9 | 44.7 | 43.0 | 42.6 | 42.8 | 42.6 |
| Other treatment | 2.0 | 1.9 | 1.6 | 1.3 | 1.2 | 1.1 | 1.0 | 1.0 | 0.9 | 0.9 | 0.8 | 0.8 | 0.8 | 0.8 |
| No treatment | 29.3 | 31.0 | 32.2 | 33.4 | 34.9 | 37.2 | 39.5 | 41.2 | 43.0 | 44.1 | 45.6 | 45.8 | 45.1 | 44.4 |

Supplementary Table 16. Number and proportion of diabetics (not prescribed lipid lowering therapy prior to diagnosis and without chronic kidney disease (stage 3+) or ASCVD) with documented QRISK 10-year risk score by QRISK threshold and year.

|  | **2010** | **2011** | **2012** | **2013** | **2014** | **2015** | **2016** | **2017** | **2018** | **2019** | **2020** | **2021** | **2022** | **2023** |
| --- | --- | --- | --- | --- | --- | --- | --- | --- | --- | --- | --- | --- | --- | --- |
| **Incident cases documented** | | | | | | | | | | | | | | |
| Not recorded | 4130 | 4564 | 4449 | 4471 | 4141 | 3993 | 3528 | 3349 | 3317 | 3575 | 2966 | 3192 | 3118 | N/A |
| Below 10% | 52 | 56 | 71 | 90 | 112 | 185 | 262 | 290 | 338 | 428 | 318 | 456 | 581 | N/A |
| Between 10-19% | 73 | 102 | 102 | 99 | 176 | 293 | 366 | 392 | 510 | 536 | 434 | 622 | 837 | N/A |
| Over 20% | 184 | 224 | 191 | 228 | 249 | 468 | 585 | 564 | 659 | 687 | 490 | 771 | 940 | N/A |
| **Incident cases % documented** | | | | | | | | | | | | | | |
| Not recorded | 93.0 | 92.3 | 92.4 | 91.5 | 88.5 | 80.8 | 74.4 | 72.9 | 68.8 | 68.4 | 70.5 | 63.3 | 56.9 | N/A |
| Below 10% | 1.2 | 1.1 | 1.5 | 1.8 | 2.4 | 3.7 | 5.5 | 6.3 | 7.0 | 8.2 | 7.6 | 9.0 | 10.6 | N/A |
| Between 10-19% | 1.6 | 2.1 | 2.1 | 2.0 | 3.8 | 5.9 | 7.7 | 8.5 | 10.6 | 10.3 | 10.3 | 12.3 | 15.3 | N/A |
| Over 20% | 4.1 | 4.5 | 4.0 | 4.7 | 5.3 | 9.5 | 12.3 | 12.3 | 13.7 | 13.1 | 11.6 | 15.3 | 17.2 | N/A |

Supplementary Table 17. Multivariable logistic regression model to predict the likelihood of having QRISK score documented for diabetics (not prescribed lipid lowering therapy prior to diagnosis and without chronic kidney disease (stage 3+) or ASCVD) during incident year.

|  | Odds ratio | Lower CI | Upper CI | P |
| --- | --- | --- | --- | --- |
| Sex – Female (reference) |  |  |  |  |
| Sex – Male | 1.25 | 1.20 | 1.30 | <0.01 |
| Welsh Index of Multiple Deprivation (WIMD) quintile – 1 (reference) |  |  |  |  |
| WIMD quintile – 2 | 1.08 | 1.02 | 1.15 | 0.01 |
| WIMD quintile – 3 | 1.04 | 0.98 | 1.10 | 0.23 |
| WIMD quintile – 4 | 1.24 | 1.16 | 1.32 | <0.01 |
| WIMD quintile – 5 | 1.07 | 1.00 | 1.14 | 0.04 |
| WIMD quintile - Unknown | 0.88 | 0.46 | 1.67 | 0.70 |
| Diagnosis year | 1.07 | 1.06 | 1.07 | <0.01 |
| Age at diagnosis | 1.02 | 1.02 | 1.02 | <0.01 |
| Weight – Normal (reference) |  |  |  |  |
| Weight - Obese | 2.33 | 2.16 | 2.51 | <0.01 |
| Weight - Overweight | 1.67 | 1.54 | 1.82 | <0.01 |
| Weight - Unknown | 1.00 | 0.91 | 1.10 | 0.98 |
| Weight - Underweight | 0.57 | 0.43 | 0.75 | 0.00 |
| Smoker Status – Non-smoker (reference) |  |  |  |  |
| Smoker Status – Ex-smoker | 1.14 | 1.09 | 1.19 | <0.01 |
| Smoker Status – Current smoker | 1.08 | 1.02 | 1.14 | 0.01 |
| Smoker Status - Unknown | 0.55 | 0.49 | 0.62 | <0.01 |
| Dementia | 0.34 | 0.24 | 0.49 | <0.01 |
| Respiratory Disease | 0.91 | 0.86 | 0.96 | <0.01 |
| Liver Disease | 1.25 | 1.10 | 1.42 | <0.01 |
| Hypertension | 1.36 | 1.29 | 1.42 | <0.01 |

Model covariates determined by minimising the Akaike information criterion – no covariates removed.

Supplementary Table 18. Prescribed lipid lowering therapy for incident diabetics (not prescribed lipid lowering therapy prior to diagnosis and without chronic kidney disease (stage 3+) or ASCVD) in the year following diagnosis by QRISK documentation and year.

|  | **2010** | **2011** | **2012** | **2013** | **2014** | **2015** | **2016** | **2017** | **2018** | **2019** | **2020** | **2021** | **2022** | **2023** |
| --- | --- | --- | --- | --- | --- | --- | --- | --- | --- | --- | --- | --- | --- | --- |
| **Incident cases - No QRISK documented** | | | | | | | | | | | | | | |
| No treatment | 2902 | 2958 | 2891 | 3015 | 3015 | 3251 | 2961 | 2885 | 2967 | 3161 | 2627 | 3018 | 2903 | N/A |
| Statin or other treatment | 1257 | 1603 | 1527 | 1433 | 1168 | 831 | 594 | 460 | 383 | 369 | 298 | 297 | 345 | N/A |
| **Incident cases - No QRISK documented (%)** | | | | | | | | | | | | | | |
| No treatment | 69.8 | 64.9 | 65.4 | 67.8 | 72.1 | 79.6 | 83.3 | 86.2 | 88.6 | 89.5 | 89.8 | 91 | 89.4 | N/A |
| Statin or other treatment | 30.2 | 35.1 | 34.6 | 32.2 | 27.9 | 20.4 | 16.7 | 13.8 | 11.4 | 10.5 | 10.2 | 9 | 10.6 | N/A |
| **Incident cases - Below 10%** | | | | | | | | | | | | | | |
| No treatment | 35 | 37 | 48 | 68 | 81 | 141 | 224 | 249 | 291 | 364 | 272 | 382 | 471 | N/A |
| Statin or other treatment | 11 | 10 | 30 | 22 | 35 | 39 | 37 | 38 | 50 | 60 | 54 | 61 | 84 | N/A |
| **Incident cases - Below 10% (%)** | | | | | | | | | | | | | | |
| No treatment | 76.1 | 78.7 | 61.5 | 75.6 | 69.8 | 78.3 | 85.8 | 86.8 | 85.3 | 85.8 | 83.4 | 86.2 | 84.9 | N/A |
| Statin or other treatment | 23.9 | 21.3 | 38.5 | 24.4 | 30.2 | 21.7 | 14.2 | 13.2 | 14.7 | 14.2 | 16.6 | 13.8 | 15.1 | N/A |
| **Incident cases - Between 10-19%** | | | | | | | | | | | | | | |
| No treatment | 42 | 52 | 62 | 67 | 96 | 155 | 229 | 247 | 326 | 337 | 293 | 386 | 490 | N/A |
| Statin or other treatment | 21 | 48 | 45 | 53 | 57 | 115 | 125 | 146 | 158 | 216 | 160 | 204 | 274 | N/A |
| **Incident cases - Between 10-19% (%)** | | | | | | | | | | | | | | |
| No treatment | 66.7 | 52 | 57.9 | 55.8 | 62.7 | 57.4 | 64.7 | 62.8 | 67.4 | 60.9 | 64.7 | 65.4 | 64.1 | N/A |
| Statin or other treatment | 33.3 | 48 | 42.1 | 44.2 | 37.3 | 42.6 | 35.3 | 37.2 | 32.6 | 39.1 | 35.3 | 34.6 | 35.9 | N/A |
| **Incident cases - Over 20%** | | | | | | | | | | | | | | |
| No treatment | 63 | 108 | 98 | 121 | 96 | 183 | 282 | 285 | 333 | 397 | 272 | 366 | 453 | N/A |
| Statin or other treatment | 108 | 130 | 112 | 109 | 130 | 224 | 289 | 285 | 316 | 322 | 232 | 327 | 456 | N/A |
| **Incident cases - Over 20% (%)** | | | | | | | | | | | | | | |
| No treatment | 36.8 | 45.4 | 46.7 | 52.6 | 42.5 | 45 | 49.4 | 50 | 51.3 | 55.2 | 54 | 52.8 | 49.8 | N/A |
| Statin or other treatment | 63.2 | 54.6 | 53.3 | 47.4 | 57.5 | 55 | 50.6 | 50 | 48.7 | 44.8 | 46 | 47.2 | 50.2 | N/A |

Supplementary Table 19. Multivariable logistic regression model to predict the likelihood of being prescribed lipid lowering therapy in the year following diagnosis for incident diabetics (not prescribed lipid lowering therapy prior to diagnosis and without chronic kidney disease (stage 3+) or ASCVD).

|  | | Odds ratio | Lower CI | Upper CI | P |
| --- | --- | --- | --- | --- | --- |
| Sex – Female (reference) |  | |  |  |  |
| Sex – Male | 1.38 | | 1.33 | 1.43 | <0.01 |
| Welsh Index of Multiple Deprivation (WIMD) quintile – 1 (reference) most deprived |  | |  |  |  |
| WIMD quintile - 2 | | 0.89 | 0.85 | 0.94 | <0.01 |
| WIMD quintile - 3 | | 0.77 | 0.73 | 0.82 | <0.01 |
| WIMD quintile - 4 | | 0.82 | 0.77 | 0.86 | <0.01 |
| WIMD quintile – 5 (least deprived) | | 0.77 | 0.73 | 0.82 | <0.01 |
| WIMD quintile - Unknown | | 0.51 | 0.26 | 0.95 | 0.04 |
| Diagnosis year | | 0.92 | 0.92 | 0.93 | <0.01 |
| Age at diagnosis | | 1.04 | 1.04 | 1.04 | <0.01 |
| Weight – Normal (reference) | |  |  |  |  |
| Weight - Obese | | 1.69 | 1.57 | 1.82 | <0.01 |
| Weight - Overweight | | 1.64 | 1.51 | 1.78 | <0.01 |
| Weight - Unknown | | 1.14 | 1.04 | 1.26 | 0.01 |
| Weight - Underweight | | 0.40 | 0.27 | 0.56 | <0.01 |
| Smoker Status – Non-smoker (reference) | |  |  |  |  |
| Smoker Status – Ex-smoker | | 1.18 | 1.13 | 1.23 | <0.01 |
| Smoker Status – Current smoker | | 1.59 | 1.52 | 1.67 | <0.01 |
| Smoker Status - Unknown | | 0.76 | 0.66 | 0.88 | <0.01 |
| Dementia | | 0.68 | 0.50 | 0.91 | 0.01 |
| Respiratory Disease | | 0.96 | 0.92 | 1.00 | 0.06 |
| Hypertension | | 1.91 | 1.84 | 1.98 | <0.01 |
| Documented QRISK | | 1.59 | 1.52 | 1.66 | <0.01 |

Model covariates determined by minimising the Akaike information criterion – covariates removed include: Liver Disease.
